# Global burden of early-onset colorectal cancer related to alcohol, tobacco, and physical inactivity: evidence from the global burden of disease 2021

**DOI:** 10.3389/fonc.2026.1653676

**Published:** 2026-04-21

**Authors:** Tianyu Ma, Binbin Hu, Dongli Zhou

**Affiliations:** Cancer Center, Gaoyou People’s Hospital, Gaoyou, Jiangsu, China

**Keywords:** early-onset colorectal cancer, high alcohol use, tobacco, low physical activity, predication

## Abstract

**Objective:**

To investigate global trends in early-onset colorectal cancer attributable to behavioral risk factors (high alcohol use, tobacco, and low physical activity).

**Methods:**

Data on early-onset colorectal cancer were obtained from the Global Burden of Disease 2021 database. Epidemiological tendencies were evaluated. Correlation between socio-demographic index and age-standardized rates was evaluated using the Pearson correlation coefficient. Bayesian age-period-cohort models were to predict disease burden by 2050.

**Results:**

From 1990 to 2021, the global burden of early-onset colorectal cancer attributable to the three behavioral risk factors declined overall. The most decrease in disease burden caused by tobacco was in the high socio-demographic index regions. The decrease of burden was the most in Europe. Fractions of disease burden attributable to high alcohol use and tobacco were higher in males, whereas the opposite pattern was observed for low physical activity. The disease burden was increased with age, reaching the highest level in individuals aged 45–49 years. In addition, disease burden was positively correlated with socio-demographic index. Projections indicated that by 2050, the disease burdens attributable to high alcohol use and tobacco were expected to decline, while the burden associated with low physical activity was relatively stable.

**Conclusions:**

Over the past three decades, the global burden of early-onset colorectal cancer attributable to three behavioral risk factors exhibits an overall downward trend. Targeted prevention and management strategies should particularly focus on individuals aged 45-49.

## 1 Introduction

Colorectal cancer (CRC), the second leading cause of cancer-related deaths worldwide, accounts for approximately 9% of all cancer-related deaths (1, 2). According to data from the Global Cancer Observatory in 2022, more than 900,000 deaths are attributed to CRC annually (3). Over the past three decades, countries with high social-demographic index (SDI) have experienced declines in both age-standardized incidence rate (ASIR) and age-standardized mortality rate (ASMR) of CRC (4). In contrast, countries with low-middle SDI level have experienced a significant increase in CRC incidence (5). Recent studies have reported a global rise in the incidence of early-onset CRC, defined as CRC diagnosed in individuals under 50 years of age, accounting for 10%-12% of all CRC cases (6–8). Patients with early-onset CRC often have insufficient awareness of symptoms and lack knowledge of early CRC screening, which may contribute to delayed diagnosis and disease progression (9).

The incidence of CRC is closely related to adverse behavior risk factors, particularly alcohol consumption, tobacco use and low physical activity, which are considered modifiable risk factors (10–12). However, the global burden of early-onset CRC related to these behavioral risk factors has not been fully explored. According to the Global Burden of Disease (GBD) 2019 study, the global number of CRC deaths attributable to tobacco and alcohol consumption increased to 142,931 and 52,495, respectively, in 2019 (13). Notably, mortality from CRC associated with alcohol and tobacco has shown an increasing trend (13). In Middle East and North Africa, the burden of CRC due to low physical activity has shown a declining trend over the past 30 years (10). However, it remains unclear whether these established behavioral risk factors exert a similar influence on early-onset CRC. Identifying these risk factors is crucial for improving prevention strategies and promoting early screening for early-onset CRC.

The disease trend of early-onset CRC is jointly determined by multiple factors (behavior, diet, and metabolism). This study aims to investigate the global, regional, and national burden of early-onset CRC attributable to three behavioral risk factors (high alcohol use, tobacco, and low physical activity). The findings of this study may provide evidence to support targeted early interventions aimed at reducing the disease burden associated with these risk factors.

## 2 Methods

### 2.1 Data source

The data from 1990 to 2021 on early-onset CRC attributable to high alcohol use, tobacco and low physical activity were retrieved from the GBD database using the Global Health Data Exchange (GHDx) query tool (http://ghdx.healthdata.org/), including mortality, disability-adjusted life years (DALYs), and their corresponding age-standardized rates (ASRs). The GBD database provided early-onset CRC data at global, regional and national levels. A total of 204 countries and territories were grouped into five levels: low, low-middle, middle, high-middle, and high SDI regions (14). The clinical definition of early-onset CRC was diagnosed under the age of 50 years (15). Age was classified into seven subgroups: 15-19, 20-24, 25-29, 30-34, 35-39, 40–44 and 45–49 years. Ethical approval and informed consent were not required as the GBD data were publicly accessible and the analyses did not involve any identifiable information. All data in the GBD study complied with the Guidelines for Accurate and Transparent Health Estimates Reporting (GATHER) for population health research (16).

### 2.2 Risk factors

The disease burden attributable to each risk factor was estimated based on the comparative risk assessment (CRA) framework in GBD 2021. The CRA approach quantified the disease burden attributable to a specific risk factor by comparing the observed distribution of exposure to that risk factor in the target population with a counterfactual scenario defined by the Theoretical Minimum Risk Exposure Level (TMREL). The difference in disease burden between these two scenarios represented the proportion of burden that can be attributed to the risk factor. To ensure the validity, robustness, and comparability of attribution estimates, the CRA framework used in the GBD study was based on the assumption of a plausible TMREL. This assumption ensured that the estimated attributable burden represented the maximum potentially avoidable burden that could be achieved through population-level control and reduction of risk factor exposure. In this study, top three behavioral risk factors, which contributed to early-onset CRC mortality and DALYs, were selected, including high alcohol use, tobacco and low physical activity.

### 2.3 Statistical analysis

Trends in the ASR-related indicators of early-onset CRC attributable to high alcohol use, tobacco and low physical activity were presented with 95% uncertainty interval (UI). The temporal tendencies of early-onset CRC in mortality and DALYs were assessed using the estimated annual percentage change (EAPC), describing with 95% confidence interval (CI). The EAPC was calculated as: 100 × [exp (β)-1], indicating the annual percentage change. EAPC >0: the increase of ASRs. EAPC = 0: the stable of ASRs. EAPC <0: the decrease of ASRs (17). The correlations between ASMR, age-standardized DALYs rate and SDI in different GBD regions were evaluated using the Pearson correlation coefficient. A significant difference was *P* < 0.05. The future burden tendencies were forecasted by a Bayesian age-period-cohort (BAPC) model over the next 30 years (18). The BAPC hierarchical model was employed to simultaneously assess the nonlinear effects of age, calendar period, and birth cohort, thereby providing robust projections. Model fitting was performed using the Integrated Nested Laplace Approximations (INLA) package (version 24.05.10). A two-level convergence criterion was applied to ensure the reliability of model inference. The 95% UIs derived from the marginal posterior predictive distributions of the parameters were used to quantify the uncertainty in parameter estimates. All statistical analyses and visualizations were performed using R software (version 4.4.1).

## 3 Results

### 3.1 Global trends of early-onset CRC attributable to three behavioral risk factors

Globally, the number of deaths from early-onset CRC-related attributable to high alcohol use increased by 1.34 times between 1990 and 2021. Yet, the ASMR declined during the same period (**Table 1**). A similar downward trend was observed in the age-standardized DALYs rate from 1990 to 2021 (**Table 1**). In terms of early-onset CRC caused by tobacco, our results indicated that both deaths and DALYs reduced over the study period (**Table 2**). Moreover, a declining trend was observed in early-onset CRC attributable to low physical activity (**Table 3**). These findings revealed that the burdens of deaths and DALYs in early-onset CRC attributable to three behavioral risk factors were decreased over the past three decades.

**Table 1:** Global and regional trends in the burden of early-onset colorectal cancer attributable to high alcohol use: deaths and disability-adjusted life years (1990–2021).

Location name	1990		2021		EAPC (95% CI)
	Number	ASR	Number	ASR	
Deaths					
Global	3947 (3058-4878)	0.146 (0.113-0.18)	5277 (4046-6682)	0.134 (0.102-0.169)	-0.33 (-0.44 to -0.23)
SDI regions					
High SDI	1387 (1106-1715)	0.301 (0.24-0.372)	1197 (943-1489)	0.238 (0.188-0.296)	-0.86 (-0.94 to -0.79)
High-middle SDI	1388 (1075-1714)	0.246 (0.19-0.304)	1601 (1217-2099)	0.254 (0.193-0.333)	-0.11 (-0.23 to 0)
Middle SDI	990 (705-1272)	0.109 (0.077-0.14)	1898 (1444-2452)	0.151 (0.115-0.195)	1.2 (0.97 to 1.43)
Low-middle SDI	123 (84-168)	0.022 (0.015-0.031)	414 (304-549)	0.041 (0.03-0.054)	2.3 (2.18 to 2.41)
Low SDI	54 (25-77)	0.024 (0.011-0.035)	162 (106-230)	0.03 (0.02-0.042)	0.55 (0.27 to 0.83)
Continents					
Africa	98 (55-131)	0.034 (0.019-0.046)	278 (186-396)	0.041 (0.028-0.059)	0.53 (0.4 to 0.65)
America	573 (457-719)	0.156 (0.124-0.196)	977 (781-1230)	0.191 (0.152-0.24)	0.71 (0.64 to 0.78)
Asia	1990 (1437-2502)	0.12 (0.087-0.151)	3074 (2324-4097)	0.13 (0.098-0.173)	0.29 (0.04 to 0.55)
Europe	1278 (1021-1576)	0.32 (0.256-0.395)	940 (739-1169)	0.244 (0.192-0.304)	-1.12 (-1.31 to -0.93)
WHO regions					
African Region	94 (51-127)	0.041 (0.022-0.056)	272 (181-389)	0.049 (0.032-0.07)	0.47 (0.34 to 0.6)
Eastern Mediterranean Region	9 (6-13)	0.005 (0.004-0.008)	31 (22-44)	0.008 (0.005-0.011)	1.63 (1.39 to 1.86)
European Region	1312 (1048-1623)	0.308 (0.246-0.381)	977 (769-1215)	0.228 (0.18-0.284)	-1.22 (-1.41 to -1.03)
Region of the Americas	573 (457-719)	0.156 (0.124-0.196)	977 (781-1230)	0.191 (0.152-0.24)	0.71 (0.64 to 0.78)
South-East Asia Region	148 (94-208)	0.023 (0.015-0.032)	453 (332-597)	0.04 (0.029-0.053)	1.86 (1.78 to 1.94)
Western Pacific Region	1772 (1275-2246)	0.208 (0.149-0.263)	2526 (1871-3409)	0.278 (0.206-0.376)	0.98 (0.65 to 1.31)
Global burden of disease regions					
East Asia	1388 (953-1821)	0.201 (0.138-0.264)	2048 (1460-2858)	0.297 (0.212-0.415)	1.28 (0.91 to 1.64)
Oceania	1 (0-1)	0.022 (0.013-0.033)	1 (1-2)	0.021 (0.013-0.028)	-0.08 (-0.36 to 0.2)
Southeast Asia	124 (88-164)	0.052 (0.037-0.069)	469 (343-599)	0.126 (0.092-0.162)	2.92 (2.77 to 3.06)
Central Sub-Saharan Africa	8 (3-13)	0.034 (0.013-0.055)	29 (15-46)	0.044 (0.023-0.071)	1.56 (1.04 to 2.08)
Eastern Sub-Saharan Africa	35 (13-51)	0.042 (0.015-0.061)	114 (70-169)	0.055 (0.033-0.081)	0.59 (0.32 to 0.85)
Southern Sub-Saharan Africa	32 (22-40)	0.122 (0.085-0.155)	62 (43-84)	0.143 (0.099-0.194)	0.32 (0.14 to 0.5)
Western Sub-Saharan Africa	18 (11-26)	0.021 (0.013-0.03)	65 (43-92)	0.028 (0.019-0.04)	1.11 (1.04 to 1.18)
South Asia	79 (40-119)	0.015 (0.008-0.022)	272 (181-385)	0.027 (0.018-0.038)	2.24 (2.07 to 2.41)
Andean Latin America	13 (9-18)	0.071 (0.047-0.099)	37 (25-54)	0.107 (0.072-0.153)	1.69 (1.52 to 1.87)
Caribbean	19 (15-24)	0.104 (0.08-0.132)	33 (24-43)	0.137 (0.101-0.18)	1.12 (1.04 to 1.21)
Central Latin America	58 (45-73)	0.071 (0.056-0.089)	179 (137-228)	0.134 (0.103-0.171)	1.95 (1.81 to 2.08)
Tropical Latin America	72 (56-91)	0.092 (0.071-0.116)	211 (165-265)	0.176 (0.137-0.222)	1.89 (1.57 to 2.21)
North Africa and Middle East	28 (20-40)	0.018 (0.012-0.025)	45 (33-60)	0.014 (0.01-0.018)	-1.06 (-1.29 to -0.84)
Central Asia	44 (33-58)	0.133 (0.1-0.173)	48 (37-62)	0.098 (0.075-0.126)	-1.03 (-1.21 to -0.85)
Central Europe	214 (171-265)	0.345 (0.276-0.427)	190 (149-237)	0.36 (0.283-0.449)	-0.21 (-0.4 to -0.01)
Eastern Europe	364 (291-453)	0.33 (0.264-0.411)	304 (239-377)	0.316 (0.249-0.392)	-0.7 (-1.1 to -0.3)
Australasia	42 (33-52)	0.387 (0.302-0.486)	41 (30-53)	0.284 (0.21-0.365)	-1.01 (-1.14 to -0.88)
High-income Asia Pacific	320 (246-397)	0.344 (0.265-0.427)	190 (140-243)	0.243 (0.179-0.31)	-1.29 (-1.42 to -1.17)
High-income North America	348 (279-438)	0.234 (0.187-0.294)	439 (342-546)	0.26 (0.202-0.324)	0.45 (0.36 to 0.55)
Southern Latin America	67 (52-84)	0.273 (0.21-0.343)	83 (63-109)	0.24 (0.181-0.313)	0.02 (-0.12 to 0.16)
Western Europe	674 (532-837)	0.348 (0.275-0.433)	417 (320-525)	0.221 (0.17-0.279)	-1.46 (-1.57 to -1.36)
Disability-adjusted life years					
Global	198998 (154056-245924)	7.342 (5.684-9.073)	265347 (204207-337507)	6.72 (5.172-8.548)	-0.35 (-0.46 to -0.24)
SDI regions					
High SDI	69301 (55012-85591)	15.038 (11.937-18.572)	60330 (47302-74895)	12.012 (9.418-14.912)	-0.83 (-0.9 to -0.75)
High-middle SDI	69768 (53889-85790)	12.361 (9.548-15.2)	80227 (60856-105144)	12.743 (9.666-16.701)	-0.13 (-0.25 to -0.01)
Middle SDI	50745 (36055-65257)	5.573 (3.959-7.166)	95521 (73292-123861)	7.611 (5.84-9.869)	1.11 (0.89 to 1.33)
Low-middle SDI	6234 (4269-8568)	1.131 (0.775-1.555)	20787 (15327-27535)	2.046 (1.508-2.709)	2.25 (2.14 to 2.37)
Low SDI	2707 (1220-3858)	1.224 (0.552-1.745)	8229 (5374-11665)	1.517 (0.991-2.151)	0.57 (0.29 to 0.85)
Continents					
Africa	4944 (2797-6649)	1.729 (0.978-2.325)	14058 (9433-20112)	2.081 (1.396-2.977)	0.53 (0.4 to 0.65)
America	29349 (23377-36752)	7.976 (6.353-9.988)	49653 (39740-62277)	9.682 (7.749-12.143)	0.69 (0.63 to 0.76)
Asia	100995 (73107-127612)	6.111 (4.424-7.722)	154709 (117166-206885)	6.526 (4.942-8.727)	0.24 (-0.02 to 0.5)
Europe	63270 (50519-78173)	15.845 (12.652-19.577)	46482 (36646-57727)	12.086 (9.529-15.01)	-1.11 (-1.28 to -0.94)
WHO regions					
African Region	4746 (2584-6411)	2.083 (1.134-2.814)	13768 (9176-19733)	2.46 (1.64-3.526)	0.47 (0.34 to 0.6)
Eastern Mediterranean Region	483 (329-672)	0.28 (0.191-0.389)	1595 (1113-2246)	0.399 (0.278-0.562)	1.63 (1.38 to 1.87)
European Region	65050 (51914-80490)	15.272 (12.188-18.897)	48366 (38157-60077)	11.314 (8.926-14.053)	-1.21 (-1.38 to -1.04)
Region of the Americas	29349 (23377-36752)	7.976 (6.353-9.988)	49653 (39740-62277)	9.682 (7.749-12.143)	0.69 (0.63 to 0.76)
South-East Asia Region	7483 (4763-10550)	1.157 (0.737-1.632)	22529 (16482-29743)	2.002 (1.465-2.643)	1.77 (1.68 to 1.86)
Western Pacific Region	89838 (64505-114064)	10.523 (7.556-13.361)	127409 (94502-172087)	14.045 (10.417-18.97)	0.93 (0.6 to 1.26)
Global burden of disease regions					
East Asia	70646 (48414-92848)	10.256 (7.028-13.479)	103397 (73986-143957)	15.018 (10.746-20.909)	1.21 (0.85 to 1.58)
Oceania	38 (22-57)	1.194 (0.678-1.777)	77 (49-106)	1.082 (0.692-1.504)	-0.13 (-0.42 to 0.15)
Southeast Asia	6440 (4581-8486)	2.722 (1.936-3.587)	23508 (17169-29893)	6.34 (4.63-8.062)	2.75 (2.61 to 2.89)
Central Sub-Saharan Africa	414 (155-667)	1.696 (0.636-2.733)	1455 (749-2351)	2.231 (1.148-3.605)	1.55 (1.03 to 2.07)
Eastern Sub-Saharan Africa	1777 (619-2557)	2.13 (0.742-3.065)	5820 (3608-8614)	2.78 (1.723-4.114)	0.62 (0.35 to 0.88)
Southern Sub-Saharan Africa	1616 (1142-2045)	6.276 (4.435-7.939)	3110 (2171-4231)	7.204 (5.029-9.801)	0.27 (0.07 to 0.47)
Western Sub-Saharan Africa	902 (552-1274)	1.053 (0.644-1.488)	3268 (2150-4640)	1.425 (0.938-2.023)	1.15 (1.08 to 1.22)
South Asia	3937 (2027-5917)	0.744 (0.383-1.118)	13470 (9036-19024)	1.338 (0.898-1.89)	2.2 (2.04 to 2.36)
Andean Latin America	684 (450-960)	3.668 (2.417-5.152)	1918 (1296-2730)	5.485 (3.705-7.804)	1.67 (1.49 to 1.84)
Caribbean	988 (754-1249)	5.407 (4.129-6.838)	1675 (1241-2206)	6.994 (5.184-9.212)	1.05 (0.96 to 1.14)
Central Latin America	3079 (2411-3868)	3.773 (2.953-4.739)	9220 (7095-11691)	6.926 (5.33-8.782)	1.87 (1.73 to 2.01)
Tropical Latin America	3743 (2928-4730)	4.767 (3.729-6.024)	10602 (8310-13320)	8.847 (6.935-11.116)	1.79 (1.49 to 2.1)
North Africa and Middle East	1461 (1018-2064)	0.912 (0.635-1.288)	2300 (1670-3063)	0.688 (0.499-0.916)	-1.1 (-1.33 to -0.88)
Central Asia	2327 (1760-3014)	6.979 (5.278-9.04)	2437 (1866-3140)	4.998 (3.828-6.441)	-1.15 (-1.35 to -0.95)
Central Europe	10486 (8362-12961)	16.886 (13.466-20.872)	9168 (7213-11442)	17.401 (13.689-21.715)	-0.2 (-0.37 to -0.03)
Eastern Europe	18107 (14418-22527)	16.418 (13.073-20.425)	14941 (11786-18559)	15.527 (12.248-19.287)	-0.71 (-1.07 to -0.34)
Australasia	2077 (1620-2608)	19.245 (15.011-24.168)	2097 (1572-2714)	14.519 (10.883-18.797)	-0.91 (-1.03 to -0.78)
High-income Asia Pacific	15885 (12249-19742)	17.113 (13.196-21.268)	9507 (6983-12127)	12.154 (8.927-15.503)	-1.25 (-1.36 to -1.14)
High-income North America	17769 (14198-22331)	11.923 (9.527-14.984)	22295 (17379-27869)	13.218 (10.304-16.523)	0.45 (0.37 to 0.53)
Southern Latin America	3323 (2571-4165)	13.57 (10.496-17.008)	4188 (3175-5456)	12.073 (9.153-15.728)	0.07 (-0.08 to 0.21)
Western Europe	33300 (26266-41446)	17.217 (13.58-21.428)	20895 (16186-26281)	11.084 (8.586-13.941)	-1.43 (-1.53 to -1.33)

SDI, socio-demographic index; WHO, World Health Organization; ASR, age-standardized rate; EAPC, estimated annual percentage change; CI, confidence interval.

**Table 2:** Global and regional trends in the burden of early-onset colorectal cancer attributable to tobacco: deaths and disability-adjusted life years (1990–2021).

Location me	1990		2021		EAPC (95% CI)
	Number	ASR	Number	ASR	
Deaths					
Global	3394 (2185-4597)	0.125 (0.081-0.17)	3800 (2369-5310)	0.096 (0.06-0.134)	-0.96 (-1.04 to -0.88)
SDI regions					
High SDI	987 (643-1331)	0.214 (0.139-0.289)	690 (436-942)	0.137 (0.087-0.187)	-1.62 (-1.75 to -1.5)
High-middle SDI	1137 (730-1556)	0.201 (0.129-0.276)	1272 (781-1786)	0.202 (0.124-0.284)	-0.25 (-0.36 to -0.13)
Middle SDI	1024 (656-1411)	0.112 (0.072-0.155)	1415 (871-2012)	0.113 (0.069-0.16)	0.06 (-0.05 to 0.16)
Low-middle SDI	200 (123-283)	0.036 (0.022-0.051)	344 (213-498)	0.034 (0.021-0.049)	-0.25 (-0.34 to -0.17)
Low SDI	42 (25-60)	0.019 (0.011-0.027)	76 (47-108)	0.014 (0.009-0.02)	-1.12 (-1.2 to -1.04)
Continents					
Africa	72 (46-100)	0.025 (0.016-0.035)	150 (91-214)	0.022 (0.013-0.032)	-0.48 (-0.53 to -0.44)
America	454 (289-619)	0.123 (0.079-0.168)	473 (294-665)	0.092 (0.057-0.13)	-1.17 (-1.37 to -0.97)
Asia	1942 (1239-2670)	0.118 (0.075-0.162)	2530 (1555-3613)	0.107 (0.066-0.152)	-0.34 (-0.43 to -0.24)
Europe	919 (595-1236)	0.23 (0.149-0.309)	642 (408-870)	0.167 (0.106-0.226)	-1.29 (-1.46 to -1.12)
WHO regions					
African Region	47 (30-65)	0.021 (0.013-0.029)	90 (56-129)	0.016 (0.01-0.023)	-0.87 (-0.95 to -0.79)
Eastern Mediterranean Region	65 (41-92)	0.038 (0.024-0.053)	174 (107-243)	0.043 (0.027-0.061)	0.48 (0.45 to 0.51)
European Region	942 (610-1268)	0.221 (0.143-0.298)	667 (425-906)	0.156 (0.099-0.212)	-1.38 (-1.55 to -1.21)
Region of the Americas	454 (289-619)	0.123 (0.079-0.168)	473 (294-665)	0.092 (0.057-0.13)	-1.17 (-1.37 to -0.97)
South-East Asia Region	256 (158-364)	0.04 (0.024-0.056)	438 (261-646)	0.039 (0.023-0.057)	-0.05 (-0.18 to 0.09)
Western Pacific Region	1602 (1022-2222)	0.188 (0.12-0.26)	1923 (1182-2838)	0.212 (0.13-0.313)	0.33 (0.18 to 0.48)
Global burden of disease regions					
East Asia	1327 (837-1865)	0.193 (0.122-0.271)	1667 (1010-2490)	0.242 (0.147-0.362)	0.67 (0.51 to 0.84)
Oceania	1 (1-2)	0.041 (0.022-0.061)	3 (2-4)	0.038 (0.022-0.054)	-0.36 (-0.47 to -0.26)
Southeast Asia	192 (120-268)	0.081 (0.051-0.113)	447 (282-663)	0.121 (0.076-0.179)	1.24 (1.07 to 1.42)
Central Sub-Saharan Africa	4 (2-6)	0.016 (0.009-0.026)	11 (6-17)	0.016 (0.009-0.026)	0.27 (0.03 to 0.52)
Eastern Sub-Saharan Africa	17 (10-24)	0.02 (0.013-0.029)	36 (22-53)	0.017 (0.01-0.025)	-0.75 (-0.85 to -0.64)
Southern Sub-Saharan Africa	19 (12-26)	0.073 (0.045-0.101)	26 (17-38)	0.061 (0.039-0.088)	-0.56 (-0.73 to -0.4)
Western Sub-Saharan Africa	6 (3-9)	0.007 (0.004-0.01)	13 (8-20)	0.006 (0.003-0.009)	-0.5 (-0.54 to -0.45)
South Asia	137 (85-196)	0.026 (0.016-0.037)	181 (109-268)	0.018 (0.011-0.027)	-1.24 (-1.35 to -1.13)
Andean Latin America	4 (2-6)	0.021 (0.013-0.031)	10 (6-15)	0.027 (0.016-0.042)	0.88 (0.71 to 1.06)
Caribbean	14 (9-19)	0.076 (0.047-0.106)	16 (10-23)	0.068 (0.043-0.097)	-0.34 (-0.49 to -0.2)
Central Latin America	30 (19-41)	0.036 (0.023-0.05)	59 (37-82)	0.044 (0.028-0.062)	0.42 (0.32 to 0.53)
Tropical Latin America	78 (50-109)	0.1 (0.064-0.139)	97 (59-141)	0.081 (0.049-0.118)	-1.42 (-1.78 to -1.06)
North Africa and Middle East	117 (72-166)	0.073 (0.045-0.103)	216 (131-300)	0.065 (0.039-0.09)	-0.49 (-0.72 to -0.25)
Central Asia	31 (20-43)	0.094 (0.061-0.128)	34 (22-47)	0.069 (0.045-0.096)	-0.99 (-1.12 to -0.87)
Central Europe	184 (119-248)	0.296 (0.191-0.4)	125 (79-171)	0.237 (0.15-0.325)	-1.13 (-1.35 to -0.91)
Eastern Europe	237 (153-318)	0.215 (0.139-0.288)	213 (134-287)	0.221 (0.139-0.299)	-0.53 (-0.84 to -0.22)
Australasia	23 (15-32)	0.214 (0.136-0.292)	18 (11-26)	0.125 (0.075-0.183)	-1.82 (-1.93 to -1.72)
High-income Asia Pacific	212 (139-285)	0.228 (0.149-0.307)	102 (64-139)	0.13 (0.082-0.178)	-2.04 (-2.17 to -1.92)
High-income North America	287 (183-388)	0.193 (0.123-0.261)	241 (147-335)	0.143 (0.087-0.199)	-1.07 (-1.29 to -0.84)
Southern Latin America	44 (27-61)	0.179 (0.111-0.248)	53 (34-75)	0.152 (0.097-0.215)	-0.45 (-0.6 to -0.3)
Western Europe	431 (273-585)	0.223 (0.141-0.303)	234 (144-325)	0.124 (0.077-0.172)	-1.76 (-1.96 to -1.56)
Disability-adjusted life years					
Global	164341 (105596-222764)	6.063 (3.896-8.219)	184199 (114491-257059)	4.665 (2.9-6.51)	-0.97 (-1.05 to -0.9)
SDI regions					
High SDI	47826 (30930-64677)	10.378 (6.711-14.034)	33745 (21304-45807)	6.719 (4.242-9.121)	-1.59 (-1.71 to -1.48)
High-middle SDI	55079 (35381-75558)	9.759 (6.269-13.387)	61958 (38137-87229)	9.841 (6.058-13.855)	-0.25 (-0.36 to -0.13)
Middle SDI	49749 (31841-68804)	5.463 (3.497-7.556)	68387 (41993-96898)	5.449 (3.346-7.721)	0 (-0.08 to 0.09)
Low-middle SDI	9533 (5872-13521)	1.73 (1.065-2.454)	16349 (10128-23615)	1.609 (0.997-2.324)	-0.27 (-0.36 to -0.19)
Low SDI	1952 (1184-2801)	0.883 (0.536-1.267)	3590 (2207-5105)	0.662 (0.407-0.941)	-1.1 (-1.17 to -1.02)
Continents					
Africa	3479 (2237-4821)	1.216 (0.782-1.686)	7173 (4383-10238)	1.062 (0.649-1.515)	-0.49 (-0.54 to -0.44)
America	22106 (14130-30106)	6.007 (3.84-8.182)	22949 (14230-32056)	4.475 (2.775-6.25)	-1.19 (-1.38 to -1)
Asia	94223 (59903-129422)	5.701 (3.625-7.831)	122884 (75218-175612)	5.183 (3.173-7.407)	-0.36 (-0.44 to -0.28)
Europe	44233 (28524-59500)	11.078 (7.144-14.901)	30936 (19709-41971)	8.044 (5.125-10.913)	-1.28 (-1.44 to -1.12)
WHO regions					
African Region	2256 (1442-3123)	0.99 (0.633-1.371)	4283 (2643-6120)	0.765 (0.472-1.094)	-0.89 (-0.97 to -0.81)
Eastern Mediterranean Region	3131 (1975-4421)	1.815 (1.144-2.562)	8396 (5159-11802)	2.099 (1.29-2.95)	0.49 (0.46 to 0.53)
European Region	45375 (29262-61058)	10.653 (6.87-14.334)	32190 (20505-43778)	7.53 (4.797-10.24)	-1.37 (-1.53 to -1.21)
Region of the Americas	22106 (14130-30106)	6.007 (3.84-8.182)	22949 (14230-32056)	4.475 (2.775-6.25)	-1.19 (-1.38 to -1)
South-East Asia Region	12155 (7468-17295)	1.88 (1.155-2.675)	20756 (12348-30683)	1.845 (1.097-2.727)	-0.08 (-0.22 to 0.07)
Western Pacific Region	77944 (49634-108050)	9.13 (5.814-12.657)	93967 (57447-138705)	10.358 (6.333-15.29)	0.3 (0.17 to 0.44)
Global burden of disease regions					
East Asia	64670 (40744-90818)	9.388 (5.915-13.184)	81640 (49298-122657)	11.858 (7.16-17.815)	0.65 (0.49 to 0.8)
Oceania	63 (34-93)	1.967 (1.06-2.925)	129 (76-184)	1.823 (1.072-2.607)	-0.36 (-0.47 to -0.24)
Southeast Asia	9278 (5757-12899)	3.921 (2.433-5.452)	21317 (13380-31544)	5.749 (3.608-8.507)	1.18 (1 to 1.35)
Central Sub-Saharan Africa	186 (107-298)	0.762 (0.437-1.219)	504 (275-794)	0.773 (0.422-1.218)	0.27 (0.02 to 0.52)
Eastern Sub-Saharan Africa	792 (489-1134)	0.95 (0.586-1.359)	1677 (1023-2494)	0.801 (0.489-1.191)	-0.73 (-0.83 to -0.63)
Southern Sub-Saharan Africa	911 (568-1266)	3.538 (2.207-4.914)	1263 (795-1808)	2.926 (1.842-4.188)	-0.63 (-0.81 to -0.45)
Western Sub-Saharan Africa	275 (164-405)	0.321 (0.192-0.473)	637 (364-935)	0.278 (0.159-0.408)	-0.47 (-0.51 to -0.42)
South Asia	6441 (3990-9250)	1.217 (0.754-1.748)	8486 (5119-12570)	0.843 (0.509-1.249)	-1.26 (-1.37 to -1.14)
Andean Latin America	189 (116-273)	1.014 (0.625-1.464)	461 (282-701)	1.319 (0.805-2.003)	0.9 (0.72 to 1.07)
Caribbean	668 (414-927)	3.656 (2.266-5.076)	778 (491-1113)	3.248 (2.05-4.65)	-0.38 (-0.53 to -0.23)
Central Latin America	1432 (911-1989)	1.755 (1.116-2.437)	2824 (1772-3944)	2.121 (1.331-2.963)	0.39 (0.28 to 0.5)
Tropical Latin America	3782 (2423-5272)	4.817 (3.085-6.714)	4588 (2805-6681)	3.829 (2.341-5.575)	-1.5 (-1.85 to -1.15)
North Africa and Middle East	5698 (3494-8097)	3.555 (2.18-5.052)	10472 (6384-14504)	3.132 (1.909-4.338)	-0.49 (-0.74 to -0.25)
Central Asia	1537 (996-2091)	4.608 (2.986-6.269)	1638 (1055-2253)	3.359 (2.163-4.62)	-1.09 (-1.21 to -0.96)
Central Europe	8762 (5655-11853)	14.11 (9.107-19.088)	5898 (3724-8083)	11.193 (7.069-15.341)	-1.13 (-1.33 to -0.93)
Eastern Europe	11387 (7395-15286)	10.325 (6.705-13.86)	10230 (6457-13764)	10.632 (6.711-14.304)	-0.52 (-0.8 to -0.24)
Australasia	1112 (706-1524)	10.301 (6.54-14.121)	894 (535-1319)	6.189 (3.703-9.137)	-1.73 (-1.84 to -1.62)
High-income Asia Pacific	10216 (6720-13674)	11.006 (7.239-14.731)	4949 (3110-6826)	6.327 (3.976-8.727)	-1.98 (-2.1 to -1.87)
High-income North America	14082 (8989-19002)	9.449 (6.032-12.751)	11885 (7203-16508)	7.046 (4.27-9.788)	-1.06 (-1.27 to -0.86)
Southern Latin America	2077 (1282-2864)	8.481 (5.236-11.695)	2522 (1617-3552)	7.272 (4.661-10.24)	-0.4 (-0.55 to -0.26)
Western Europe	20783 (13168-28271)	10.745 (6.808-14.616)	11407 (7031-15806)	6.051 (3.73-8.385)	-1.74 (-1.94 to -1.54)

SDI, socio-demographic index; WHO, World Health Organization; ASR, age-standardized rate; EAPC, estimated annual percentage change; CI, confidence interval.

**Table 3:** Global and regional trends in the burden of early-onset colorectal cancer attributable to low physical activity: deaths and disability-adjusted life years (1990–2021).

Location name	1990		2021		EAPC (95% CI)
	Number	ASR	Number	ASR	
Deaths					
Global	1143 (705-1599)	0.042 (0.026-0.059)	1657 (1015-2297)	0.042 (0.026-0.058)	-0.08 (-0.31 to 0.16)
SDI regions					
High SDI	386 (237-541)	0.084 (0.051-0.117)	377 (221-524)	0.075 (0.044-0.104)	-0.39 (-0.61 to -0.16)
High-middle SDI	261 (151-386)	0.046 (0.027-0.068)	320 (183-492)	0.051 (0.029-0.078)	0.11 (-0.2 to 0.43)
Middle SDI	352 (211-533)	0.039 (0.023-0.058)	618 (373-896)	0.049 (0.03-0.071)	0.73 (0.46 to 1)
Low-middle SDI	114 (67-165)	0.021 (0.012-0.03)	267 (161-383)	0.026 (0.016-0.038)	0.82 (0.74 to 0.9)
Low SDI	29 (14-45)	0.013 (0.006-0.02)	74 (43-109)	0.014 (0.008-0.02)	0.2 (-0.06 to 0.46)
Continents					
Africa	81 (47-117)	0.028 (0.017-0.041)	198 (114-286)	0.029 (0.017-0.042)	0.22 (0.13 to 0.32)
America	117 (72-172)	0.032 (0.02-0.047)	278 (166-396)	0.054 (0.032-0.077)	1.9 (1.65 to 2.16)
Asia	692 (421-1017)	0.042 (0.025-0.062)	968 (570-1426)	0.041 (0.024-0.06)	-0.19 (-0.49 to 0.11)
Europe	250 (150-350)	0.063 (0.037-0.088)	211 (122-290)	0.055 (0.032-0.075)	-0.51 (-0.64 to -0.37)
WHO regions					
African Region	42 (25-59)	0.019 (0.011-0.026)	102 (60-146)	0.018 (0.011-0.026)	0.04 (-0.09 to 0.18)
Eastern Mediterranean Region	83 (47-123)	0.048 (0.027-0.071)	237 (137-345)	0.059 (0.034-0.086)	0.85 (0.71 to 0.98)
European Region	255 (153-357)	0.06 (0.036-0.084)	217 (126-298)	0.051 (0.03-0.07)	-0.61 (-0.74 to -0.48)
Region of the Americas	117 (72-172)	0.032 (0.02-0.047)	278 (166-396)	0.054 (0.032-0.077)	1.9 (1.65 to 2.16)
South-East Asia Region	140 (78-212)	0.022 (0.012-0.033)	275 (157-418)	0.024 (0.014-0.037)	0.29 (0.19 to 0.39)
Western Pacific Region	487 (287-752)	0.057 (0.034-0.088)	519 (292-847)	0.057 (0.032-0.093)	-0.2 (-0.65 to 0.26)
Global burden of disease regions					
East Asia	324 (171-567)	0.047 (0.025-0.082)	379 (188-698)	0.055 (0.027-0.101)	0.22 (-0.26 to 0.7)
Oceania	1 (1-2)	0.045 (0.026-0.07)	3 (2-5)	0.043 (0.025-0.072)	-0.18 (-0.25 to -0.12)
Southeast Asia	74 (41-116)	0.031 (0.017-0.049)	188 (102-297)	0.051 (0.028-0.08)	1.79 (1.71 to 1.87)
Central Sub-Saharan Africa	3 (1-5)	0.011 (0.006-0.02)	8 (4-15)	0.012 (0.006-0.023)	0.31 (0.08 to 0.55)
Eastern Sub-Saharan Africa	10 (5-16)	0.012 (0.006-0.019)	24 (13-37)	0.012 (0.006-0.018)	-0.19 (-0.4 to 0.03)
Southern Sub-Saharan Africa	18 (10-27)	0.07 (0.04-0.105)	36 (21-54)	0.084 (0.048-0.126)	0.81 (0.53 to 1.09)
Western Sub-Saharan Africa	9 (4-14)	0.01 (0.005-0.016)	26 (14-42)	0.011 (0.006-0.018)	0.59 (0.41 to 0.77)
South Asia	83 (45-131)	0.016 (0.009-0.025)	157 (86-239)	0.016 (0.009-0.024)	-0.36 (-0.57 to -0.15)
Andean Latin America	3 (1-5)	0.016 (0.007-0.027)	8 (4-13)	0.021 (0.011-0.037)	1.04 (0.91 to 1.17)
Caribbean	8 (4-13)	0.044 (0.025-0.069)	14 (8-23)	0.06 (0.033-0.095)	1.28 (1.2 to 1.37)
Central Latin America	17 (10-24)	0.02 (0.012-0.03)	66 (38-99)	0.05 (0.029-0.074)	3.35 (2.87 to 3.84)
Tropical Latin America	29 (16-49)	0.038 (0.02-0.063)	103 (51-172)	0.086 (0.043-0.143)	2.74 (2.64 to 2.85)
North Africa and Middle East	99 (55-148)	0.062 (0.035-0.093)	243 (139-356)	0.073 (0.042-0.106)	0.72 (0.48 to 0.96)
Central Asia	5 (3-8)	0.016 (0.009-0.024)	6 (4-10)	0.013 (0.007-0.02)	-0.52 (-0.64 to -0.4)
Central Europe	34 (20-51)	0.055 (0.031-0.083)	32 (18-47)	0.06 (0.034-0.09)	-0.11 (-0.29 to 0.08)
Eastern Europe	37 (18-60)	0.033 (0.017-0.054)	32 (16-55)	0.033 (0.017-0.057)	-0.55 (-0.78 to -0.32)
Australasia	14 (8-22)	0.132 (0.072-0.203)	18 (10-29)	0.124 (0.068-0.198)	-0.06 (-0.26 to 0.14)
High-income Asia Pacific	155 (88-235)	0.167 (0.095-0.254)	103 (55-163)	0.131 (0.07-0.208)	-0.83 (-1.04 to -0.62)
High-income North America	54 (26-93)	0.036 (0.018-0.062)	78 (39-131)	0.046 (0.023-0.077)	0.93 (0.58 to 1.28)
Southern Latin America	7 (3-12)	0.029 (0.014-0.05)	11 (5-19)	0.03 (0.015-0.054)	0.14 (-0.13 to 0.41)
Western Europe	158 (93-225)	0.082 (0.048-0.116)	122 (71-166)	0.065 (0.038-0.088)	-0.65 (-0.78 to -0.53)
Disability-adjusted life years					
Global	55829 (34572-77995)	2.06 (1.275-2.878)	80825 (49388-111915)	2.047 (1.251-2.834)	-0.08 (-0.32 to 0.16)
SDI regions					
High SDI	18734 (11536-26053)	4.065 (2.503-5.653)	18547 (10951-25677)	3.693 (2.18-5.112)	-0.33 (-0.55 to -0.1)
High-middle SDI	12770 (7454-18993)	2.262 (1.321-3.365)	15652 (9126-24059)	2.486 (1.45-3.821)	0.11 (-0.22 to 0.45)
Middle SDI	17358 (10329-26228)	1.906 (1.134-2.88)	30063 (18125-43849)	2.395 (1.444-3.494)	0.66 (0.39 to 0.93)
Low-middle SDI	5518 (3232-7926)	1.001 (0.587-1.438)	12906 (7784-18640)	1.27 (0.766-1.834)	0.83 (0.74 to 0.91)
Low SDI	1391 (653-2153)	0.629 (0.295-0.974)	3575 (2064-5218)	0.659 (0.381-0.962)	0.24 (0 to 0.48)
Continents					
Africa	4009 (2327-5725)	1.402 (0.814-2.002)	9701 (5633-14090)	1.436 (0.834-2.085)	0.22 (0.12 to 0.32)
America	5731 (3522-8337)	1.557 (0.957-2.266)	13492 (8099-19185)	2.631 (1.579-3.741)	1.88 (1.61 to 2.15)
Asia	33953 (20724-49525)	2.054 (1.254-2.997)	47242 (28056-69375)	1.993 (1.183-2.926)	-0.21 (-0.51 to 0.09)
Europe	12032 (7186-16754)	3.013 (1.8-4.196)	10260 (5932-14140)	2.668 (1.542-3.677)	-0.46 (-0.6 to -0.32)
WHO regions					
African Region	2078 (1203-2890)	0.912 (0.528-1.268)	4925 (2919-7061)	0.88 (0.522-1.262)	0.01 (-0.14 to 0.15)
Eastern Mediterranean Region	4065 (2285-6009)	2.355 (1.324-3.482)	11663 (6807-16894)	2.915 (1.701-4.223)	0.86 (0.72 to 0.99)
European Region	12263 (7344-17092)	2.879 (1.724-4.013)	10569 (6127-14531)	2.472 (1.433-3.399)	-0.56 (-0.7 to -0.43)
Region of the Americas	5731 (3522-8337)	1.557 (0.957-2.266)	13492 (8099-19185)	2.631 (1.579-3.741)	1.88 (1.61 to 2.15)
South-East Asia Region	6668 (3745-9996)	1.031 (0.579-1.546)	13026 (7584-19626)	1.158 (0.674-1.744)	0.26 (0.16 to 0.36)
Western Pacific Region	24075 (14270-36821)	2.82 (1.672-4.313)	25681 (14691-42152)	2.831 (1.619-4.647)	-0.2 (-0.66 to 0.25)
Global burden of disease regions					
East Asia	16180 (8569-28062)	2.349 (1.244-4.074)	18829 (9526-34071)	2.735 (1.384-4.949)	0.18 (-0.31 to 0.67)
Oceania	71 (41-110)	2.208 (1.29-3.435)	151 (85-247)	2.133 (1.194-3.498)	-0.22 (-0.29 to -0.15)
Southeast Asia	3547 (1973-5557)	1.499 (0.834-2.349)	8925 (4869-14067)	2.407 (1.313-3.794)	1.74 (1.66 to 1.83)
Central Sub-Saharan Africa	135 (67-238)	0.554 (0.275-0.976)	388 (192-717)	0.595 (0.295-1.099)	0.3 (0.06 to 0.55)
Eastern Sub-Saharan Africa	475 (245-759)	0.569 (0.293-0.91)	1179 (632-1785)	0.563 (0.302-0.852)	-0.15 (-0.37 to 0.06)
Southern Sub-Saharan Africa	904 (515-1329)	3.511 (2.001-5.158)	1762 (1011-2651)	4.082 (2.341-6.14)	0.72 (0.41 to 1.03)
Western Sub-Saharan Africa	421 (219-669)	0.492 (0.256-0.782)	1250 (679-2002)	0.545 (0.296-0.873)	0.58 (0.41 to 0.76)
South Asia	3941 (2218-6107)	0.745 (0.419-1.154)	7485 (4144-11331)	0.744 (0.412-1.126)	-0.36 (-0.56 to -0.16)
Andean Latin America	141 (68-241)	0.755 (0.366-1.293)	364 (193-625)	1.04 (0.553-1.786)	1.03 (0.91 to 1.16)
Caribbean	390 (220-604)	2.134 (1.206-3.308)	702 (384-1093)	2.933 (1.606-4.563)	1.24 (1.15 to 1.33)
Central Latin America	829 (497-1217)	1.015 (0.609-1.491)	3242 (1894-4815)	2.435 (1.423-3.617)	3.31 (2.82 to 3.81)
Tropical Latin America	1440 (776-2384)	1.834 (0.988-3.036)	4939 (2549-8182)	4.122 (2.127-6.828)	2.69 (2.57 to 2.81)
North Africa and Middle East	4898 (2733-7310)	3.056 (1.705-4.561)	12038 (6927-17488)	3.601 (2.072-5.231)	0.74 (0.51 to 0.97)
Central Asia	261 (150-391)	0.784 (0.45-1.172)	312 (175-467)	0.641 (0.358-0.959)	-0.6 (-0.73 to -0.47)
Central Europe	1641 (931-2454)	2.642 (1.5-3.953)	1494 (845-2230)	2.836 (1.604-4.232)	-0.09 (-0.26 to 0.08)
Eastern Europe	1750 (891-2849)	1.587 (0.808-2.583)	1533 (757-2573)	1.594 (0.787-2.674)	-0.55 (-0.76 to -0.33)
Australasia	690 (383-1062)	6.399 (3.55-9.846)	901 (496-1414)	6.24 (3.435-9.79)	0.08 (-0.12 to 0.28)
High-income Asia Pacific	7534 (4296-11370)	8.116 (4.628-12.249)	5033 (2719-7968)	6.435 (3.476-10.186)	-0.76 (-0.95 to -0.56)
High-income North America	2650 (1310-4466)	1.778 (0.879-2.997)	3805 (1957-6249)	2.256 (1.161-3.705)	0.93 (0.56 to 1.3)
Southern Latin America	337 (163-587)	1.376 (0.666-2.396)	506 (244-903)	1.458 (0.702-2.604)	0.16 (-0.12 to 0.44)
Western Europe	7593 (4513-10664)	3.926 (2.333-5.513)	5986 (3469-8216)	3.175 (1.84-4.358)	-0.59 (-0.71 to -0.46)

SDI, socio-demographic index; WHO, World Health Organization; ASR, age-standardized rate; EAPC, estimated annual percentage change; CI, confidence interval.

### 3.2 Regional trends of early-onset CRC attributable to three behavioral risk factors

Furthermore, the burden of early-onset CRC attributable to three behavioral risk factors displayed obvious regional differences and was closely linked with SDI levels. From 1990 to 2021, the largest increases in early-onset CRC-related deaths and DALYs attributable to high alcohol use were observed in the low-middle SDI regions (**Table 1**). For early-onset CRC related to tobacco, the burdens of deaths and DALYs declined in most SDI regions, except for middle SDI regions. The most pronounced reductions in ASMR and age-standardized DALYs rate were found in the high SDI regions (**Table 2**). Similarly, for early-onset CRC attributable to low physical activity, the greatest increases in deaths and DALYs were also observed in the low-middle SDI regions (**Table 3**). Notably, Europe experienced the greatest reductions in early-onset CRC-related deaths and DALYs attributable to three behavioral risk factors (**Tables 1**–**3**). From 1990 to 2021, Southeast Asia represented the most increases in the burdens of early-onset CRC-related deaths and DALYs attributable to high alcohol use and tobacco (**Tables 1**, **2**). For low physical activity, the largest increase in early-onset CRC-related deaths and DALYs was observed in Central Latin America (**Table 3**).

### 3.3 National trends of early-onset CRC attributable to three behavioral risk factors

In 2021, the highest burdens of early-onset CRC-related deaths and DALYs attributable to high alcohol use were observed in Bulgaria. The most substantial reductions in ASMR and age-standardized DALYs rate were identified in Sudan (**Supplementary Figures 1A, B** and **Supplementary Table 1**). For early-onset CRC attributable to tobacco, Lesotho exhibited the highest increases in both ASMR and age-standardized DALYs rate (**Supplementary Figures 1C, D** and **Supplementary Table 2**). Regarding early-onset CRC attributable to low physical activity, Viet Nam had the largest increases in ASMR and age-standardized DALYs rate from 1990 to 2021 (**Supplementary Figures 1E, F** and **Supplementary Table 3**).

### 3.4 Sex and age patterns in different regions

**Figure 1:**
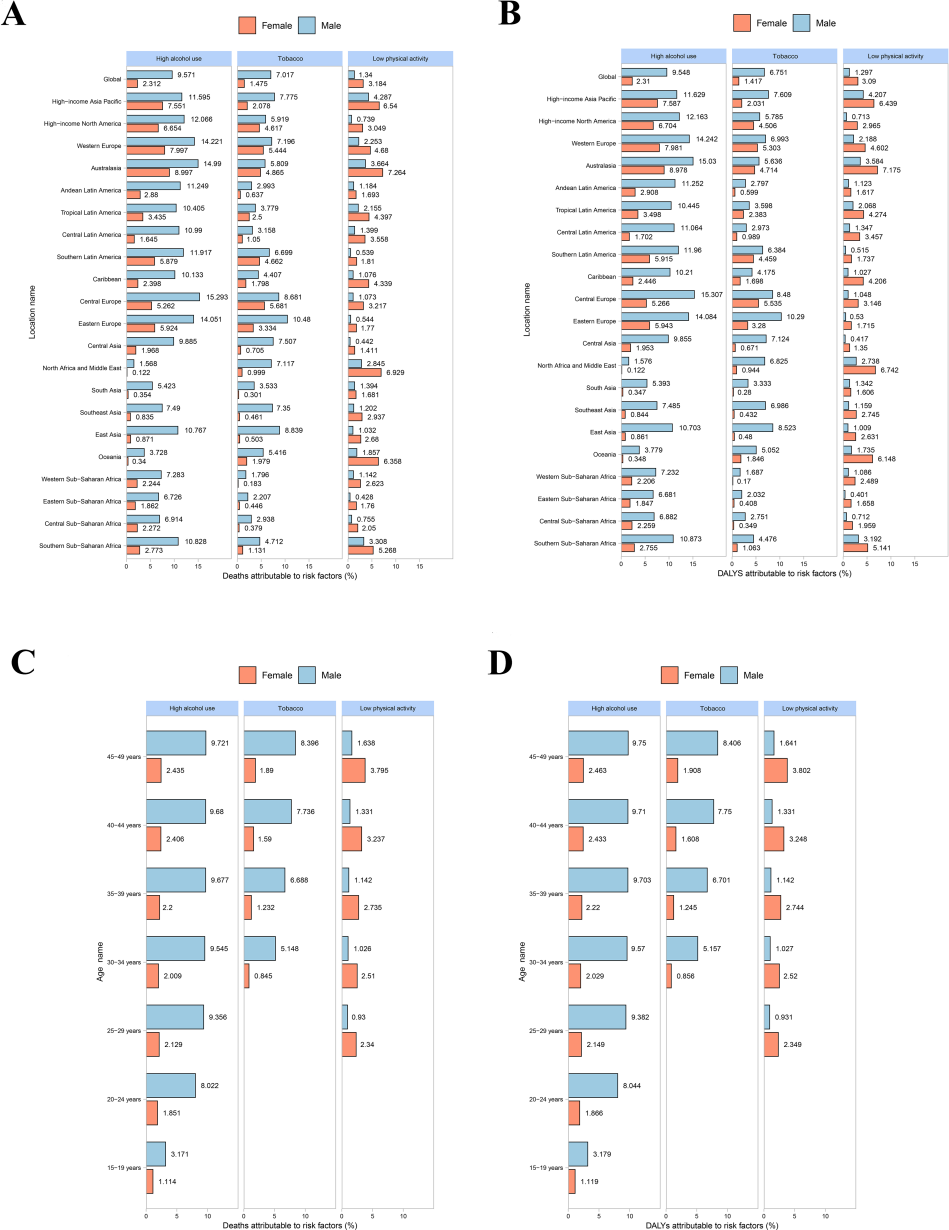
Fraction of early-onset CRC attributable to three behavioral risk factors by GBD regions, age and sex in 2021. **(A)** Deaths-GBD regions. **(B)** DALYs-GBD regions. **(C)** Deaths-age. **(D)** DALYs-age. CRC, colorectal cancer; DALYs, disability-adjusted life years; GBD, global burden of disease.

Globally, the proportions of early-onset CRC-related deaths and DALYs attributable to high alcohol use and tobacco were higher in males than females. In contrast, the proportions attributable to low physical activity were higher in females than in males (**Figures 1A, B**). Going further, our data indicated that the proportions of early-onset CRC-related deaths and DALYs attributable to high alcohol use and tobacco were higher in males than females across all age subgroups. Conversely, the proportions of early-onset CRC-related deaths and DALYs attributable to low physical activity were higher in females than males across all age subgroups (**Figures 1C, D**). In both sexes, early-onset CRC-related deaths and DALYs attributable to three behavioral risk factors increased with age, with highest proportions were observed in the 45–49 years age group across males and females (**Figures 1C, D**).

In the high SDI regions, the proportions of early-onset CRC-related deaths and DALYs attributable to three behavioral risk factors were highest in both sexes in 1990 and remained high in 2021 (**Supplementary Figures 2A, B**). On the other hand, in the middle SDI regions, the peaks of early-onset CRC-related deaths and DALYs attributable to three behavioral risk factors occurred in the 45–49 years age group across males and females (**Supplementary Figures 3A, B**). Among individuals aged 45–49 years, the number of early-onset CRC-related deaths and DALYs attributable to high alcohol use and tobacco was higher in males, while those attributable to low physical activity were higher in females (**Supplementary Figure 3A, B**).

### 3.5 The trends in age-specific rates and ASRs of early-onset CRC attributable to three behavioral risk factors

**Figure 2:**
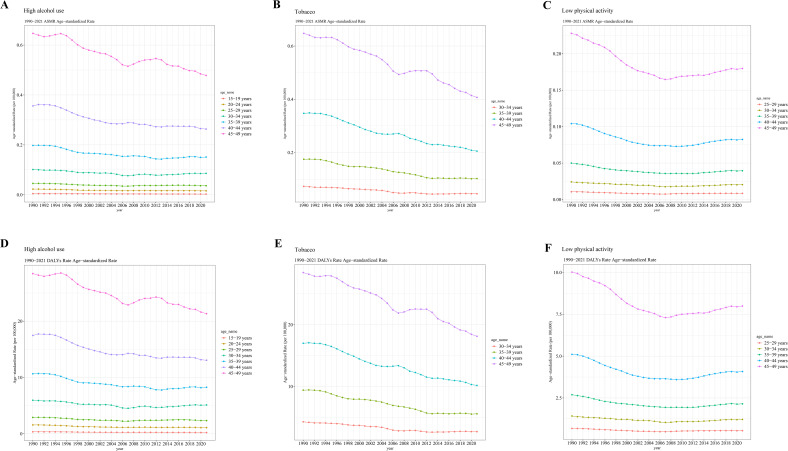
The global trends in age-specific rates of early-onset CRC attributable to three behavioral risk factors from 1990 to 2021. **(A)** ASMR caused by high alcohol use. **(B)** ASMR caused by tobacco. **(C)** ASMR caused by low physical activity. **(D)** Age-standardized DALYs rate caused by high alcohol use. **(E)** Age-standardized DALYs rate caused by tobacco. **(F)** Age-standardized DALYs rate caused by low physical activity. CRC, colorectal cancer; ASMR, age-standardized mortality rate; DALY, disability-adjusted life year.

**Figure 3:**
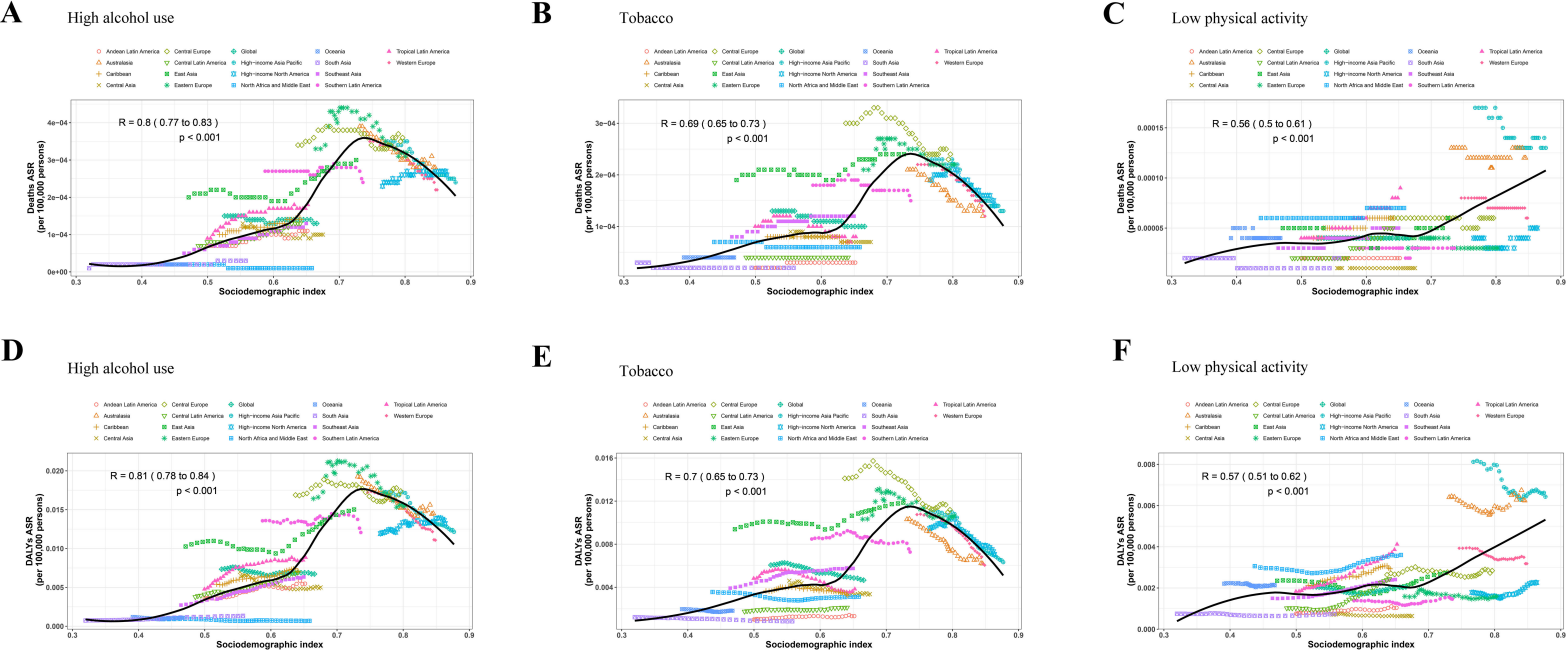
The trend in ASRs of early-onset CRC attributable to three behavioral risk factors across GBD regions by SDI from 1990 to 2021. **(A)** ASMR caused by high alcohol use. **(B)** ASMR caused by tobacco. **(C)** ASMR caused by low physical activity. **(D)** Age-standardized DALYs rate caused by high alcohol use. **(E)** Age-standardized DALYs rate caused by tobacco. **(F)** Age-standardized DALYs rate caused by low physical activity. CRC, colorectal cancer; ASMR, age-standardized mortality rate; GBD, global burden of disease; SDI, socio-demographic index; DALYs, disability-adjusted life years.

Overall, the ASMR and age-standardized DALYs rate of early-onset CRC attributable to high alcohol use, tobacco and low physical activity increased with age (**Figure 2**). Over the study period, the greatest reductions in ASMR and age-standardized DALYs rate were observed in the 45–49 years age group (**Figure 2**). After 2012, early-onset CRC-related death and DALYs caused by high alcohol use and tobacco showed a continuous downward trend in the 45–49 age group (**Figures 2A, B**, **2D, E**). In addition, since 2007, early-onset CRC-related death and DALYs caused by low physical activity have exhibited a continuous upward trend in the 45–49 age group (**Figures 2C, F**). On the other hand, our data displayed a significant positive correlation between SDI and both the ASMR and age-standardized DALYs rate of early-onset CRC attributable to three behavioral risk factors across different GBD regions from 1990 to 2021 (**Figure 3** and **Table 4**). These findings suggested that the mortality and DALYs attributable to three behavioral risk factors were higher in GBD areas with higher SDI levels.

**Table 4:** The trend in ASRs of early-onset CRC attributable to three behavioral risk factors across GBD regions by SDI.

Indicator	Behavioral risk factor	R	*P*
ASMR	High alcohol use	0.80 (0.77 to 0.83)	<0.001
	Tobacco	0.69 (0.65 to 0.73)	<0.001
	Low physical activity	0.56 (0.50 to 0.61)	<0.001
Age-standardized DALYs rate	High alcohol use	0.81 (0.78 to 0.84)	<0.001
	Tobacco	0.70 (0.65 to 0.73)	<0.001
	Low physical activity	0.57 (0.51 to 0.62)	<0.001

CRC, colorectal cancer; ASMR, age-standardized mortality rate; DALYs, disability-adjusted life years.

### 3.6 Future forecast

**Figure 4:**
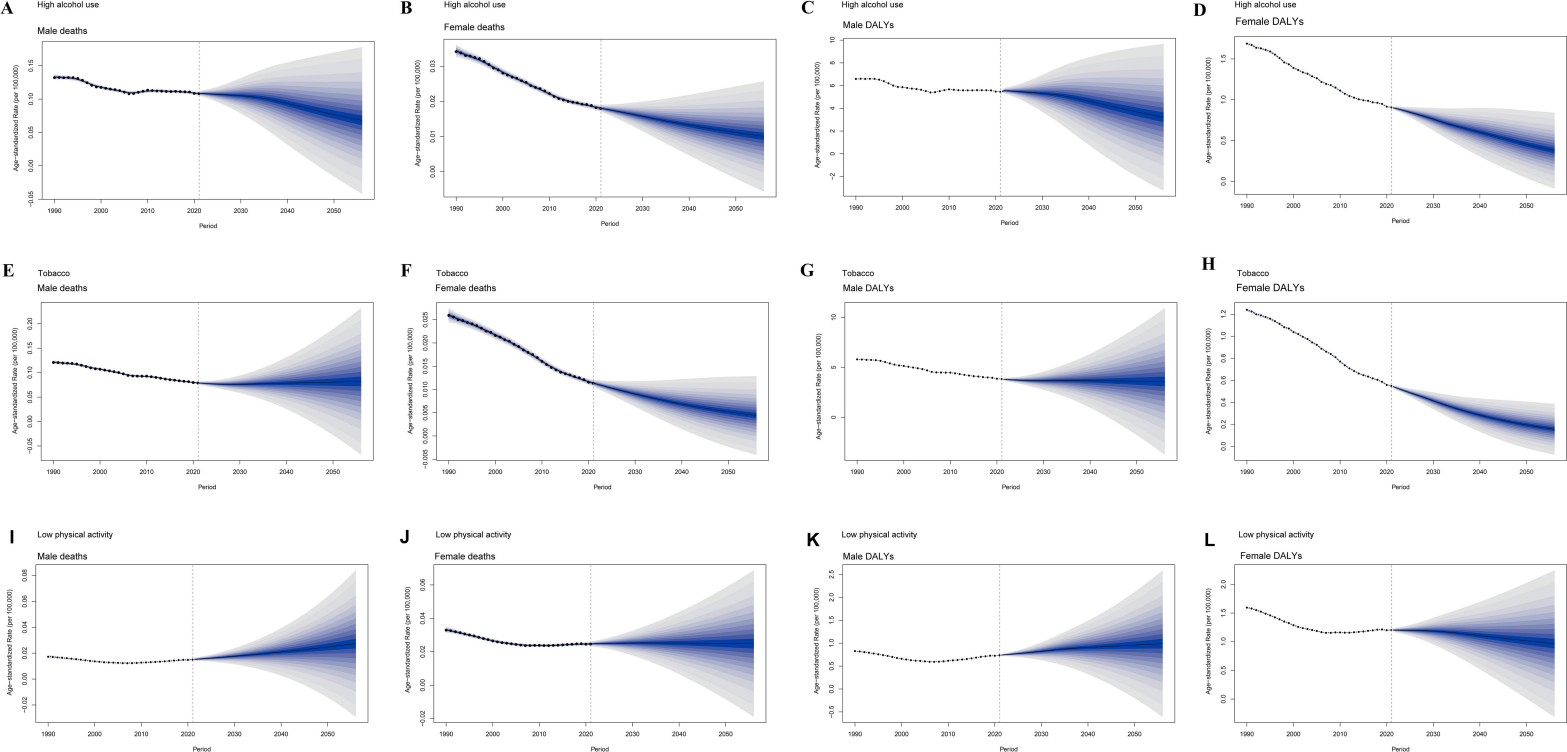
Future forecast of global burden of early-onset CRC attributable to three behavioral risk factors by 2050. **(A)** Male deaths caused by high alcohol use. **(B)** Female deaths caused by high alcohol use. **(C)** Male DALYs caused by high alcohol use. **(D)** Female DALYs caused by high alcohol use. **(E)** Male deaths caused by tobacco. **(F)** Female deaths caused by tobacco. **(G)** Male DALYs caused by tobacco. **(H)** Female DALYs caused by tobacco. **(I)** Male deaths caused by low physical activity. **(J)** Female deaths caused by low physical activity. **(K)** Male DALYs caused by low physical activity. **(L)** Female DALYs caused by low physical activity. Shaded areas represent 95% uncertainty intervals. CRC, colorectal cancer; DALYs, disability-adjusted life years.

The global burden of early-onset CRC attributable to three behavioral risk factors was predicted from 2021 to 2050 for both sexes (**Figure 4**). Our results revealed that the global ASMR and age-standardized DALYs rate of early-onset CRC attributable to high alcohol use were predicted to decline by 2050 in both males and females, with a greater reduction observed in males (**Figures 4A–D**). As for early-onset CRC attributable to tobacco, the age-standardized DALYs rate was decreased in both sexes (**Figures 4G, H**). Yet, the ASMR of early-onset CRC attributable to tobacco in both sexes remain relatively stable in both males and females (**Figures 4E, F**). Regarding low physical activity, the burden of early-onset CRC in both sexes displayed a relatively stable trend in the next 30 years (**Figures 4I–L**).

## 4 Discussion

Our results indicated that the burden of early-onset CRC attributable to three behavioral risk factors (high alcohol use, tobacco and low physical activity) was reduced from 1990 to 2021. In recent years, a global increase in the incidence of early-onset CRC has become one of the major public health challenges (19–21). The total increase of early-onset CRC incidence may be related to the factors of diet and metabolism, which are the driving factors for early-onset CRC (22, 23). As previous reported, GBD studies have revealed differences in the mortality and DALYs of early-onset CRC across regions and countries, aiding in the identification of high-risk populations and areas (24–26).

GBD 2019 study has reported that the increase in the incidence of early-onset CRC in 2019 is closely related to alcohol consumption (25). According to the latest GBD 2021 data, the global prevalence and incidence of early-onset CRC continue to rise, while mortality and DALYs have shown a declining trend (26). Additionally, in 2021, middle- and high-SDI regions, as well as East Asia bore the greatest burden of early-onset CRC (26). The high incidence of CRC in younger populations is related to several risk factors. In addition to known dietary risk factors, recent studies have shown that some behavioral risk factors are strongly associated with the progression of early-onset CRC, including alcohol consumption, tobacco, and low physical activity (12, 27). In Saudi Arabia, Kuwait, and Qatar, the attributable proportion of early-onset CRC in females due to low physical activity has remained high (12). Going further, our findings firstly indicate that the global burden of deaths and DALYs due to three behavioral risk factors (high alcohol use, tobacco and low physical activity) in early-onset CRC is declining. It is expected that in the next 30 years, the disease burden of early-onset CRC attributed to high alcohol use and tobacco will further decrease, which may be related to the implementation of advanced global public health policies. The Global Action Plan for the Prevention and Control of Noncommunicable Diseases 2013-2020, issued by World Health Organization (WHO), aims to reduce premature deaths from noncommunicable diseases by targeting multiple risk factors, including smoking, alcohol consumption, and unhealthy diets (28). This initiative has contributed to improvements in the global burden of early-onset CRC.

In the present study, a notable finding is that CRC-related deaths and DALYs attributable to high alcohol use, tobacco and low physical activity in early-onset CRC are higher in GBD regions with higher SDI levels. With ongoing socioeconomic development and urbanization, diets and lifestyles in many countries have undergone significant changes. Populations in high-income areas are more frequently exposed to high-calorie, high-fat diets and are more likely to engage in unhealthy behaviors (excessive alcohol consumption and tobacco use), which may be closely related to the onset of CRC (26, 29). As income levels rise, especially in the high SDI regions, the prevalence of tobacco and alcohol consumption is higher, which may contribute to the rising incidence of early-onset CRC (30). Alcohol is classified as a Group I carcinogen. Its primary metabolite, acetaldehyde, can directly form DNA adducts, leading to DNA double-strand breaks and chromosomal aberrations. In addition, it inhibits DNA repair pathways, thereby increasing the risk of CRC (31). Smoking can induce chronic intestinal inflammation and activate the NF-κB-mediated pro-inflammatory signaling pathway, creating a tumor-promoting microenvironment. At the same time, smoking suppresses antitumor immune surveillance, thereby accelerating the progression and malignant transformation of CRC (32). Although some of these regions may have implemented policies to control tobacco and drinking, unhealthy behaviors remain widespread. Additionally, in some high-income and high SDI regions, individuals may experience greater work-related stress, and prolonged working hours may lead to insufficient physical activity, thereby increasing the risk of CRC (33). Therefore, to reduce the global burden of early-onset CRC, in addition to raising public awareness of unhealthy lifestyle habits, governments and relevant organizations should take proactive measures to promote public awareness of healthy living.

The burden of CRC varies across different genders and age groups. Several studies have pointed out that the risk of CRC is generally higher in males than in females, and this disparity may be related to sex hormones, lifestyle factors, and certain genetic factors (34, 35). Evidence suggests that premenopausal women have a lower risk of CRC, which may be associated with the protective effects of estrogen (36). In many countries, the prevalence of tobacco use and alcohol consumption is higher among males than females, which may contribute to the greater burden of CRC in males (37). Consistent with these findings, our study supports that in males, the proportion of deaths and DALYs related to early-onset CRC attributable to high alcohol use and tobacco is higher in males than in females. An outstanding finding in this study is that the burden of CRC-related deaths and DALYs is higher in females with low physical activity. Additionally, according to the GBD study, the burden of CRC is generally higher in older populations (38). Notably, in recent years, the incidence of CRC among individuals younger than 50 years has been rising, a trend that is especially pronounced in high-income countries (25, 39). Our findings further indicate that early-onset CRC-related deaths and DALYs caused by these three behavioral risk factors increase with age, with the highest burden observed in the 45–49 age group. CRC patients in this age group often don’t undergo routine screening, as many countries recommend CRC screening beginning at age of 50, which may lead to lower early detection rates in this population (40, 41). Moreover, our results reveal that after 2012, early-onset CRC-related deaths and DALYs due to excessive drinking and tobacco in the 45–49 age group exhibited a continuous decline. In 2012, the WHO strengthened the implementation of the Framework Convention on Tobacco Control, encouraging countries to adopt stricter tobacco control measures, including tobacco bans in public places, warning labels on cigarette packages, and restricting tobacco advertising (42). These policies have been effective in reducing tobacco use, thereby contributing to a reduction in the burden of early-onset CRC caused by tobacco.

Several limitations of the present study should be acknowledged. First, the GBD 2021 database doesn’t provide data on the prevalence and incidence of early-onset CRC attributable to the three behavioral risk factors, which may affect the accuracy of the epidemiological trend estimates for this disease. Second, due to the absence of obvious symptoms in younger individuals, early-onset CRC cases may be underdiagnosed, and data reported in the GBD database may introduce bias in estimating attributable burden of CRC. Third, the interactions between CRC comorbidities and risk factors in younger individuals remain unknown, highlighting the need for further well-designed studies. Fourth, the data used in this study were derived from population-level aggregated data from the GBD database rather than individual-level data. Consequently, potential confounding factors could not be adequately adjusted for, and the analysis may be subject to risks of ecological fallacy and measurement bias. Moreover, the use of aggregated data may obscure heterogeneity across regions and within countries, making it difficult to capture true differences across more granular dimensions. In addition, the BAPC forecasting model employed in this study generates projections under the assumption that the current trends in risk factor exposure remain unchanged. Should these trends shift in the future, the predictive estimates may therefore be subject to uncertainty. Finally, this study did not examine potential interactions among different risk factors, and thus the joint effects of multiple risk factors could not be clearly identified, representing a limitation in the analytical scope. Despite these limitations, our findings provide valuable insights that may inform the development of public health policies aimed at addressing early-onset CRC.

## 5 Conclusions

In summary, the global burden of early-onset CRC attributable to high alcohol use, tobacco and low physical activity has shown an overall downward trend from 1990 to 2021. Except for the relatively stable trend associated with low physical activity, the burdens attributable to high alcohol use and tobacco are expected to decline further over the next 30 years. In addition, early-onset CRC-related deaths and DALYs attributable to three behavioral risk factors were increased with age. These findings provide critical support for mitigating the global burden of early-onset CRC attributable to these behavioral risk factors.

## Data Availability

Publicly available datasets were analyzed in this study. This data can be found here: [http://ghdx.healthdata.org/]. - GBD 2021.

## Supplementary material

Supplementary Figure 1 The EAPC map of early-onset CRC attributable to three behavioral risk factors across 204 countries and territories in 2021. **(A)** Deaths caused by high alcohol use. **(B)** DALYs caused by high alcohol use. **(C)** Deaths caused by tobacco. **(D)** DALYs caused by tobacco. **(E)** Deaths caused by low physical activity. **(F)** DALYs caused by low physical activity. CRC, colorectal cancer; EAPC, estimated annual percentage change; DALYs, disability-adjusted life years.

Supplementary Figure 2 Fraction of early-onset CRC attributable to three behavioral risk factors by sex and SDI regions in 1990 and 2021. **(A)** Deaths. **(B)** DALYs. CRC, colorectal cancer; DALYs, disability-adjusted life years; SDI, socio-demographic index.

Supplementary Figure 3 The age-specific numbers of early-onset CRC attributable to three behavioral risk factors. **(A)** Deaths. **(B)** DALYs. CRC, colorectal cancer; DALYs, disability-adjusted life years; SDI, socio-demographic index.

Supplementary Table 1 The global burden of early-onset colorectal cancer attributable to high alcohol use in 204 countries and territories.

Supplementary Table 2 The global burden of early-onset colorectal cancer attributable to tobacco in 204 countries and territories.

Supplementary Table 3 The global burden of early-onset colorectal cancer attributable to low physical activity in 204 countries and territories.
