# Supplementary figures and images for: Global burden of early-onset colorectal cancer related to alcohol, tobacco, and physical inactivity: evidence from the global burden of disease 2021
Source: Front Oncol. 2026 Apr 21;16:1653676. doi: 10.3389/fonc.2026.1653676 (PMC13138888; doi:10.3389/fonc.2026.1653676)

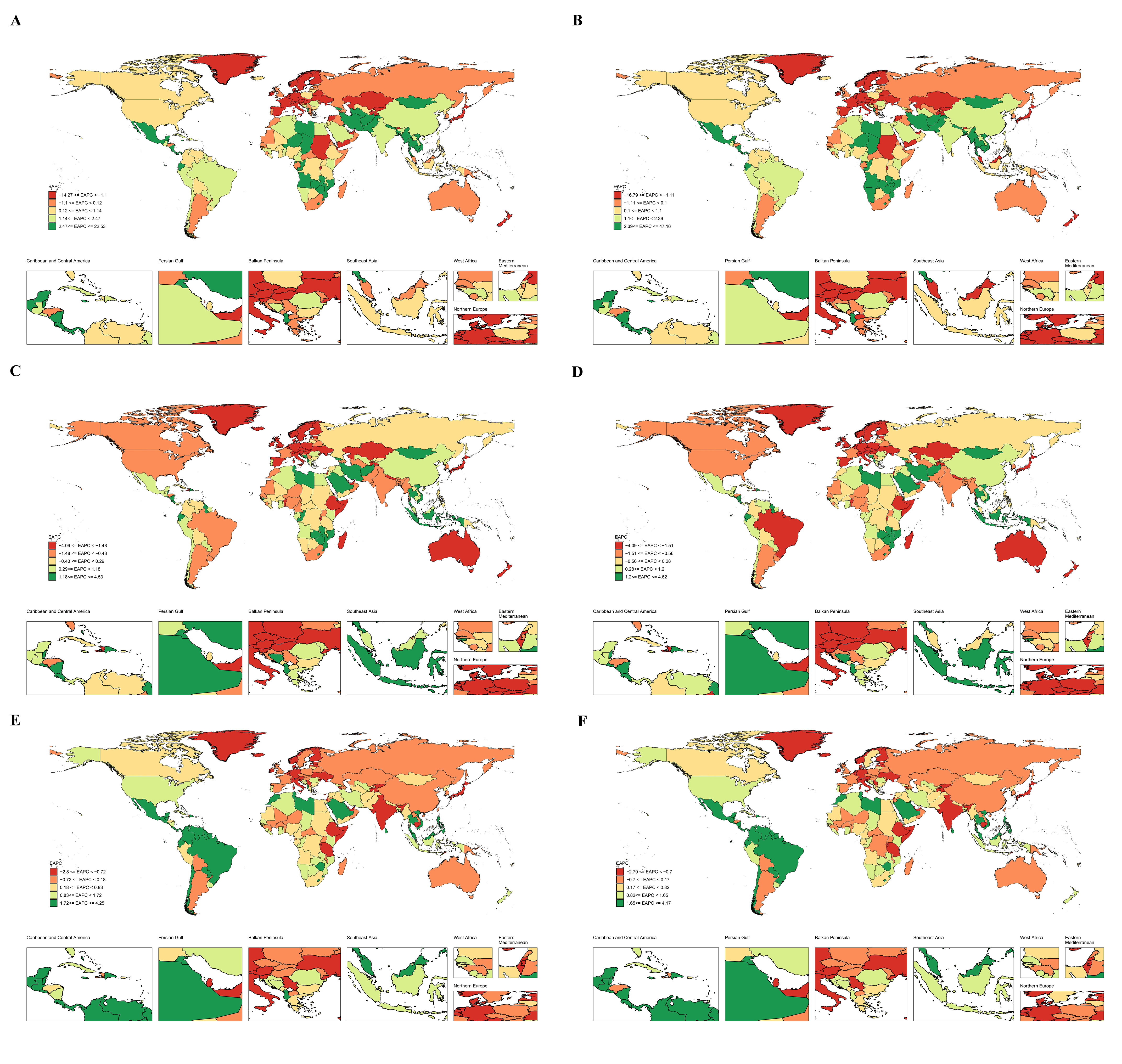

Supplement: Supplementary Figure 1 — The EAPC map of early-onset CRC attributable to three behavioral risk factors across 204 countries and territories in 2021. (A) Deaths caused by high alcohol use. (B) DALYs caused by high alcohol use. (C) Deaths caused by tobacco. (D) DALYs caused by tobacco. (E) Deaths caused by low physical activity. (F) DALYs caused by low physical activity. CRC, colorectal cancer; EAPC, estimated annual percentage change; DALYs, disability-adjusted life years. [file Image1.jpeg]
