# Supplementary material for: Global burden of early-onset colorectal cancer related to alcohol, tobacco, and physical inactivity: evidence from the global burden of disease 2021
Source: Front Oncol. 2026 Apr 21;16:1653676. doi: 10.3389/fonc.2026.1653676 (PMC13138888; doi:10.3389/fonc.2026.1653676)
Supplement: Supplementary Table 1 — The global burden of early-onset colorectal cancer attributable to high alcohol use in 204 countries and territories. [file Table1.docx]

**Supplementary Table 1** The global burden of early-onset colorectal cancer attributable to high alcohol use in 204 countries and territories.

| **Location name** | **1990** | | **2021** | | **EAPC (95% CI)** |
| --- | --- | --- | --- | --- | --- |
|  | **Number** | **ASR** | **Number** | **ASR** |  |
| **Deaths** |  |  |  |  |  |
| Afghanistan | 0 (0-0) | 0 (0-0) | 0 (0-0) | 0.002 (0.001-0.003) | 14.53 (12.09 to 17.03) |
| Albania | 0 (0-1) | 0.019 (0.01-0.03) | 1 (0-1) | 0.05 (0.029-0.082) | 3.38 (2.41 to 4.35) |
| Algeria | 0 (0-1) | 0.003 (0.002-0.004) | 1 (1-2) | 0.005 (0.004-0.008) | 1.78 (1.61 to 1.94) |
| American Samoa | 0 (0-0) | 0.018 (0.003-0.045) | 0 (0-0) | 0.02 (0.004-0.049) | 0.53 (-0.2 to 1.26) |
| Andorra | 0 (0-0) | 0.376 (0.246-0.567) | 0 (0-0) | 0.289 (0.165-0.435) | -0.48 (-0.73 to -0.23) |
| Angola | 2 (1-3) | 0.039 (0.014-0.065) | 11 (6-17) | 0.076 (0.042-0.119) | 3.29 (2.8 to 3.78) |
| Antigua and Barbuda | 0 (0-0) | 0.041 (0.019-0.067) | 0 (0-0) | 0.092 (0.068-0.12) | 3.78 (3.2 to 4.37) |
| Argentina | 52 (40-66) | 0.327 (0.253-0.412) | 59 (44-78) | 0.251 (0.186-0.329) | -0.41 (-0.58 to -0.24) |
| Armenia | 2 (1-3) | 0.114 (0.074-0.157) | 2 (1-2) | 0.116 (0.087-0.153) | -0.31 (-0.72 to 0.1) |
| Australia | 33 (26-42) | 0.369 (0.286-0.465) | 34 (25-44) | 0.282 (0.205-0.364) | -0.91 (-1.04 to -0.78) |
| Austria | 15 (12-20) | 0.381 (0.293-0.485) | 7 (5-9) | 0.173 (0.128-0.225) | -2.27 (-2.43 to -2.11) |
| Azerbaijan | 5 (3-7) | 0.129 (0.086-0.182) | 6 (4-8) | 0.102 (0.066-0.147) | -0.74 (-1.28 to -0.19) |
| Bahamas | 0 (0-0) | 0.239 (0.176-0.307) | 1 (0-1) | 0.253 (0.134-0.401) | 0.35 (0.2 to 0.5) |
| Bahrain | 0 (0-0) | 0.019 (0.013-0.028) | 0 (0-0) | 0.016 (0.011-0.025) | -1.36 (-1.73 to -0.98) |
| Bangladesh | 0 (0-1) | 0.001 (0-0.002) | 2 (0-6) | 0.003 (0-0.007) | 4.81 (4.41 to 5.2) |
| Barbados | 0 (0-0) | 0.191 (0.146-0.238) | 0 (0-1) | 0.272 (0.192-0.373) | 1.76 (1.38 to 2.13) |
| Belarus | 15 (11-20) | 0.306 (0.222-0.4) | 12 (9-17) | 0.293 (0.204-0.411) | -1.1 (-1.52 to -0.68) |
| Belgium | 17 (13-22) | 0.342 (0.263-0.438) | 10 (7-13) | 0.192 (0.136-0.253) | -2.11 (-2.44 to -1.78) |
| Belize | 0 (0-0) | 0.043 (0.033-0.055) | 0 (0-0) | 0.094 (0.07-0.122) | 2.87 (2.32 to 3.42) |
| Benin | 0 (0-0) | 0.011 (0.004-0.02) | 1 (1-2) | 0.019 (0.01-0.03) | 1.85 (1.63 to 2.08) |
| Bermuda | 0 (0-0) | 0.387 (0.288-0.504) | 0 (0-0) | 0.31 (0.21-0.441) | -0.38 (-0.57 to -0.2) |
| Bhutan | 0 (0-0) | 0.027 (0.012-0.045) | 0 (0-0) | 0.007 (0.002-0.015) | -6.42 (-7.05 to -5.78) |
| Bolivia (Plurinational State of) | 3 (1-4) | 0.085 (0.048-0.128) | 6 (4-10) | 0.1 (0.062-0.156) | 0.39 (0.1 to 0.68) |
| Bosnia and Herzegovina | 3 (2-4) | 0.108 (0.064-0.153) | 3 (2-4) | 0.198 (0.127-0.283) | 1.87 (1.56 to 2.18) |
| Botswana | 0 (0-1) | 0.062 (0.032-0.103) | 1 (1-2) | 0.079 (0.04-0.135) | 0.25 (-0.09 to 0.59) |
| Brazil | 70 (55-89) | 0.092 (0.071-0.116) | 206 (161-260) | 0.178 (0.138-0.225) | 1.9 (1.58 to 2.23) |
| Brunei Darussalam | 0 (0-0) | 0.053 (0.036-0.076) | 0 (0-0) | 0.017 (0.002-0.034) | -0.61 (-2.39 to 1.2) |
| Bulgaria | 19 (13-25) | 0.449 (0.312-0.599) | 18 (13-24) | 0.606 (0.44-0.802) | 1.02 (0.7 to 1.34) |
| Burkina Faso | 1 (1-1) | 0.024 (0.015-0.036) | 3 (2-5) | 0.03 (0.019-0.044) | 0.86 (0.77 to 0.94) |
| Burundi | 2 (1-3) | 0.091 (0.041-0.142) | 4 (2-6) | 0.057 (0.029-0.099) | -2.39 (-2.72 to -2.06) |
| Cabo Verde | 0 (0-0) | 0.018 (0.009-0.028) | 0 (0-0) | 0.053 (0.032-0.084) | 3.42 (2.78 to 4.06) |
| Cambodia | 1 (1-3) | 0.032 (0.015-0.056) | 17 (10-26) | 0.187 (0.114-0.292) | 5.41 (5.1 to 5.72) |
| Cameroon | 2 (1-3) | 0.039 (0.019-0.061) | 10 (6-16) | 0.065 (0.036-0.103) | 2.14 (1.97 to 2.32) |
| Canada | 35 (26-44) | 0.235 (0.179-0.3) | 44 (32-58) | 0.267 (0.193-0.35) | 0.5 (0.36 to 0.65) |
| Central African Republic | 1 (0-1) | 0.058 (0.022-0.094) | 1 (0-2) | 0.041 (0.015-0.079) | -0.85 (-0.97 to -0.73) |
| Chad | 0 (0-0) | 0.007 (0.001-0.016) | 2 (1-4) | 0.025 (0.007-0.049) | 5.45 (4.94 to 5.97) |
| Chile | 10 (8-13) | 0.146 (0.112-0.185) | 19 (14-25) | 0.2 (0.15-0.258) | 1.65 (1.43 to 1.88) |
| China | 1338 (915-1765) | 0.201 (0.137-0.265) | 1991 (1409-2807) | 0.3 (0.212-0.423) | 1.33 (0.96 to 1.7) |
| Colombia | 16 (12-20) | 0.095 (0.071-0.12) | 30 (20-40) | 0.114 (0.078-0.153) | 0.49 (0.25 to 0.73) |
| Comoros | 0 (0-0) | 0.003 (0-0.007) | 0 (0-0) | 0.009 (0.003-0.018) | 4.4 (4.04 to 4.77) |
| Congo | 1 (0-1) | 0.06 (0.021-0.102) | 3 (1-5) | 0.111 (0.052-0.186) | 2.88 (2.17 to 3.6) |
| Cook Islands | 0 (0-0) | 0.013 (0-0.038) | 0 (0-0) | 0.1 (0.064-0.141) | 8.29 (7.47 to 9.11) |
| Costa Rica | 1 (1-1) | 0.072 (0.053-0.096) | 5 (3-7) | 0.194 (0.131-0.266) | 2.85 (2.46 to 3.25) |
| Côte d'Ivoire | 1 (1-2) | 0.021 (0.01-0.035) | 4 (2-8) | 0.032 (0.018-0.055) | 0.92 (0.71 to 1.13) |
| Croatia | 8 (6-10) | 0.317 (0.229-0.413) | 5 (3-7) | 0.275 (0.186-0.382) | -0.78 (-1.17 to -0.39) |
| Cuba | 6 (5-8) | 0.101 (0.074-0.133) | 8 (6-11) | 0.155 (0.112-0.211) | 1.78 (1.65 to 1.92) |
| Cyprus | 1 (0-1) | 0.131 (0.094-0.175) | 1 (0-1) | 0.104 (0.069-0.151) | -0.78 (-1.2 to -0.36) |
| Czechia | 31 (24-39) | 0.598 (0.465-0.758) | 18 (13-25) | 0.392 (0.28-0.529) | -2.01 (-2.39 to -1.64) |
| Democratic People's Republic of Korea | 19 (11-30) | 0.175 (0.1-0.278) | 25 (15-44) | 0.184 (0.106-0.321) | 0.52 (0.32 to 0.72) |
| Democratic Republic of the Congo | 4 (1-8) | 0.025 (0.008-0.045) | 11 (4-24) | 0.026 (0.01-0.055) | 0.4 (-0.99 to 1.81) |
| Denmark | 10 (8-13) | 0.391 (0.292-0.504) | 4 (3-5) | 0.156 (0.108-0.213) | -3.3 (-3.67 to -2.93) |
| Djibouti | 0 (0-0) | 0.017 (0.002-0.035) | 0 (0-0) | 0.006 (0.001-0.015) | -4.35 (-4.94 to -3.76) |
| Dominica | 0 (0-0) | 0.09 (0.063-0.122) | 0 (0-0) | 0.149 (0.103-0.212) | 2.05 (1.7 to 2.4) |
| Dominican Republic | 2 (2-3) | 0.068 (0.048-0.091) | 7 (5-10) | 0.118 (0.08-0.169) | 2.16 (1.88 to 2.44) |
| Ecuador | 2 (1-3) | 0.037 (0.022-0.052) | 8 (6-12) | 0.09 (0.06-0.132) | 3.35 (2.46 to 4.25) |
| Egypt | 1 (0-1) | 0.003 (0.002-0.004) | 3 (2-4) | 0.005 (0.003-0.007) | 2.16 (1.97 to 2.35) |
| El Salvador | 1 (1-2) | 0.049 (0.035-0.067) | 3 (2-5) | 0.106 (0.073-0.147) | 2.34 (2.06 to 2.63) |
| Equatorial Guinea | 0 (0-0) | 0.04 (0.013-0.077) | 1 (0-1) | 0.088 (0.041-0.151) | 3.07 (2.91 to 3.23) |
| Eritrea | 0 (0-1) | 0.027 (0.005-0.052) | 1 (0-2) | 0.028 (0.008-0.055) | -0.88 (-1.22 to -0.54) |
| Estonia | 2 (2-3) | 0.301 (0.203-0.404) | 2 (1-2) | 0.262 (0.192-0.355) | -1.06 (-1.41 to -0.71) |
| Eswatini | 0 (0-0) | 0.063 (0.035-0.096) | 1 (0-1) | 0.135 (0.069-0.222) | 2.64 (2.04 to 3.24) |
| Ethiopia | 12 (3-23) | 0.055 (0.012-0.107) | 39 (21-63) | 0.07 (0.037-0.114) | 0.58 (-0.24 to 1.41) |
| Fiji | 0 (0-0) | 0.041 (0.022-0.062) | 0 (0-0) | 0.061 (0.033-0.091) | 1.73 (1.11 to 2.35) |
| Finland | 6 (5-8) | 0.248 (0.186-0.318) | 3 (2-4) | 0.131 (0.096-0.172) | -2.09 (-2.37 to -1.81) |
| France | 97 (75-122) | 0.331 (0.256-0.42) | 63 (47-83) | 0.222 (0.167-0.293) | -1.4 (-1.61 to -1.19) |
| Gabon | 1 (0-1) | 0.154 (0.068-0.255) | 1 (1-2) | 0.155 (0.085-0.253) | -0.18 (-0.32 to -0.05) |
| Gambia | 0 (0-0) | 0.005 (0.001-0.009) | 0 (0-0) | 0.011 (0.006-0.019) | 2.55 (1.97 to 3.14) |
| Georgia | 4 (3-6) | 0.157 (0.093-0.232) | 4 (3-5) | 0.222 (0.16-0.29) | 2.53 (2.04 to 3.03) |
| Germany | 181 (142-232) | 0.455 (0.357-0.581) | 92 (71-118) | 0.259 (0.199-0.333) | -1.55 (-1.68 to -1.41) |
| Ghana | 2 (1-3) | 0.025 (0.011-0.039) | 6 (4-10) | 0.036 (0.021-0.056) | 1.08 (0.93 to 1.22) |
| Greece | 9 (7-11) | 0.179 (0.139-0.225) | 8 (6-10) | 0.173 (0.128-0.228) | -0.17 (-0.33 to -0.01) |
| Greenland | 0 (0-0) | 0.57 (0.344-0.825) | 0 (0-0) | 0.346 (0.202-0.525) | -1.12 (-1.4 to -0.84) |
| Grenada | 0 (0-0) | 0.116 (0.084-0.149) | 0 (0-0) | 0.17 (0.12-0.229) | 1.2 (0.95 to 1.45) |
| Guam | 0 (0-0) | 0.051 (0.002-0.13) | 0 (0-0) | 0.183 (0.045-0.322) | 5.07 (4.58 to 5.56) |
| Guatemala | 1 (1-2) | 0.036 (0.027-0.045) | 5 (4-7) | 0.059 (0.043-0.081) | 1.59 (1.28 to 1.89) |
| Guinea | 0 (0-0) | 0.005 (0.001-0.01) | 1 (0-1) | 0.01 (0.004-0.017) | 1.24 (0.75 to 1.74) |
| Guinea-Bissau | 0 (0-0) | 0.036 (0.017-0.058) | 0 (0-1) | 0.045 (0.026-0.069) | 0.79 (0.71 to 0.88) |
| Guyana | 1 (0-1) | 0.126 (0.091-0.167) | 1 (1-1) | 0.189 (0.13-0.266) | 1.75 (1.31 to 2.19) |
| Haiti | 3 (2-5) | 0.106 (0.06-0.161) | 7 (4-12) | 0.107 (0.062-0.175) | 0.27 (0.09 to 0.44) |
| Honduras | 1 (0-1) | 0.026 (0.018-0.037) | 1 (1-2) | 0.024 (0.013-0.041) | -0.92 (-1.26 to -0.58) |
| Hungary | 29 (22-39) | 0.579 (0.437-0.76) | 19 (13-26) | 0.441 (0.308-0.608) | -1.54 (-1.87 to -1.2) |
| Iceland | 0 (0-0) | 0.104 (0.067-0.148) | 0 (0-0) | 0.139 (0.097-0.187) | 0.57 (0.3 to 0.83) |
| India | 77 (40-116) | 0.018 (0.009-0.028) | 254 (169-359) | 0.033 (0.022-0.046) | 2.18 (2 to 2.36) |
| Indonesia | 8 (3-15) | 0.009 (0.003-0.016) | 19 (6-36) | 0.012 (0.004-0.023) | 0.67 (0.47 to 0.87) |
| Iran (Islamic Republic of) | 0 (0-0) | 0 (0-0) | 7 (4-9) | 0.014 (0.009-0.019) | 22.52 (19.45 to 25.67) |
| Iraq | 0 (0-0) | 0.003 (0.002-0.005) | 1 (0-1) | 0.003 (0.002-0.004) | -0.47 (-0.64 to -0.31) |
| Ireland | 5 (4-7) | 0.307 (0.237-0.396) | 5 (3-6) | 0.196 (0.142-0.257) | -1.77 (-2.03 to -1.52) |
| Israel | 1 (1-2) | 0.047 (0.023-0.081) | 3 (2-4) | 0.065 (0.038-0.099) | 1.08 (0.72 to 1.43) |
| Italy | 97 (76-121) | 0.338 (0.264-0.42) | 50 (38-64) | 0.203 (0.153-0.261) | -1.66 (-1.8 to -1.51) |
| Jamaica | 0 (0-1) | 0.038 (0.027-0.051) | 2 (1-2) | 0.104 (0.067-0.157) | 2.68 (2.01 to 3.35) |
| Japan | 253 (193-317) | 0.39 (0.298-0.489) | 136 (100-176) | 0.269 (0.198-0.347) | -1.32 (-1.55 to -1.09) |
| Jordan | 0 (0-0) | 0.004 (0.002-0.005) | 0 (0-0) | 0.004 (0.003-0.007) | 1.14 (0.79 to 1.49) |
| Kazakhstan | 23 (16-31) | 0.277 (0.199-0.374) | 15 (11-19) | 0.157 (0.117-0.207) | -2.06 (-2.36 to -1.75) |
| Kenya | 3 (1-4) | 0.027 (0.012-0.041) | 11 (6-16) | 0.041 (0.024-0.063) | 1.53 (1.31 to 1.75) |
| Kiribati | 0 (0-0) | 0.033 (0.007-0.066) | 0 (0-0) | 0.021 (0.003-0.053) | -2.3 (-2.75 to -1.85) |
| Kuwait | 0 (0-0) | 0 (0-0) | 0 (0-0) | 0.002 (0-0.004) | 14.25 (12.03 to 16.52) |
| Kyrgyzstan | 3 (2-3) | 0.121 (0.087-0.167) | 3 (2-4) | 0.09 (0.061-0.13) | -1.12 (-1.42 to -0.83) |
| Lao People's Democratic Republic | 2 (1-4) | 0.131 (0.059-0.224) | 9 (5-13) | 0.214 (0.13-0.327) | 1.6 (1.31 to 1.89) |
| Latvia | 4 (3-5) | 0.291 (0.205-0.383) | 3 (2-4) | 0.34 (0.248-0.448) | -0.04 (-0.27 to 0.2) |
| Lebanon | 1 (0-1) | 0.047 (0.025-0.074) | 1 (1-1) | 0.028 (0.019-0.042) | -1.33 (-1.63 to -1.04) |
| Lesotho | 0 (0-0) | 0.038 (0.019-0.064) | 1 (1-2) | 0.118 (0.062-0.187) | 4.08 (3.62 to 4.55) |
| Liberia | 0 (0-0) | 0.031 (0.018-0.045) | 1 (1-2) | 0.037 (0.018-0.066) | 0.44 (-0.05 to 0.93) |
| Libya | 0 (0-0) | 0 (0-0) | 0 (0-1) | 0.012 (0.007-0.019) | 11.14 (8.46 to 13.9) |
| Lithuania | 5 (3-6) | 0.269 (0.188-0.353) | 4 (3-5) | 0.308 (0.222-0.405) | 0.68 (0.24 to 1.12) |
| Luxembourg | 1 (1-1) | 0.458 (0.361-0.575) | 1 (0-1) | 0.163 (0.12-0.215) | -3.36 (-3.65 to -3.07) |
| Madagascar | 1 (0-2) | 0.028 (0.008-0.047) | 3 (1-6) | 0.023 (0.01-0.039) | -0.67 (-1.09 to -0.25) |
| Malawi | 0 (0-1) | 0.011 (0.004-0.017) | 2 (1-4) | 0.021 (0.012-0.037) | 2.36 (2.2 to 2.51) |
| Malaysia | 4 (3-6) | 0.048 (0.028-0.069) | 8 (4-11) | 0.043 (0.024-0.064) | -1.08 (-1.61 to -0.54) |
| Maldives | 0 (0-0) | 0.004 (0-0.012) | 0 (0-0) | 0.009 (0.003-0.018) | -0.19 (-1.77 to 1.41) |
| Mali | 0 (0-0) | 0.007 (0.004-0.012) | 1 (0-1) | 0.008 (0.005-0.012) | 0.44 (0.34 to 0.55) |
| Malta | 0 (0-0) | 0.141 (0.098-0.186) | 0 (0-0) | 0.148 (0.102-0.202) | -0.49 (-0.77 to -0.21) |
| Marshall Islands | 0 (0-0) | 0.044 (0.021-0.073) | 0 (0-0) | 0.074 (0.035-0.12) | 1.61 (1.5 to 1.71) |
| Mauritania | 0 (0-0) | 0 (0-0) | 0 (0-0) | 0 (0-0) | 0 (0 to 0) |
| Mauritius | 0 (0-1) | 0.06 (0.039-0.084) | 1 (1-1) | 0.166 (0.111-0.231) | 2.12 (1.55 to 2.7) |
| Mexico | 26 (20-33) | 0.061 (0.047-0.077) | 110 (84-143) | 0.161 (0.122-0.209) | 3.06 (2.85 to 3.27) |
| Micronesia (Federated States of) | 0 (0-0) | 0.094 (0.051-0.148) | 0 (0-0) | 0.067 (0.034-0.113) | -1.42 (-1.54 to -1.3) |
| Monaco | 0 (0-0) | 0.299 (0.011-0.626) | 0 (0-0) | 0.345 (0.021-0.731) | 0.64 (0.43 to 0.86) |
| Mongolia | 0 (0-1) | 0.045 (0.025-0.07) | 3 (2-4) | 0.164 (0.109-0.232) | 5.35 (4.97 to 5.74) |
| Montenegro | 1 (0-1) | 0.17 (0.117-0.241) | 1 (0-1) | 0.187 (0.131-0.272) | 0.12 (-0.24 to 0.48) |
| Morocco | 1 (0-1) | 0.006 (0.004-0.009) | 1 (1-2) | 0.006 (0.003-0.01) | -0.11 (-0.32 to 0.1) |
| Mozambique | 0 (0-0) | 0.002 (0-0.004) | 1 (0-2) | 0.008 (0.003-0.014) | 5.68 (5.49 to 5.88) |
| Myanmar | 2 (1-4) | 0.011 (0.003-0.022) | 30 (18-47) | 0.102 (0.062-0.159) | 8.74 (8.19 to 9.29) |
| Namibia | 0 (0-0) | 0.041 (0.018-0.065) | 1 (1-2) | 0.09 (0.048-0.143) | 2.47 (2.04 to 2.89) |
| Nauru | 0 (0-0) | 0.156 (0.057-0.306) | 0 (0-0) | 0.188 (0.094-0.295) | 0.65 (0.47 to 0.83) |
| Nepal | 0 (0-0) | 0.002 (0-0.006) | 4 (2-7) | 0.024 (0.01-0.042) | 8.16 (7.37 to 8.96) |
| Netherlands | 25 (19-32) | 0.305 (0.237-0.398) | 16 (12-22) | 0.216 (0.16-0.294) | -1.14 (-1.33 to -0.95) |
| New Zealand | 9 (7-11) | 0.476 (0.365-0.602) | 7 (5-9) | 0.292 (0.216-0.378) | -1.44 (-1.61 to -1.26) |
| Nicaragua | 1 (0-1) | 0.033 (0.022-0.047) | 2 (2-3) | 0.065 (0.044-0.092) | 2.66 (2.41 to 2.9) |
| Niger | 0 (0-0) | 0.001 (0-0.002) | 0 (0-0) | 0.002 (0-0.004) | 2.96 (2.26 to 3.66) |
| Nigeria | 10 (6-15) | 0.025 (0.015-0.037) | 32 (19-49) | 0.03 (0.018-0.046) | 0.76 (0.65 to 0.86) |
| Niue | 0 (0-0) | 0.092 (0.016-0.176) | 0 (0-0) | 0.141 (0.059-0.226) | 0.68 (0.4 to 0.96) |
| North Macedonia | 2 (1-3) | 0.195 (0.141-0.259) | 2 (1-3) | 0.161 (0.11-0.232) | -1.07 (-1.36 to -0.79) |
| Northern Mariana Islands | 0 (0-0) | 0.077 (0.002-0.185) | 0 (0-0) | 0.119 (0.017-0.221) | 1.56 (1.26 to 1.86) |
| Norway | 5 (3-7) | 0.225 (0.159-0.31) | 4 (3-5) | 0.161 (0.116-0.212) | -1.28 (-1.6 to -0.95) |
| Oman | 0 (0-0) | 0.002 (0.001-0.003) | 0 (0-0) | 0.002 (0.001-0.004) | 0 (-0.86 to 0.86) |
| Pakistan | 2 (0-3) | 0.004 (0.001-0.007) | 11 (5-19) | 0.009 (0.004-0.016) | 3.16 (2.92 to 3.4) |
| Palau | 0 (0-0) | 0.037 (0.006-0.084) | 0 (0-0) | 0.071 (0.017-0.139) | 2.12 (2.02 to 2.21) |
| Palestine | 0 (0-0) | 0.023 (0.014-0.033) | 1 (0-1) | 0.02 (0.013-0.028) | -0.66 (-0.9 to -0.43) |
| Panama | 1 (1-1) | 0.074 (0.056-0.094) | 3 (2-4) | 0.15 (0.106-0.199) | 2.53 (2.16 to 2.9) |
| Papua New Guinea | 0 (0-0) | 0.015 (0.007-0.024) | 1 (0-1) | 0.012 (0.007-0.018) | -0.61 (-1.15 to -0.07) |
| Paraguay | 2 (1-2) | 0.085 (0.062-0.114) | 5 (3-7) | 0.134 (0.089-0.186) | 1.35 (1.17 to 1.54) |
| Peru | 9 (5-13) | 0.082 (0.05-0.118) | 23 (15-33) | 0.117 (0.076-0.173) | 1.5 (1.21 to 1.79) |
| Philippines | 61 (43-78) | 0.196 (0.138-0.251) | 162 (119-209) | 0.27 (0.197-0.348) | 1.07 (0.98 to 1.16) |
| Poland | 57 (44-72) | 0.302 (0.234-0.381) | 64 (49-81) | 0.358 (0.275-0.453) | 0.22 (0 to 0.43) |
| Portugal | 19 (15-24) | 0.383 (0.293-0.481) | 15 (11-20) | 0.335 (0.25-0.438) | -0.41 (-0.68 to -0.14) |
| Puerto Rico | 3 (3-5) | 0.189 (0.138-0.245) | 3 (2-4) | 0.227 (0.165-0.301) | 0.38 (0.12 to 0.63) |
| Qatar | 0 (0-0) | 0.009 (0.006-0.014) | 0 (0-0) | 0.01 (0.006-0.014) | 0.23 (-0.46 to 0.93) |
| Republic of Korea | 65 (47-86) | 0.251 (0.181-0.332) | 52 (37-72) | 0.215 (0.151-0.296) | -0.8 (-1.06 to -0.55) |
| Republic of Moldova | 8 (7-11) | 0.383 (0.297-0.483) | 6 (4-8) | 0.335 (0.251-0.444) | -0.73 (-0.96 to -0.5) |
| Romania | 30 (23-41) | 0.268 (0.201-0.36) | 35 (26-47) | 0.423 (0.307-0.56) | 1.29 (1.03 to 1.54) |
| Russian Federation | 226 (181-279) | 0.305 (0.243-0.376) | 215 (171-268) | 0.319 (0.253-0.398) | -0.41 (-0.85 to 0.03) |
| Rwanda | 4 (2-6) | 0.12 (0.05-0.186) | 6 (3-9) | 0.084 (0.046-0.137) | -2.64 (-3.17 to -2.12) |
| Saint Kitts and Nevis | 0 (0-0) | 0.101 (0.003-0.144) | 0 (0-0) | 0.075 (0.001-0.183) | -1.58 (-1.95 to -1.21) |
| Saint Lucia | 0 (0-0) | 0.149 (0.117-0.184) | 0 (0-0) | 0.205 (0.149-0.273) | 1.28 (1.11 to 1.44) |
| Saint Vincent and the Grenadines | 0 (0-0) | 0.087 (0.058-0.119) | 0 (0-0) | 0.243 (0.182-0.323) | 3.59 (3.34 to 3.85) |
| Samoa | 0 (0-0) | 0.025 (0.014-0.038) | 0 (0-0) | 0.026 (0.013-0.042) | 0.23 (-0.19 to 0.66) |
| San Marino | 0 (0-0) | 0.235 (0.002-0.383) | 0 (0-0) | 0.156 (0.002-0.296) | -0.16 (-0.6 to 0.29) |
| Sao Tome and Principe | 0 (0-0) | 0.036 (0.019-0.057) | 0 (0-0) | 0.066 (0.037-0.111) | 1.67 (1.31 to 2.03) |
| Saudi Arabia | 0 (0-1) | 0.004 (0.001-0.006) | 1 (0-2) | 0.004 (0.001-0.008) | 1.81 (1.2 to 2.43) |
| Senegal | 0 (0-0) | 0.005 (0.001-0.011) | 0 (0-1) | 0.004 (0.001-0.009) | -0.84 (-1.2 to -0.48) |
| Serbia | 13 (9-19) | 0.28 (0.182-0.39) | 10 (7-14) | 0.239 (0.16-0.336) | -0.99 (-1.31 to -0.67) |
| Seychelles | 0 (0-0) | 0.122 (0.082-0.172) | 0 (0-0) | 0.251 (0.169-0.351) | 1.82 (1.16 to 2.49) |
| Sierra Leone | 0 (0-1) | 0.02 (0.011-0.032) | 1 (1-1) | 0.02 (0.011-0.032) | -0.06 (-0.14 to 0.03) |
| Singapore | 2 (1-2) | 0.082 (0.051-0.117) | 2 (1-3) | 0.06 (0.036-0.089) | -2.09 (-2.72 to -1.45) |
| Slovakia | 14 (10-18) | 0.515 (0.387-0.691) | 10 (7-14) | 0.377 (0.263-0.526) | -1.23 (-1.4 to -1.07) |
| Slovenia | 3 (2-4) | 0.318 (0.224-0.433) | 1 (1-2) | 0.13 (0.066-0.218) | -3.5 (-3.73 to -3.26) |
| Solomon Islands | 0 (0-0) | 0.01 (0.003-0.022) | 0 (0-0) | 0.026 (0.011-0.049) | 4.18 (3.43 to 4.93) |
| Somalia | 0 (0-0) | 0 (0-0) | 0 (0-0) | 0 (0-0) | 0 (0 to 0) |
| South Africa | 28 (20-36) | 0.148 (0.104-0.19) | 49 (34-67) | 0.157 (0.11-0.215) | -0.07 (-0.27 to 0.14) |
| South Sudan | 0 (0-0) | 0.004 (0-0.011) | 0 (0-0) | 0.004 (0-0.011) | 0.79 (0.43 to 1.16) |
| Spain | 71 (55-90) | 0.366 (0.283-0.463) | 47 (35-61) | 0.235 (0.173-0.306) | -1.61 (-1.77 to -1.45) |
| Sri Lanka | 2 (1-3) | 0.023 (0.015-0.033) | 4 (2-6) | 0.035 (0.02-0.055) | 1.08 (0.63 to 1.53) |
| Sudan | 2 (1-3) | 0.02 (0.011-0.033) | 0 (0-0) | 0 (0-0) | -14.26 (-17.26 to -11.16) |
| Suriname | 0 (0-0) | 0.125 (0.083-0.169) | 0 (0-1) | 0.16 (0.103-0.227) | 1.04 (0.77 to 1.32) |
| Sweden | 11 (8-14) | 0.263 (0.2-0.344) | 7 (5-9) | 0.158 (0.112-0.208) | -1.29 (-1.45 to -1.13) |
| Switzerland | 10 (7-12) | 0.264 (0.206-0.328) | 4 (3-6) | 0.111 (0.083-0.148) | -2.66 (-2.89 to -2.42) |
| Syrian Arab Republic | 1 (0-1) | 0.014 (0.009-0.021) | 1 (0-1) | 0.008 (0.004-0.014) | -1.73 (-1.93 to -1.53) |
| Taiwan (Province of China) | 31 (24-38) | 0.272 (0.21-0.34) | 32 (23-41) | 0.28 (0.206-0.365) | -0.61 (-0.95 to -0.27) |
| Tajikistan | 2 (1-2) | 0.063 (0.043-0.092) | 2 (1-4) | 0.039 (0.024-0.079) | -2.35 (-3.15 to -1.55) |
| Thailand | 40 (27-56) | 0.125 (0.086-0.176) | 114 (74-165) | 0.357 (0.231-0.517) | 2.91 (2.5 to 3.31) |
| Timor-Leste | 0 (0-0) | 0.022 (0.011-0.039) | 0 (0-0) | 0.043 (0.024-0.065) | 1.65 (1.2 to 2.1) |
| Togo | 0 (0-0) | 0.016 (0.008-0.027) | 1 (0-2) | 0.024 (0.012-0.041) | 1.84 (1.5 to 2.18) |
| Tokelau | 0 (0-0) | 0.037 (0.013-0.066) | 0 (0-0) | 0.074 (0.034-0.126) | 2.06 (1.96 to 2.16) |
| Tonga | 0 (0-0) | 0.009 (0.003-0.015) | 0 (0-0) | 0.009 (0.003-0.017) | 0.1 (-0.76 to 0.97) |
| Trinidad and Tobago | 1 (1-1) | 0.128 (0.096-0.165) | 2 (1-2) | 0.225 (0.152-0.319) | 2.17 (1.89 to 2.46) |
| Tunisia | 0 (0-1) | 0.012 (0.008-0.017) | 2 (1-2) | 0.025 (0.016-0.037) | 2.26 (2.12 to 2.4) |
| Turkey | 20 (13-29) | 0.07 (0.046-0.101) | 24 (16-34) | 0.055 (0.037-0.077) | -1.29 (-1.61 to -0.97) |
| Turkmenistan | 1 (0-1) | 0.043 (0.024-0.066) | 2 (2-3) | 0.089 (0.06-0.127) | 2.49 (2.14 to 2.85) |
| Tuvalu | 0 (0-0) | 0.036 (0.015-0.066) | 0 (0-0) | 0.054 (0.026-0.094) | 0.96 (0.6 to 1.32) |
| Uganda | 4 (2-7) | 0.06 (0.024-0.094) | 19 (11-30) | 0.095 (0.054-0.15) | 0.83 (0.46 to 1.19) |
| Ukraine | 103 (75-132) | 0.413 (0.303-0.528) | 62 (38-89) | 0.309 (0.188-0.442) | -1.51 (-1.92 to -1.09) |
| United Arab Emirates | 1 (0-1) | 0.066 (0.035-0.105) | 2 (1-4) | 0.033 (0.019-0.059) | -2.62 (-3.04 to -2.19) |
| United Kingdom | 91 (72-114) | 0.318 (0.252-0.399) | 77 (59-97) | 0.254 (0.193-0.321) | -0.83 (-0.98 to -0.68) |
| United Republic of Tanzania | 5 (2-8) | 0.047 (0.021-0.073) | 17 (10-27) | 0.059 (0.035-0.094) | 0.86 (0.78 to 0.94) |
| United States of America | 313 (248-396) | 0.233 (0.185-0.295) | 394 (306-493) | 0.259 (0.201-0.324) | 0.45 (0.34 to 0.55) |
| United States Virgin Islands | 0 (0-0) | 0.203 (0.002-0.434) | 0 (0-0) | 0.319 (0.02-0.613) | 2.01 (1.84 to 2.18) |
| Uruguay | 4 (3-6) | 0.298 (0.225-0.392) | 5 (4-7) | 0.318 (0.228-0.426) | 0.33 (0.21 to 0.45) |
| Uzbekistan | 5 (3-8) | 0.055 (0.034-0.079) | 12 (8-16) | 0.068 (0.047-0.091) | 0.22 (-0.37 to 0.82) |
| Vanuatu | 0 (0-0) | 0.042 (0.021-0.069) | 0 (0-0) | 0.061 (0.033-0.097) | 1.41 (0.33 to 2.51) |
| Venezuela (Bolivarian Republic of) | 10 (8-13) | 0.108 (0.084-0.134) | 18 (12-27) | 0.14 (0.088-0.204) | 0.87 (0.65 to 1.09) |
| Viet Nam | 2 (0-4) | 0.005 (0.001-0.011) | 105 (67-156) | 0.202 (0.129-0.3) | 13.21 (11.94 to 14.48) |
| Yemen | 1 (0-1) | 0.012 (0.006-0.02) | 1 (0-1) | 0.004 (0.002-0.008) | -3.49 (-3.78 to -3.2) |
| Zambia | 2 (1-3) | 0.058 (0.023-0.091) | 12 (5-33) | 0.126 (0.056-0.345) | 2.85 (2.55 to 3.15) |
| Zimbabwe | 2 (1-4) | 0.052 (0.03-0.079) | 8 (4-14) | 0.109 (0.057-0.18) | 2.5 (2.18 to 2.81) |
| **Disability-adjusted life years** |  |  |  |  |  |
| Afghanistan | 0 (0-0) | 0 (0-0) | 12 (5-23) | 0.079 (0.031-0.159) | 47.15 (37.43 to 57.56) |
| Albania | 16 (9-27) | 0.957 (0.525-1.567) | 31 (18-51) | 2.494 (1.458-4.079) | 3.18 (2.23 to 4.15) |
| Algeria | 19 (13-27) | 0.165 (0.114-0.233) | 63 (43-93) | 0.278 (0.188-0.41) | 1.72 (1.55 to 1.89) |
| American Samoa | 0 (0-1) | 0.947 (0.152-2.358) | 0 (0-1) | 1.016 (0.218-2.515) | 0.38 (-0.33 to 1.09) |
| Andorra | 6 (4-9) | 18.803 (12.401-27.968) | 6 (3-9) | 14.29 (8.177-21.354) | -0.52 (-0.76 to -0.27) |
| Angola | 90 (33-150) | 1.94 (0.711-3.225) | 562 (312-877) | 3.835 (2.125-5.984) | 3.33 (2.83 to 3.83) |
| Antigua and Barbuda | 1 (0-1) | 2.134 (0.985-3.487) | 2 (2-3) | 4.645 (3.41-6.019) | 3.66 (3.07 to 4.25) |
| Argentina | 2576 (1999-3255) | 16.21 (12.58-20.48) | 2969 (2191-3890) | 12.612 (9.308-16.526) | -0.33 (-0.52 to -0.15) |
| Armenia | 103 (66-142) | 6.012 (3.89-8.298) | 86 (64-113) | 5.925 (4.411-7.763) | -0.44 (-0.87 to 0) |
| Australia | 1651 (1279-2081) | 18.375 (14.231-23.154) | 1737 (1273-2243) | 14.465 (10.603-18.683) | -0.79 (-0.92 to -0.67) |
| Austria | 751 (581-954) | 18.667 (14.428-23.688) | 351 (261-458) | 8.701 (6.461-11.345) | -2.24 (-2.4 to -2.09) |
| Azerbaijan | 252 (168-355) | 6.853 (4.568-9.66) | 291 (188-416) | 5.226 (3.376-7.47) | -0.96 (-1.57 to -0.36) |
| Bahamas | 18 (13-23) | 12.347 (9.17-15.81) | 27 (15-43) | 12.923 (6.928-20.517) | 0.27 (0.12 to 0.42) |
| Bahrain | 3 (2-4) | 1.013 (0.7-1.465) | 8 (6-12) | 0.851 (0.563-1.261) | -1.34 (-1.71 to -0.97) |
| Bangladesh | 14 (0-44) | 0.029 (0-0.089) | 122 (7-288) | 0.138 (0.008-0.328) | 5.12 (4.68 to 5.56) |
| Barbados | 13 (10-17) | 9.952 (7.64-12.424) | 19 (14-26) | 13.8 (9.727-18.855) | 1.6 (1.26 to 1.95) |
| Belarus | 759 (551-995) | 15.054 (10.923-19.724) | 614 (427-855) | 14.42 (10.04-20.105) | -1.11 (-1.51 to -0.71) |
| Belgium | 847 (651-1081) | 17.074 (13.119-21.8) | 482 (346-633) | 9.658 (6.935-12.671) | -2.07 (-2.39 to -1.76) |
| Belize | 2 (1-2) | 2.315 (1.751-2.916) | 12 (9-15) | 4.884 (3.607-6.344) | 2.78 (2.24 to 3.32) |
| Benin | 11 (4-20) | 0.568 (0.188-0.996) | 59 (30-96) | 0.937 (0.479-1.533) | 1.88 (1.65 to 2.1) |
| Bermuda | 7 (5-9) | 19.571 (14.624-25.432) | 4 (3-6) | 15.817 (10.857-22.195) | -0.37 (-0.55 to -0.18) |
| Bhutan | 4 (2-7) | 1.361 (0.602-2.275) | 1 (0-3) | 0.335 (0.093-0.776) | -6.43 (-7.06 to -5.8) |
| Bolivia (Plurinational State of) | 130 (72-196) | 4.341 (2.418-6.539) | 317 (197-495) | 5.068 (3.156-7.907) | 0.39 (0.09 to 0.68) |
| Bosnia and Herzegovina | 130 (78-184) | 5.411 (3.238-7.668) | 143 (93-205) | 9.611 (6.24-13.746) | 1.76 (1.48 to 2.05) |
| Botswana | 19 (10-31) | 3.143 (1.681-5.206) | 54 (27-94) | 3.988 (2.01-6.908) | 0.23 (-0.1 to 0.56) |
| Brazil | 3660 (2861-4621) | 4.776 (3.733-6.031) | 10340 (8059-13044) | 8.916 (6.949-11.248) | 1.81 (1.5 to 2.12) |
| Brunei Darussalam | 4 (3-6) | 2.766 (1.919-3.943) | 2 (0-5) | 0.866 (0.115-1.667) | -0.81 (-2.6 to 1.01) |
| Bulgaria | 907 (628-1207) | 21.816 (15.106-29.044) | 858 (625-1131) | 29.205 (21.285-38.503) | 1.02 (0.7 to 1.34) |
| Burkina Faso | 45 (28-68) | 1.178 (0.726-1.762) | 158 (98-229) | 1.528 (0.947-2.22) | 0.91 (0.83 to 1) |
| Burundi | 112 (49-173) | 4.6 (2.038-7.14) | 180 (93-319) | 2.885 (1.495-5.116) | -2.32 (-2.64 to -1.99) |
| Cabo Verde | 1 (1-2) | 0.976 (0.496-1.517) | 8 (5-13) | 2.66 (1.592-4.217) | 3.21 (2.58 to 3.84) |
| Cambodia | 75 (36-132) | 1.625 (0.79-2.872) | 863 (532-1349) | 9.573 (5.902-14.967) | 5.38 (5.06 to 5.69) |
| Cameroon | 89 (44-138) | 1.955 (0.98-3.054) | 509 (287-802) | 3.297 (1.861-5.197) | 2.2 (2.03 to 2.38) |
| Canada | 1752 (1342-2223) | 11.885 (9.101-15.073) | 2290 (1656-2980) | 13.767 (9.955-17.914) | 0.56 (0.4 to 0.72) |
| Central African Republic | 36 (14-58) | 2.885 (1.09-4.661) | 54 (20-103) | 2.016 (0.759-3.865) | -0.89 (-1.01 to -0.77) |
| Chad | 8 (1-21) | 0.338 (0.048-0.824) | 93 (25-182) | 1.248 (0.343-2.45) | 5.51 (4.99 to 6.02) |
| Chile | 528 (406-668) | 7.421 (5.71-9.39) | 955 (719-1239) | 10.051 (7.569-13.037) | 1.57 (1.35 to 1.8) |
| China | 68107 (46556-89761) | 10.213 (6.981-13.46) | 100558 (71354-141121) | 15.16 (10.757-21.276) | 1.27 (0.9 to 1.65) |
| Colombia | 869 (653-1106) | 5.136 (3.859-6.538) | 1581 (1084-2124) | 6.056 (4.149-8.133) | 0.45 (0.19 to 0.7) |
| Comoros | 0 (0-1) | 0.136 (0.013-0.353) | 2 (1-4) | 0.456 (0.141-0.908) | 4.41 (4.03 to 4.79) |
| Congo | 33 (11-57) | 2.998 (1.036-5.113) | 155 (73-260) | 5.506 (2.591-9.234) | 2.82 (2.13 to 3.52) |
| Cook Islands | 0 (0-0) | 0.698 (0-1.965) | 0 (0-1) | 5.048 (3.213-7.161) | 8.13 (7.34 to 8.92) |
| Costa Rica | 58 (42-77) | 3.773 (2.736-5.021) | 252 (170-345) | 10.058 (6.796-13.776) | 2.87 (2.46 to 3.29) |
| Côte d'Ivoire | 59 (28-97) | 1.061 (0.503-1.751) | 219 (125-379) | 1.6 (0.912-2.767) | 0.92 (0.72 to 1.13) |
| Croatia | 380 (275-497) | 15.577 (11.268-20.339) | 246 (168-341) | 13.437 (9.161-18.65) | -0.8 (-1.16 to -0.43) |
| Cuba | 323 (236-425) | 5.245 (3.836-6.904) | 395 (289-542) | 7.779 (5.685-10.672) | 1.6 (1.46 to 1.73) |
| Cyprus | 26 (19-35) | 6.475 (4.659-8.79) | 37 (25-54) | 5.266 (3.485-7.643) | -0.71 (-1.14 to -0.27) |
| Czechia | 1502 (1165-1904) | 28.84 (22.372-36.559) | 904 (642-1218) | 19.163 (13.615-25.818) | -1.88 (-2.22 to -1.53) |
| Democratic People's Republic of Korea | 938 (542-1506) | 8.733 (5.047-14.014) | 1261 (724-2201) | 9.119 (5.235-15.912) | 0.42 (0.23 to 0.6) |
| Democratic Republic of the Congo | 217 (71-383) | 1.291 (0.424-2.281) | 575 (218-1209) | 1.328 (0.503-2.791) | 0.37 (-1.03 to 1.78) |
| Denmark | 510 (383-651) | 19.092 (14.313-24.362) | 198 (138-269) | 7.688 (5.357-10.451) | -3.26 (-3.63 to -2.9) |
| Djibouti | 2 (0-4) | 0.862 (0.114-1.744) | 2 (0-5) | 0.307 (0.043-0.735) | -4.32 (-4.91 to -3.73) |
| Dominica | 2 (1-2) | 4.599 (3.192-6.172) | 3 (2-4) | 7.519 (5.13-10.513) | 1.97 (1.64 to 2.31) |
| Dominican Republic | 131 (91-176) | 3.595 (2.509-4.832) | 357 (243-509) | 6.095 (4.145-8.676) | 2.08 (1.8 to 2.36) |
| Ecuador | 97 (56-135) | 1.952 (1.138-2.719) | 439 (294-645) | 4.67 (3.131-6.863) | 3.32 (2.43 to 4.23) |
| Egypt | 41 (25-59) | 0.153 (0.094-0.22) | 133 (82-190) | 0.249 (0.153-0.355) | 2.26 (2.06 to 2.47) |
| El Salvador | 65 (47-89) | 2.608 (1.871-3.563) | 181 (125-254) | 5.481 (3.763-7.658) | 2.3 (2.01 to 2.58) |
| Equatorial Guinea | 4 (1-7) | 1.987 (0.658-3.74) | 37 (17-63) | 4.542 (2.119-7.824) | 3.22 (3.06 to 3.38) |
| Eritrea | 20 (4-39) | 1.315 (0.234-2.544) | 49 (14-95) | 1.435 (0.404-2.775) | -0.81 (-1.16 to -0.46) |
| Estonia | 113 (76-152) | 14.922 (10.046-20.079) | 74 (54-100) | 12.906 (9.426-17.466) | -1.07 (-1.41 to -0.74) |
| Eswatini | 11 (6-17) | 3.12 (1.739-4.678) | 42 (21-67) | 6.79 (3.438-10.929) | 2.7 (2.1 to 3.3) |
| Ethiopia | 609 (135-1180) | 2.803 (0.62-5.437) | 2006 (1055-3203) | 3.644 (1.918-5.82) | 0.68 (-0.13 to 1.49) |
| Fiji | 8 (5-13) | 2.127 (1.181-3.239) | 15 (8-22) | 3.133 (1.706-4.654) | 1.69 (1.06 to 2.32) |
| Finland | 318 (239-407) | 12.304 (9.248-15.764) | 154 (114-201) | 6.592 (4.86-8.611) | -2.01 (-2.28 to -1.75) |
| France | 4830 (3742-6101) | 16.553 (12.822-20.906) | 3195 (2406-4239) | 11.295 (8.504-14.984) | -1.31 (-1.49 to -1.12) |
| Gabon | 34 (16-57) | 7.701 (3.47-12.65) | 71 (40-116) | 7.693 (4.276-12.488) | -0.23 (-0.36 to -0.09) |
| Gambia | 1 (0-2) | 0.243 (0.077-0.443) | 7 (4-11) | 0.575 (0.307-0.952) | 2.58 (1.99 to 3.19) |
| Georgia | 218 (128-320) | 8.117 (4.786-11.935) | 177 (128-232) | 11.084 (8.002-14.492) | 2.34 (1.84 to 2.85) |
| Germany | 8807 (6990-11196) | 22.082 (17.525-28.069) | 4595 (3553-5855) | 12.916 (9.986-16.458) | -1.52 (-1.64 to -1.4) |
| Ghana | 88 (40-139) | 1.283 (0.581-2.034) | 320 (187-506) | 1.828 (1.067-2.885) | 1.06 (0.92 to 1.2) |
| Greece | 456 (356-570) | 9.022 (7.058-11.277) | 368 (272-484) | 8.512 (6.286-11.177) | -0.25 (-0.43 to -0.07) |
| Greenland | 10 (6-14) | 28.541 (17.289-41.019) | 5 (3-7) | 17.083 (9.908-25.908) | -1.25 (-1.48 to -1.03) |
| Grenada | 2 (2-3) | 6.154 (4.446-7.938) | 5 (3-6) | 8.62 (6.143-11.604) | 1.04 (0.82 to 1.26) |
| Guam | 2 (0-5) | 2.776 (0.099-6.928) | 7 (2-12) | 9.21 (2.337-15.98) | 4.8 (4.34 to 5.25) |
| Guatemala | 68 (52-86) | 1.915 (1.456-2.434) | 262 (189-354) | 3.11 (2.241-4.213) | 1.58 (1.27 to 1.89) |
| Guinea | 7 (2-12) | 0.261 (0.072-0.486) | 29 (13-53) | 0.479 (0.206-0.86) | 1.3 (0.79 to 1.81) |
| Guinea-Bissau | 8 (4-13) | 1.815 (0.864-2.863) | 23 (13-35) | 2.279 (1.309-3.486) | 0.85 (0.76 to 0.93) |
| Guyana | 26 (19-35) | 6.502 (4.731-8.613) | 39 (27-54) | 9.638 (6.634-13.442) | 1.66 (1.24 to 2.08) |
| Haiti | 159 (90-240) | 5.416 (3.054-8.18) | 376 (219-607) | 5.478 (3.193-8.853) | 0.29 (0.11 to 0.46) |
| Honduras | 28 (20-39) | 1.367 (0.954-1.904) | 65 (36-112) | 1.203 (0.672-2.061) | -1.06 (-1.42 to -0.7) |
| Hungary | 1442 (1091-1891) | 28.325 (21.43-37.15) | 925 (647-1275) | 21.316 (14.892-29.37) | -1.54 (-1.85 to -1.24) |
| Iceland | 7 (4-10) | 5.286 (3.393-7.482) | 12 (8-16) | 7.163 (5.015-9.619) | 0.67 (0.41 to 0.92) |
| India | 3820 (2005-5748) | 0.909 (0.477-1.367) | 12572 (8492-17755) | 1.612 (1.089-2.277) | 2.15 (1.98 to 2.32) |
| Indonesia | 435 (156-771) | 0.46 (0.165-0.816) | 942 (302-1805) | 0.614 (0.197-1.177) | 0.58 (0.37 to 0.79) |
| Iran (Islamic Republic of) | 0 (0-0) | 0 (0-0.001) | 348 (234-462) | 0.733 (0.494-0.975) | 33.41 (27.94 to 39.1) |
| Iraq | 14 (10-20) | 0.17 (0.116-0.238) | 29 (19-47) | 0.132 (0.084-0.212) | -0.53 (-0.68 to -0.37) |
| Ireland | 271 (212-347) | 15.218 (11.882-19.457) | 228 (165-300) | 9.878 (7.152-12.979) | -1.68 (-1.94 to -1.42) |
| Israel | 58 (29-99) | 2.391 (1.174-4.08) | 145 (85-223) | 3.234 (1.887-4.967) | 1.1 (0.74 to 1.45) |
| Italy | 4782 (3740-5932) | 16.651 (13.02-20.653) | 2470 (1862-3176) | 10.036 (7.566-12.905) | -1.66 (-1.82 to -1.51) |
| Jamaica | 23 (16-30) | 1.956 (1.358-2.614) | 82 (52-123) | 5.326 (3.419-8.01) | 2.57 (1.9 to 3.25) |
| Japan | 12472 (9529-15666) | 19.213 (14.678-24.132) | 6786 (5004-8757) | 13.39 (9.873-17.279) | -1.24 (-1.44 to -1.04) |
| Jordan | 3 (2-5) | 0.186 (0.119-0.275) | 16 (10-24) | 0.227 (0.145-0.353) | 1.13 (0.77 to 1.5) |
| Kazakhstan | 1189 (858-1598) | 14.468 (10.437-19.446) | 740 (553-975) | 7.905 (5.91-10.418) | -2.16 (-2.43 to -1.88) |
| Kenya | 139 (58-211) | 1.368 (0.574-2.076) | 523 (307-813) | 2.002 (1.174-3.108) | 1.48 (1.26 to 1.7) |
| Kiribati | 1 (0-1) | 1.762 (0.396-3.491) | 1 (0-2) | 1.102 (0.177-2.822) | -2.28 (-2.71 to -1.85) |
| Kuwait | 0 (0-0) | 0 (0-0) | 3 (0-7) | 0.097 (0.006-0.225) | 41.01 (32.36 to 50.23) |
| Kyrgyzstan | 134 (96-182) | 6.389 (4.592-8.707) | 160 (109-229) | 4.655 (3.182-6.666) | -1.2 (-1.46 to -0.93) |
| Lao People's Democratic Republic | 124 (56-214) | 6.691 (3.024-11.513) | 441 (269-668) | 11.021 (6.733-16.686) | 1.62 (1.33 to 1.91) |
| Latvia | 184 (130-240) | 14.306 (10.126-18.681) | 131 (95-172) | 16.572 (12.043-21.772) | -0.09 (-0.32 to 0.15) |
| Lebanon | 34 (18-53) | 2.377 (1.272-3.651) | 44 (29-65) | 1.455 (0.953-2.149) | -1.27 (-1.57 to -0.98) |
| Lesotho | 12 (6-21) | 1.855 (0.922-3.177) | 59 (31-93) | 5.911 (3.129-9.301) | 4.2 (3.74 to 4.66) |
| Liberia | 17 (10-25) | 1.583 (0.912-2.3) | 52 (25-93) | 1.867 (0.89-3.329) | 0.52 (0.03 to 1) |
| Libya | 0 (0-0) | 0.009 (0.002-0.02) | 24 (14-39) | 0.578 (0.331-0.958) | 11.74 (8.72 to 14.85) |
| Lithuania | 243 (171-318) | 13.259 (9.318-17.356) | 174 (126-229) | 15.084 (10.905-19.821) | 0.62 (0.19 to 1.05) |
| Luxembourg | 45 (36-56) | 22.672 (17.918-28.314) | 26 (19-34) | 8.082 (6.011-10.628) | -3.4 (-3.68 to -3.11) |
| Madagascar | 76 (21-125) | 1.427 (0.39-2.364) | 164 (71-281) | 1.165 (0.502-1.988) | -0.66 (-1.09 to -0.24) |
| Malawi | 23 (8-38) | 0.53 (0.192-0.859) | 105 (57-182) | 1.083 (0.593-1.88) | 2.42 (2.26 to 2.57) |
| Malaysia | 220 (131-314) | 2.441 (1.459-3.485) | 378 (215-566) | 2.132 (1.209-3.189) | -1.16 (-1.7 to -0.61) |
| Maldives | 0 (0-1) | 0.205 (0.003-0.601) | 2 (1-3) | 0.467 (0.152-0.96) | -0.1 (-1.69 to 1.51) |
| Mali | 13 (7-20) | 0.369 (0.191-0.562) | 42 (24-63) | 0.397 (0.233-0.598) | 0.51 (0.41 to 0.62) |
| Malta | 13 (9-18) | 7.013 (4.852-9.289) | 14 (10-20) | 7.344 (5.035-10.06) | -0.39 (-0.66 to -0.12) |
| Marshall Islands | 0 (0-1) | 2.402 (1.146-3.903) | 1 (1-2) | 3.848 (1.853-6.267) | 1.49 (1.38 to 1.61) |
| Mauritania | 0 (0-0) | 0 (0-0) | 0 (0-0) | 0 (0-0) | -0.14 (-0.21 to -0.07) |
| Mauritius | 18 (12-25) | 3.035 (1.966-4.197) | 53 (35-74) | 8.26 (5.534-11.499) | 2.05 (1.5 to 2.61) |
| Mexico | 1372 (1048-1738) | 3.225 (2.463-4.086) | 5630 (4259-7305) | 8.223 (6.22-10.67) | 2.93 (2.72 to 3.14) |
| Micronesia (Federated States of) | 2 (1-4) | 4.896 (2.649-7.747) | 2 (1-3) | 3.468 (1.79-5.709) | -1.46 (-1.57 to -1.34) |
| Monaco | 2 (0-4) | 14.816 (0.592-30.809) | 2 (0-5) | 17.346 (1.051-36.912) | 0.67 (0.46 to 0.88) |
| Mongolia | 24 (13-37) | 2.326 (1.292-3.568) | 139 (92-195) | 8.214 (5.472-11.562) | 5.21 (4.83 to 5.6) |
| Montenegro | 27 (19-39) | 8.573 (5.89-12.1) | 27 (19-39) | 9.183 (6.519-13.37) | 0.06 (-0.28 to 0.39) |
| Morocco | 39 (24-60) | 0.31 (0.192-0.482) | 54 (31-97) | 0.28 (0.16-0.497) | -0.26 (-0.47 to -0.04) |
| Mozambique | 5 (0-11) | 0.08 (0.002-0.191) | 56 (22-99) | 0.395 (0.155-0.692) | 5.87 (5.68 to 6.06) |
| Myanmar | 114 (35-228) | 0.559 (0.174-1.117) | 1515 (927-2357) | 5.153 (3.153-8.018) | 8.67 (8.14 to 9.21) |
| Namibia | 13 (6-21) | 2.034 (0.908-3.24) | 58 (31-92) | 4.456 (2.371-7.093) | 2.43 (2 to 2.86) |
| Nauru | 0 (0-1) | 7.99 (3.024-15.636) | 1 (0-1) | 9.703 (4.904-15.088) | 0.68 (0.5 to 0.86) |
| Nepal | 9 (0-25) | 0.103 (0.005-0.281) | 202 (83-348) | 1.215 (0.497-2.09) | 8.15 (7.34 to 8.97) |
| Netherlands | 1256 (974-1623) | 15.497 (12.018-20.018) | 819 (601-1111) | 11.063 (8.115-15.004) | -1.11 (-1.3 to -0.93) |
| New Zealand | 425 (326-539) | 23.58 (18.068-29.886) | 360 (268-468) | 14.785 (11-19.229) | -1.41 (-1.58 to -1.23) |
| Nicaragua | 30 (20-43) | 1.723 (1.175-2.487) | 121 (81-173) | 3.371 (2.267-4.807) | 2.62 (2.37 to 2.86) |
| Niger | 1 (0-4) | 0.045 (0.004-0.119) | 9 (2-21) | 0.085 (0.021-0.199) | 3.23 (2.47 to 4.01) |
| Nigeria | 510 (304-739) | 1.244 (0.74-1.802) | 1626 (958-2473) | 1.508 (0.888-2.293) | 0.81 (0.7 to 0.92) |
| Niue | 0 (0-0) | 4.639 (0.815-8.962) | 0 (0-0) | 7.089 (2.977-11.333) | 0.63 (0.34 to 0.92) |
| North Macedonia | 99 (72-131) | 9.607 (6.927-12.684) | 86 (60-123) | 7.823 (5.419-11.193) | -1.11 (-1.38 to -0.83) |
| Northern Mariana Islands | 1 (0-3) | 3.943 (0.125-9.394) | 1 (0-3) | 5.947 (0.837-11.045) | 1.28 (1.02 to 1.55) |
| Norway | 242 (171-334) | 11.15 (7.878-15.399) | 201 (146-266) | 8.065 (5.844-10.656) | -1.21 (-1.54 to -0.88) |
| Oman | 1 (1-2) | 0.105 (0.057-0.179) | 4 (2-6) | 0.124 (0.072-0.211) | 0.11 (-0.76 to 0.98) |
| Pakistan | 89 (18-171) | 0.18 (0.036-0.346) | 573 (275-946) | 0.469 (0.225-0.776) | 3.21 (2.96 to 3.45) |
| Palau | 0 (0-0) | 1.921 (0.311-4.408) | 0 (0-1) | 3.559 (0.882-6.898) | 1.99 (1.89 to 2.09) |
| Palestine | 11 (7-16) | 1.198 (0.751-1.772) | 28 (19-39) | 1.048 (0.698-1.468) | -0.7 (-0.94 to -0.47) |
| Panama | 48 (36-61) | 3.873 (2.951-4.97) | 173 (123-229) | 7.98 (5.673-10.559) | 2.55 (2.2 to 2.9) |
| Papua New Guinea | 16 (8-25) | 0.806 (0.398-1.266) | 33 (19-50) | 0.626 (0.352-0.945) | -0.64 (-1.18 to -0.1) |
| Paraguay | 83 (60-111) | 4.382 (3.196-5.854) | 262 (174-363) | 6.787 (4.516-9.399) | 1.31 (1.13 to 1.5) |
| Peru | 457 (275-654) | 4.275 (2.575-6.115) | 1163 (747-1723) | 6.015 (3.864-8.913) | 1.47 (1.17 to 1.76) |
| Philippines | 3212 (2261-4115) | 10.324 (7.268-13.228) | 8236 (6044-10664) | 13.719 (10.067-17.763) | 0.95 (0.86 to 1.04) |
| Poland | 2822 (2184-3555) | 14.882 (11.518-18.749) | 3100 (2387-3922) | 17.297 (13.318-21.886) | 0.23 (0.05 to 0.42) |
| Portugal | 962 (737-1206) | 19.233 (14.73-24.107) | 757 (566-994) | 16.519 (12.363-21.7) | -0.49 (-0.76 to -0.23) |
| Puerto Rico | 181 (132-236) | 9.804 (7.173-12.807) | 173 (126-226) | 11.789 (8.586-15.426) | 0.39 (0.14 to 0.64) |
| Qatar | 1 (1-2) | 0.497 (0.307-0.752) | 11 (7-16) | 0.513 (0.335-0.736) | 0.31 (-0.35 to 0.98) |
| Republic of Korea | 3330 (2446-4381) | 12.876 (9.458-16.941) | 2628 (1855-3599) | 10.822 (7.637-14.82) | -0.89 (-1.13 to -0.66) |
| Republic of Moldova | 414 (321-522) | 18.857 (14.624-23.762) | 290 (217-384) | 16.21 (12.101-21.445) | -0.79 (-1.02 to -0.55) |
| Romania | 1504 (1134-2010) | 13.266 (10.005-17.732) | 1690 (1227-2260) | 20.29 (14.73-27.125) | 1.21 (0.98 to 1.44) |
| Russian Federation | 11307 (9050-13995) | 15.234 (12.194-18.856) | 10617 (8428-13313) | 15.743 (12.498-19.741) | -0.41 (-0.81 to 0) |
| Rwanda | 194 (77-298) | 6.134 (2.448-9.444) | 292 (160-477) | 4.258 (2.343-6.958) | -2.61 (-3.12 to -2.1) |
| Saint Kitts and Nevis | 1 (0-1) | 5.286 (0.14-7.534) | 1 (0-3) | 3.675 (0.069-8.992) | -1.78 (-2.18 to -1.38) |
| Saint Lucia | 5 (4-6) | 7.674 (6.035-9.528) | 10 (7-13) | 10.291 (7.503-13.724) | 1.15 (0.99 to 1.31) |
| Saint Vincent and the Grenadines | 2 (2-3) | 4.576 (3.063-6.259) | 7 (5-9) | 12.052 (9.043-15.986) | 3.36 (3.12 to 3.59) |
| Samoa | 1 (1-1) | 1.244 (0.711-1.909) | 1 (1-2) | 1.301 (0.632-2.106) | 0.15 (-0.26 to 0.57) |
| San Marino | 1 (0-2) | 11.832 (0.123-19.302) | 1 (0-2) | 7.76 (0.091-14.648) | -0.23 (-0.67 to 0.21) |
| Sao Tome and Principe | 1 (0-1) | 1.844 (0.978-2.874) | 4 (2-6) | 3.347 (1.925-5.734) | 1.61 (1.23 to 2) |
| Saudi Arabia | 15 (5-27) | 0.186 (0.057-0.333) | 52 (10-106) | 0.206 (0.041-0.421) | 1.87 (1.22 to 2.52) |
| Senegal | 9 (2-18) | 0.274 (0.06-0.546) | 17 (4-35) | 0.217 (0.057-0.454) | -0.82 (-1.18 to -0.45) |
| Serbia | 655 (422-912) | 13.746 (8.853-19.137) | 490 (330-688) | 11.561 (7.79-16.231) | -1 (-1.3 to -0.71) |
| Seychelles | 2 (2-3) | 6.246 (4.187-8.877) | 7 (4-9) | 12.391 (8.275-17.273) | 1.7 (1.03 to 2.37) |
| Sierra Leone | 19 (11-30) | 1.006 (0.568-1.585) | 45 (26-72) | 1.004 (0.578-1.612) | -0.01 (-0.1 to 0.08) |
| Singapore | 79 (49-113) | 4.15 (2.568-5.949) | 90 (55-135) | 3.029 (1.833-4.522) | -2.06 (-2.71 to -1.42) |
| Slovakia | 674 (509-903) | 25.245 (19.045-33.804) | 477 (332-661) | 18.447 (12.84-25.542) | -1.19 (-1.35 to -1.04) |
| Slovenia | 160 (113-218) | 15.729 (11.127-21.481) | 57 (28-94) | 6.503 (3.241-10.727) | -3.44 (-3.67 to -3.2) |
| Solomon Islands | 1 (0-2) | 0.545 (0.171-1.167) | 5 (2-9) | 1.371 (0.582-2.542) | 4.05 (3.31 to 4.81) |
| Somalia | 0 (0-0) | 0 (0-0) | 0 (0-0) | 0 (0-0) | 0 (0 to 0) |
| South Africa | 1440 (1024-1846) | 7.632 (5.429-9.787) | 2474 (1743-3389) | 7.94 (5.596-10.88) | -0.12 (-0.35 to 0.11) |
| South Sudan | 5 (0-15) | 0.198 (0.003-0.557) | 10 (1-24) | 0.223 (0.027-0.544) | 0.79 (0.43 to 1.15) |
| Spain | 3590 (2770-4544) | 18.484 (14.265-23.398) | 2338 (1736-3032) | 11.694 (8.684-15.167) | -1.68 (-1.83 to -1.52) |
| Sri Lanka | 108 (71-154) | 1.18 (0.774-1.678) | 193 (112-297) | 1.749 (1.017-2.692) | 0.91 (0.46 to 1.36) |
| Sudan | 93 (54-153) | 1.016 (0.594-1.679) | 0 (0-1) | 0.001 (0-0.005) | -16.78 (-20.69 to -12.66) |
| Suriname | 13 (9-17) | 6.521 (4.326-8.836) | 23 (15-33) | 8.09 (5.203-11.407) | 0.89 (0.62 to 1.15) |
| Sweden | 546 (414-710) | 12.982 (9.856-16.886) | 359 (256-473) | 7.93 (5.658-10.459) | -1.2 (-1.36 to -1.04) |
| Switzerland | 481 (374-604) | 13.264 (10.314-16.655) | 225 (168-300) | 5.609 (4.188-7.482) | -2.69 (-2.91 to -2.46) |
| Syrian Arab Republic | 41 (26-63) | 0.738 (0.464-1.116) | 28 (14-49) | 0.397 (0.202-0.687) | -1.94 (-2.09 to -1.78) |
| Taiwan (Province of China) | 1600 (1242-1996) | 14.215 (11.031-17.726) | 1578 (1174-2046) | 13.876 (10.325-17.995) | -0.79 (-1.14 to -0.44) |
| Tajikistan | 81 (55-117) | 3.368 (2.276-4.844) | 107 (65-216) | 2.091 (1.272-4.197) | -2.44 (-3.28 to -1.59) |
| Thailand | 2034 (1389-2834) | 6.427 (4.387-8.953) | 5705 (3700-8122) | 17.875 (11.592-25.448) | 2.74 (2.32 to 3.17) |
| Timor-Leste | 4 (2-8) | 1.148 (0.547-2.011) | 15 (8-23) | 2.152 (1.221-3.313) | 1.59 (1.12 to 2.06) |
| Togo | 13 (6-22) | 0.815 (0.402-1.35) | 49 (24-85) | 1.19 (0.589-2.051) | 1.78 (1.45 to 2.12) |
| Tokelau | 0 (0-0) | 1.929 (0.705-3.43) | 0 (0-0) | 3.829 (1.818-6.436) | 2.01 (1.91 to 2.12) |
| Tonga | 0 (0-0) | 0.437 (0.177-0.753) | 0 (0-0) | 0.481 (0.156-0.899) | 0.07 (-0.81 to 0.97) |
| Trinidad and Tobago | 41 (30-52) | 6.556 (4.903-8.438) | 79 (54-111) | 11.496 (7.835-16.083) | 2.19 (1.91 to 2.47) |
| Tunisia | 24 (16-36) | 0.596 (0.398-0.873) | 77 (51-112) | 1.267 (0.841-1.858) | 2.21 (2.08 to 2.34) |
| Turkey | 1049 (701-1521) | 3.62 (2.42-5.249) | 1215 (839-1708) | 2.765 (1.909-3.887) | -1.36 (-1.68 to -1.04) |
| Turkmenistan | 40 (23-62) | 2.282 (1.306-3.519) | 122 (83-175) | 4.543 (3.093-6.519) | 2.39 (2.04 to 2.74) |
| Tuvalu | 0 (0-0) | 1.867 (0.772-3.433) | 0 (0-0) | 2.777 (1.337-4.841) | 0.97 (0.6 to 1.34) |
| Uganda | 224 (90-346) | 3.011 (1.205-4.659) | 971 (557-1527) | 4.83 (2.769-7.598) | 0.85 (0.46 to 1.23) |
| Ukraine | 5086 (3750-6486) | 20.385 (15.031-25.994) | 3042 (1857-4373) | 15.039 (9.182-21.622) | -1.55 (-1.94 to -1.16) |
| United Arab Emirates | 39 (21-63) | 3.351 (1.787-5.383) | 113 (66-203) | 1.651 (0.962-2.95) | -2.6 (-2.98 to -2.23) |
| United Kingdom | 4464 (3523-5600) | 15.685 (12.38-19.676) | 3893 (2969-4924) | 12.83 (9.783-16.227) | -0.74 (-0.89 to -0.6) |
| United Republic of Tanzania | 264 (116-412) | 2.334 (1.028-3.643) | 838 (497-1323) | 2.973 (1.763-4.691) | 0.86 (0.78 to 0.94) |
| United States of America | 16006 (12741-20177) | 11.923 (9.491-15.03) | 20000 (15465-25083) | 13.158 (10.174-16.502) | 0.44 (0.35 to 0.53) |
| United States Virgin Islands | 6 (0-12) | 10.229 (0.123-21.896) | 6 (0-11) | 16.555 (1.111-32.111) | 2.12 (1.94 to 2.29) |
| Uruguay | 219 (166-287) | 14.756 (11.153-19.345) | 264 (189-354) | 16.063 (11.491-21.582) | 0.4 (0.28 to 0.53) |
| Uzbekistan | 288 (177-411) | 2.942 (1.814-4.198) | 615 (425-824) | 3.447 (2.384-4.623) | 0.1 (-0.5 to 0.71) |
| Vanuatu | 2 (1-3) | 2.193 (1.09-3.59) | 5 (3-8) | 3.168 (1.714-4.982) | 1.39 (0.32 to 2.48) |
| Venezuela (Bolivarian Republic of) | 542 (427-673) | 5.656 (4.451-7.021) | 955 (597-1390) | 7.261 (4.538-10.575) | 0.93 (0.71 to 1.15) |
| Viet Nam | 83 (15-187) | 0.252 (0.045-0.571) | 5126 (3298-7622) | 9.842 (6.333-14.635) | 12.95 (11.73 to 14.17) |
| Yemen | 31 (17-54) | 0.576 (0.321-0.992) | 36 (19-65) | 0.217 (0.114-0.389) | -3.47 (-3.73 to -3.2) |
| Zambia | 104 (39-160) | 2.942 (1.103-4.524) | 616 (274-1684) | 6.381 (2.834-17.43) | 2.85 (2.56 to 3.14) |
| Zimbabwe | 121 (71-183) | 2.619 (1.544-3.96) | 424 (221-699) | 5.468 (2.849-9.019) | 2.5 (2.18 to 2.82) |

ASR, age-standardized rate; EAPC, estimated annual percentage change; CI, confidence interval.
