# Supplementary material for: Global burden of early-onset colorectal cancer related to alcohol, tobacco, and physical inactivity: evidence from the global burden of disease 2021
Source: Front Oncol. 2026 Apr 21;16:1653676. doi: 10.3389/fonc.2026.1653676 (PMC13138888; doi:10.3389/fonc.2026.1653676)
Supplement: Supplementary Table 2 — The global burden of early-onset colorectal cancer attributable to tobacco in 204 countries and territories. [file Table2.docx]

**Supplementary Table 2** The global burden of early-onset colorectal cancer attributable to tobacco in 204 countries and territories.

| **Location name** | **1990** | | **2021** | | **EAPC (95% CI)** |
| --- | --- | --- | --- | --- | --- |
|  | **Number** | **ASR** | **Number** | **ASR** |  |
| **Deaths** |  |  |  |  |  |
| Afghanistan | 1 (0-2) | 0.029 (0.007-0.057) | 6 (2-11) | 0.041 (0.017-0.077) | 2.79 (2.02 to 3.57) |
| Albania | 1 (0-1) | 0.032 (0.019-0.05) | 1 (0-1) | 0.053 (0.028-0.087) | 2.04 (1.77 to 2.31) |
| Algeria | 2 (1-4) | 0.021 (0.012-0.031) | 5 (3-8) | 0.024 (0.014-0.036) | 0.37 (0.13 to 0.62) |
| American Samoa | 0 (0-0) | 0.11 (0.063-0.174) | 0 (0-0) | 0.168 (0.094-0.262) | 1.63 (1.54 to 1.72) |
| Andorra | 0 (0-0) | 0.233 (0.136-0.369) | 0 (0-0) | 0.172 (0.088-0.28) | -0.59 (-0.87 to -0.32) |
| Angola | 1 (1-2) | 0.027 (0.014-0.046) | 4 (2-7) | 0.027 (0.014-0.044) | 0.45 (0.12 to 0.78) |
| Antigua and Barbuda | 0 (0-0) | 0.036 (0.022-0.053) | 0 (0-0) | 0.039 (0.023-0.057) | 1.41 (1.1 to 1.71) |
| Argentina | 32 (20-45) | 0.203 (0.123-0.284) | 37 (23-51) | 0.155 (0.096-0.217) | -0.86 (-1.06 to -0.66) |
| Armenia | 3 (2-4) | 0.155 (0.1-0.207) | 2 (1-2) | 0.123 (0.079-0.166) | -1.35 (-1.68 to -1.02) |
| Australia | 19 (12-26) | 0.208 (0.133-0.287) | 15 (9-22) | 0.126 (0.075-0.185) | -1.71 (-1.82 to -1.59) |
| Austria | 9 (6-12) | 0.221 (0.139-0.3) | 5 (3-6) | 0.112 (0.068-0.159) | -2.01 (-2.18 to -1.84) |
| Azerbaijan | 3 (2-5) | 0.092 (0.057-0.131) | 5 (3-7) | 0.083 (0.047-0.13) | -0.51 (-0.77 to -0.26) |
| Bahamas | 0 (0-0) | 0.069 (0.041-0.103) | 0 (0-0) | 0.101 (0.055-0.149) | 1.73 (1.52 to 1.95) |
| Bahrain | 0 (0-0) | 0.039 (0.024-0.058) | 0 (0-1) | 0.05 (0.029-0.079) | 0.1 (-0.23 to 0.43) |
| Bangladesh | 13 (7-21) | 0.026 (0.014-0.041) | 18 (9-33) | 0.02 (0.011-0.038) | -0.8 (-0.92 to -0.69) |
| Barbados | 0 (0-0) | 0.073 (0.044-0.103) | 0 (0-0) | 0.072 (0.039-0.116) | 0.28 (-0.26 to 0.82) |
| Belarus | 10 (6-14) | 0.199 (0.126-0.279) | 8 (5-12) | 0.188 (0.111-0.281) | -1.32 (-1.7 to -0.95) |
| Belgium | 11 (7-15) | 0.217 (0.135-0.301) | 5 (3-8) | 0.106 (0.064-0.153) | -2.86 (-3.2 to -2.52) |
| Belize | 0 (0-0) | 0.017 (0.01-0.024) | 0 (0-0) | 0.035 (0.021-0.052) | 2.43 (1.9 to 2.96) |
| Benin | 0 (0-0) | 0.006 (0.003-0.01) | 0 (0-0) | 0.004 (0.002-0.006) | -1.97 (-2.18 to -1.76) |
| Bermuda | 0 (0-0) | 0.126 (0.075-0.187) | 0 (0-0) | 0.121 (0.069-0.188) | 0.29 (0.12 to 0.47) |
| Bhutan | 0 (0-0) | 0.012 (0.005-0.022) | 0 (0-0) | 0.012 (0.006-0.021) | -0.57 (-0.76 to -0.37) |
| Bolivia (Plurinational State of) | 1 (0-1) | 0.03 (0.016-0.048) | 2 (1-3) | 0.027 (0.013-0.045) | -0.39 (-0.74 to -0.05) |
| Bosnia and Herzegovina | 3 (2-5) | 0.135 (0.085-0.192) | 3 (2-5) | 0.212 (0.12-0.322) | 1.75 (1.28 to 2.22) |
| Botswana | 0 (0-0) | 0.041 (0.021-0.069) | 1 (0-1) | 0.048 (0.023-0.087) | -0.14 (-0.42 to 0.14) |
| Brazil | 77 (50-108) | 0.101 (0.065-0.141) | 95 (58-138) | 0.082 (0.05-0.119) | -1.44 (-1.8 to -1.08) |
| Brunei Darussalam | 0 (0-0) | 0.181 (0.103-0.28) | 0 (0-1) | 0.146 (0.08-0.233) | -0.58 (-1.15 to 0) |
| Bulgaria | 19 (12-27) | 0.461 (0.287-0.655) | 13 (8-20) | 0.457 (0.276-0.673) | 0.06 (-0.2 to 0.31) |
| Burkina Faso | 0 (0-0) | 0.007 (0.004-0.011) | 1 (0-1) | 0.007 (0.003-0.011) | -0.04 (-0.29 to 0.2) |
| Burundi | 1 (0-1) | 0.027 (0.014-0.043) | 1 (0-2) | 0.014 (0.007-0.025) | -2.69 (-3.03 to -2.35) |
| Cabo Verde | 0 (0-0) | 0.006 (0.003-0.009) | 0 (0-0) | 0.01 (0.005-0.017) | 1.75 (1.21 to 2.29) |
| Cambodia | 4 (2-7) | 0.087 (0.045-0.144) | 10 (5-16) | 0.11 (0.056-0.183) | 0.5 (0.35 to 0.65) |
| Cameroon | 1 (0-1) | 0.013 (0.007-0.021) | 2 (1-3) | 0.011 (0.005-0.019) | -0.81 (-0.95 to -0.67) |
| Canada | 28 (17-40) | 0.191 (0.116-0.269) | 23 (14-33) | 0.135 (0.082-0.197) | -1.41 (-1.56 to -1.26) |
| Central African Republic | 0 (0-1) | 0.027 (0.013-0.045) | 1 (0-1) | 0.024 (0.01-0.045) | -0.37 (-0.5 to -0.24) |
| Chad | 0 (0-0) | 0.007 (0.004-0.011) | 1 (0-1) | 0.007 (0.004-0.012) | 0.12 (-0.13 to 0.38) |
| Chile | 8 (5-11) | 0.112 (0.071-0.151) | 13 (8-18) | 0.132 (0.082-0.185) | 1.11 (0.95 to 1.26) |
| China | 1289 (816-1821) | 0.193 (0.122-0.273) | 1614 (974-2425) | 0.243 (0.147-0.366) | 0.68 (0.51 to 0.85) |
| Colombia | 8 (5-11) | 0.046 (0.029-0.067) | 12 (7-17) | 0.045 (0.026-0.067) | -0.38 (-0.59 to -0.18) |
| Comoros | 0 (0-0) | 0.025 (0.011-0.045) | 0 (0-0) | 0.03 (0.015-0.055) | 0.53 (0.11 to 0.94) |
| Congo | 0 (0-0) | 0.025 (0.012-0.041) | 1 (0-2) | 0.032 (0.016-0.053) | 1.19 (0.83 to 1.56) |
| Cook Islands | 0 (0-0) | 0.052 (0.027-0.079) | 0 (0-0) | 0.044 (0.023-0.071) | -0.1 (-0.31 to 0.12) |
| Costa Rica | 1 (0-1) | 0.043 (0.026-0.064) | 2 (1-3) | 0.089 (0.052-0.133) | 1.99 (1.72 to 2.27) |
| Côte d'Ivoire | 0 (0-1) | 0.008 (0.005-0.013) | 1 (1-2) | 0.008 (0.004-0.014) | -0.59 (-0.88 to -0.3) |
| Croatia | 7 (4-10) | 0.286 (0.178-0.407) | 4 (3-6) | 0.238 (0.143-0.343) | -0.55 (-0.82 to -0.28) |
| Cuba | 7 (4-10) | 0.118 (0.072-0.164) | 6 (4-9) | 0.124 (0.075-0.182) | 0.25 (0.09 to 0.41) |
| Cyprus | 0 (0-1) | 0.1 (0.062-0.15) | 1 (0-1) | 0.079 (0.045-0.119) | -0.9 (-1.15 to -0.64) |
| Czechia | 22 (13-32) | 0.425 (0.256-0.605) | 10 (6-15) | 0.214 (0.125-0.324) | -3.06 (-3.48 to -2.64) |
| Democratic People's Republic of Korea | 17 (9-27) | 0.154 (0.084-0.252) | 24 (12-43) | 0.176 (0.088-0.312) | 0.67 (0.57 to 0.76) |
| Democratic Republic of the Congo | 2 (1-3) | 0.011 (0.006-0.02) | 5 (2-9) | 0.011 (0.005-0.02) | -0.02 (-0.27 to 0.23) |
| Denmark | 8 (5-11) | 0.303 (0.184-0.422) | 3 (2-4) | 0.102 (0.062-0.151) | -3.84 (-4.09 to -3.59) |
| Djibouti | 0 (0-0) | 0.038 (0.019-0.067) | 0 (0-1) | 0.054 (0.027-0.094) | 1.31 (1.07 to 1.55) |
| Dominica | 0 (0-0) | 0.033 (0.019-0.049) | 0 (0-0) | 0.05 (0.027-0.076) | 1.78 (1.52 to 2.05) |
| Dominican Republic | 1 (1-2) | 0.035 (0.022-0.053) | 3 (2-5) | 0.049 (0.027-0.077) | 1.4 (1.12 to 1.69) |
| Ecuador | 1 (1-2) | 0.021 (0.013-0.031) | 3 (1-4) | 0.028 (0.016-0.042) | 1.25 (0.69 to 1.8) |
| Egypt | 14 (9-22) | 0.053 (0.033-0.08) | 37 (20-56) | 0.068 (0.038-0.105) | 0.66 (0.54 to 0.77) |
| El Salvador | 1 (0-1) | 0.022 (0.013-0.032) | 2 (1-2) | 0.048 (0.028-0.075) | 2.92 (2.67 to 3.17) |
| Equatorial Guinea | 0 (0-0) | 0.024 (0.011-0.043) | 0 (0-0) | 0.024 (0.011-0.041) | 0.31 (-0.24 to 0.85) |
| Eritrea | 0 (0-1) | 0.03 (0.016-0.051) | 1 (0-2) | 0.031 (0.014-0.058) | -0.03 (-0.15 to 0.1) |
| Estonia | 2 (1-2) | 0.226 (0.138-0.32) | 1 (0-1) | 0.138 (0.085-0.199) | -2.43 (-2.78 to -2.08) |
| Eswatini | 0 (0-0) | 0.02 (0.01-0.033) | 0 (0-0) | 0.029 (0.013-0.05) | 1.31 (0.64 to 1.97) |
| Ethiopia | 5 (2-8) | 0.023 (0.009-0.038) | 5 (3-9) | 0.01 (0.005-0.016) | -2.77 (-3.25 to -2.3) |
| Fiji | 0 (0-0) | 0.065 (0.035-0.098) | 0 (0-0) | 0.058 (0.029-0.094) | -0.19 (-0.5 to 0.12) |
| Finland | 4 (3-6) | 0.165 (0.099-0.231) | 2 (1-2) | 0.069 (0.04-0.1) | -3.05 (-3.24 to -2.87) |
| France | 53 (33-73) | 0.181 (0.114-0.249) | 36 (21-53) | 0.127 (0.074-0.186) | -1.1 (-1.4 to -0.81) |
| Gabon | 0 (0-0) | 0.033 (0.015-0.06) | 0 (0-1) | 0.039 (0.019-0.068) | 0.42 (0.24 to 0.6) |
| Gambia | 0 (0-0) | 0.007 (0.004-0.011) | 0 (0-0) | 0.006 (0.003-0.009) | -1.1 (-1.33 to -0.88) |
| Georgia | 5 (3-6) | 0.172 (0.107-0.236) | 3 (2-4) | 0.198 (0.122-0.278) | 1.45 (1.16 to 1.74) |
| Germany | 101 (64-140) | 0.253 (0.161-0.351) | 47 (27-67) | 0.132 (0.076-0.188) | -1.73 (-1.94 to -1.52) |
| Ghana | 0 (0-0) | 0.004 (0.002-0.007) | 1 (0-2) | 0.005 (0.003-0.009) | 1.03 (0.93 to 1.12) |
| Greece | 7 (4-9) | 0.135 (0.087-0.183) | 7 (4-9) | 0.154 (0.097-0.214) | 0.43 (0.19 to 0.67) |
| Greenland | 0 (0-0) | 0.608 (0.359-0.884) | 0 (0-0) | 0.299 (0.168-0.444) | -1.9 (-2.42 to -1.37) |
| Grenada | 0 (0-0) | 0.047 (0.028-0.069) | 0 (0-0) | 0.059 (0.035-0.086) | 0.86 (0.42 to 1.3) |
| Guam | 0 (0-0) | 0.088 (0.054-0.133) | 0 (0-0) | 0.16 (0.095-0.233) | 2.06 (1.84 to 2.27) |
| Guatemala | 1 (0-1) | 0.015 (0.009-0.022) | 2 (1-3) | 0.024 (0.014-0.036) | 1.15 (0.84 to 1.45) |
| Guinea | 0 (0-0) | 0.009 (0.005-0.014) | 0 (0-1) | 0.008 (0.004-0.014) | -0.28 (-0.49 to -0.07) |
| Guinea-Bissau | 0 (0-0) | 0.008 (0.004-0.014) | 0 (0-0) | 0.01 (0.005-0.017) | 1.22 (0.91 to 1.52) |
| Guyana | 0 (0-0) | 0.045 (0.028-0.067) | 0 (0-1) | 0.081 (0.044-0.13) | 2.52 (1.99 to 3.06) |
| Haiti | 1 (1-2) | 0.049 (0.025-0.078) | 2 (1-3) | 0.029 (0.015-0.049) | -1.91 (-2.15 to -1.67) |
| Honduras | 0 (0-1) | 0.022 (0.013-0.033) | 1 (1-2) | 0.018 (0.01-0.031) | -1.07 (-1.27 to -0.87) |
| Hungary | 23 (14-32) | 0.443 (0.276-0.624) | 12 (7-18) | 0.269 (0.158-0.404) | -2.34 (-2.8 to -1.87) |
| Iceland | 0 (0-0) | 0.119 (0.075-0.165) | 0 (0-0) | 0.069 (0.041-0.102) | -2.58 (-2.84 to -2.31) |
| India | 109 (68-157) | 0.026 (0.016-0.037) | 134 (76-198) | 0.017 (0.01-0.025) | -1.33 (-1.44 to -1.22) |
| Indonesia | 60 (35-91) | 0.064 (0.037-0.096) | 169 (88-282) | 0.11 (0.058-0.184) | 1.93 (1.7 to 2.17) |
| Iran (Islamic Republic of) | 9 (5-13) | 0.034 (0.02-0.05) | 26 (15-38) | 0.055 (0.033-0.08) | 2.12 (1.83 to 2.42) |
| Iraq | 4 (2-7) | 0.05 (0.028-0.078) | 11 (6-19) | 0.05 (0.027-0.084) | 0.33 (0.13 to 0.52) |
| Ireland | 3 (2-5) | 0.193 (0.124-0.264) | 2 (1-3) | 0.079 (0.048-0.116) | -3.03 (-3.28 to -2.78) |
| Israel | 3 (2-5) | 0.135 (0.083-0.187) | 3 (2-5) | 0.076 (0.047-0.108) | -2.29 (-2.61 to -1.97) |
| Italy | 62 (40-84) | 0.216 (0.138-0.291) | 29 (18-40) | 0.118 (0.074-0.163) | -1.81 (-2.01 to -1.62) |
| Jamaica | 0 (0-1) | 0.037 (0.023-0.053) | 1 (1-2) | 0.063 (0.034-0.103) | 1 (0.33 to 1.68) |
| Japan | 170 (112-229) | 0.263 (0.173-0.353) | 73 (46-101) | 0.143 (0.091-0.199) | -2.14 (-2.35 to -1.93) |
| Jordan | 1 (1-2) | 0.078 (0.046-0.115) | 6 (4-10) | 0.09 (0.053-0.153) | 1.09 (0.86 to 1.32) |
| Kazakhstan | 13 (8-18) | 0.154 (0.099-0.215) | 9 (6-12) | 0.096 (0.06-0.134) | -1.48 (-1.83 to -1.12) |
| Kenya | 1 (1-2) | 0.01 (0.006-0.015) | 3 (2-5) | 0.013 (0.007-0.021) | 0.64 (0.39 to 0.89) |
| Kiribati | 0 (0-0) | 0.099 (0.059-0.147) | 0 (0-0) | 0.124 (0.071-0.203) | 0.42 (0.14 to 0.7) |
| Kuwait | 0 (0-0) | 0.032 (0.02-0.046) | 2 (1-3) | 0.071 (0.043-0.105) | 2.37 (1.85 to 2.89) |
| Kyrgyzstan | 2 (1-3) | 0.099 (0.063-0.137) | 3 (2-5) | 0.091 (0.054-0.134) | -0.38 (-0.56 to -0.21) |
| Lao People's Democratic Republic | 2 (1-3) | 0.108 (0.048-0.186) | 4 (2-7) | 0.11 (0.058-0.18) | -0.01 (-0.1 to 0.08) |
| Latvia | 3 (2-4) | 0.221 (0.142-0.312) | 1 (1-2) | 0.178 (0.114-0.25) | -1.05 (-1.24 to -0.87) |
| Lebanon | 1 (1-2) | 0.1 (0.05-0.158) | 3 (2-4) | 0.092 (0.052-0.14) | -0.08 (-0.32 to 0.15) |
| Lesotho | 0 (0-0) | 0.021 (0.011-0.037) | 1 (0-1) | 0.078 (0.042-0.13) | 4.52 (4.09 to 4.95) |
| Liberia | 0 (0-0) | 0.007 (0.004-0.012) | 0 (0-0) | 0.008 (0.003-0.014) | -0.17 (-0.54 to 0.19) |
| Libya | 1 (1-2) | 0.071 (0.039-0.114) | 5 (3-8) | 0.12 (0.066-0.196) | 2.84 (2.4 to 3.29) |
| Lithuania | 3 (2-4) | 0.171 (0.105-0.238) | 2 (1-3) | 0.158 (0.098-0.222) | -0.01 (-0.34 to 0.31) |
| Luxembourg | 1 (0-1) | 0.265 (0.169-0.365) | 0 (0-0) | 0.079 (0.048-0.117) | -4.08 (-4.43 to -3.72) |
| Madagascar | 1 (1-2) | 0.021 (0.012-0.033) | 2 (1-3) | 0.013 (0.006-0.021) | -1.48 (-1.59 to -1.36) |
| Malawi | 0 (0-1) | 0.007 (0.004-0.011) | 1 (1-2) | 0.011 (0.005-0.019) | 1.18 (0.96 to 1.41) |
| Malaysia | 8 (5-12) | 0.087 (0.05-0.131) | 19 (11-28) | 0.107 (0.064-0.16) | 0.32 (0.1 to 0.54) |
| Maldives | 0 (0-0) | 0.04 (0.015-0.068) | 0 (0-0) | 0.031 (0.018-0.048) | -1.24 (-1.59 to -0.88) |
| Mali | 0 (0-1) | 0.01 (0.006-0.016) | 1 (1-2) | 0.01 (0.005-0.017) | 0.2 (0.08 to 0.31) |
| Malta | 0 (0-0) | 0.143 (0.089-0.206) | 0 (0-0) | 0.112 (0.067-0.166) | -1.52 (-1.88 to -1.15) |
| Marshall Islands | 0 (0-0) | 0.059 (0.032-0.096) | 0 (0-0) | 0.097 (0.049-0.162) | 1.36 (1.21 to 1.51) |
| Mauritania | 0 (0-0) | 0.016 (0.009-0.026) | 0 (0-0) | 0.012 (0.006-0.02) | -1.06 (-1.19 to -0.92) |
| Mauritius | 0 (0-0) | 0.054 (0.033-0.074) | 1 (1-1) | 0.143 (0.093-0.204) | 2.45 (2.09 to 2.81) |
| Mexico | 14 (9-19) | 0.033 (0.021-0.045) | 30 (19-42) | 0.044 (0.028-0.061) | 0.77 (0.58 to 0.95) |
| Micronesia (Federated States of) | 0 (0-0) | 0.108 (0.058-0.174) | 0 (0-0) | 0.135 (0.066-0.217) | 0.73 (0.63 to 0.82) |
| Monaco | 0 (0-0) | 0.321 (0.181-0.493) | 0 (0-0) | 0.289 (0.152-0.486) | -0.11 (-0.33 to 0.12) |
| Mongolia | 0 (0-1) | 0.043 (0.024-0.073) | 2 (1-3) | 0.098 (0.053-0.156) | 2.8 (2.66 to 2.95) |
| Montenegro | 1 (0-1) | 0.179 (0.111-0.258) | 1 (0-1) | 0.194 (0.123-0.285) | 0.22 (-0.27 to 0.72) |
| Morocco | 4 (2-7) | 0.033 (0.018-0.053) | 6 (3-10) | 0.031 (0.016-0.051) | -0.43 (-0.51 to -0.34) |
| Mozambique | 0 (0-0) | 0.004 (0.002-0.006) | 1 (0-1) | 0.005 (0.002-0.008) | 1.87 (1.53 to 2.21) |
| Myanmar | 18 (9-31) | 0.09 (0.044-0.153) | 19 (10-29) | 0.063 (0.034-0.099) | -1.32 (-1.48 to -1.16) |
| Namibia | 0 (0-0) | 0.015 (0.008-0.024) | 0 (0-0) | 0.016 (0.008-0.026) | -0.23 (-0.67 to 0.22) |
| Nauru | 0 (0-0) | 0.206 (0.088-0.349) | 0 (0-0) | 0.18 (0.081-0.297) | -0.63 (-0.77 to -0.49) |
| Nepal | 3 (1-4) | 0.028 (0.013-0.047) | 2 (1-4) | 0.014 (0.007-0.023) | -2.64 (-2.83 to -2.45) |
| Netherlands | 22 (14-30) | 0.268 (0.172-0.372) | 10 (6-14) | 0.128 (0.079-0.186) | -2.5 (-2.72 to -2.28) |
| New Zealand | 4 (3-6) | 0.245 (0.153-0.346) | 3 (2-4) | 0.121 (0.073-0.176) | -2.37 (-2.5 to -2.25) |
| Nicaragua | 0 (0-1) | 0.021 (0.012-0.031) | 1 (1-2) | 0.03 (0.017-0.044) | 1.49 (1.28 to 1.7) |
| Niger | 0 (0-0) | 0.004 (0.002-0.007) | 0 (0-1) | 0.003 (0.001-0.005) | -1.08 (-1.19 to -0.98) |
| Nigeria | 2 (1-3) | 0.005 (0.003-0.008) | 4 (2-6) | 0.004 (0.002-0.006) | -0.79 (-0.86 to -0.72) |
| Niue | 0 (0-0) | 0.079 (0.04-0.135) | 0 (0-0) | 0.086 (0.045-0.151) | 0.04 (-0.09 to 0.16) |
| North Macedonia | 2 (1-3) | 0.185 (0.116-0.262) | 2 (1-3) | 0.179 (0.105-0.267) | -0.28 (-0.67 to 0.1) |
| Northern Mariana Islands | 0 (0-0) | 0.126 (0.066-0.204) | 0 (0-0) | 0.136 (0.081-0.205) | 1.18 (0.73 to 1.64) |
| Norway | 5 (3-8) | 0.253 (0.158-0.348) | 2 (2-3) | 0.099 (0.063-0.139) | -3.64 (-3.89 to -3.38) |
| Oman | 0 (0-0) | 0.015 (0.009-0.025) | 0 (0-1) | 0.011 (0.006-0.019) | -1.2 (-1.45 to -0.95) |
| Pakistan | 13 (8-19) | 0.026 (0.015-0.038) | 27 (15-42) | 0.022 (0.013-0.035) | -0.92 (-1.28 to -0.57) |
| Palau | 0 (0-0) | 0.04 (0.022-0.066) | 0 (0-0) | 0.054 (0.031-0.084) | 0.65 (0.53 to 0.78) |
| Palestine | 1 (0-1) | 0.08 (0.048-0.121) | 2 (1-3) | 0.073 (0.041-0.11) | -0.56 (-0.75 to -0.37) |
| Panama | 0 (0-0) | 0.026 (0.016-0.037) | 1 (0-1) | 0.034 (0.019-0.052) | 0.85 (0.57 to 1.12) |
| Papua New Guinea | 1 (0-1) | 0.027 (0.012-0.045) | 1 (1-2) | 0.027 (0.015-0.041) | -0.31 (-0.49 to -0.12) |
| Paraguay | 1 (0-1) | 0.044 (0.026-0.063) | 2 (1-3) | 0.053 (0.029-0.084) | 0.47 (0.22 to 0.71) |
| Peru | 2 (1-3) | 0.019 (0.011-0.028) | 5 (3-9) | 0.028 (0.015-0.044) | 1.08 (0.85 to 1.31) |
| Philippines | 45 (29-63) | 0.145 (0.093-0.203) | 83 (49-117) | 0.138 (0.082-0.195) | -0.4 (-0.6 to -0.2) |
| Poland | 55 (36-74) | 0.292 (0.19-0.392) | 37 (22-50) | 0.204 (0.124-0.281) | -1.7 (-1.93 to -1.47) |
| Portugal | 9 (6-13) | 0.186 (0.115-0.267) | 9 (6-12) | 0.192 (0.121-0.272) | 0.43 (0.08 to 0.77) |
| Puerto Rico | 2 (1-3) | 0.097 (0.059-0.144) | 2 (1-2) | 0.104 (0.059-0.166) | -0.11 (-0.35 to 0.13) |
| Qatar | 0 (0-0) | 0.036 (0.019-0.055) | 1 (0-1) | 0.036 (0.02-0.059) | -0.3 (-0.89 to 0.29) |
| Republic of Korea | 39 (24-57) | 0.151 (0.091-0.219) | 27 (16-41) | 0.111 (0.066-0.171) | -1.37 (-1.71 to -1.04) |
| Republic of Moldova | 4 (3-6) | 0.187 (0.119-0.26) | 4 (2-5) | 0.205 (0.125-0.291) | 0.31 (0.03 to 0.58) |
| Romania | 23 (15-32) | 0.203 (0.128-0.278) | 24 (14-34) | 0.285 (0.165-0.408) | 0.73 (0.41 to 1.04) |
| Russian Federation | 136 (89-183) | 0.183 (0.12-0.246) | 153 (99-208) | 0.227 (0.147-0.308) | 0.23 (-0.12 to 0.58) |
| Rwanda | 1 (1-2) | 0.03 (0.017-0.048) | 2 (1-3) | 0.025 (0.013-0.042) | -2.24 (-2.79 to -1.69) |
| Saint Kitts and Nevis | 0 (0-0) | 0.045 (0.027-0.066) | 0 (0-0) | 0.047 (0.025-0.074) | -0.43 (-0.8 to -0.06) |
| Saint Lucia | 0 (0-0) | 0.048 (0.029-0.069) | 0 (0-0) | 0.059 (0.034-0.09) | 0.98 (0.77 to 1.2) |
| Saint Vincent and the Grenadines | 0 (0-0) | 0.037 (0.023-0.053) | 0 (0-0) | 0.072 (0.041-0.106) | 2.46 (2.23 to 2.68) |
| Samoa | 0 (0-0) | 0.04 (0.023-0.065) | 0 (0-0) | 0.054 (0.029-0.082) | 0.94 (0.8 to 1.08) |
| San Marino | 0 (0-0) | 0.167 (0.102-0.241) | 0 (0-0) | 0.089 (0.042-0.153) | -0.98 (-1.37 to -0.59) |
| Sao Tome and Principe | 0 (0-0) | 0.008 (0.005-0.013) | 0 (0-0) | 0.014 (0.007-0.027) | 1.89 (1.64 to 2.14) |
| Saudi Arabia | 2 (1-4) | 0.026 (0.014-0.044) | 15 (8-24) | 0.06 (0.033-0.094) | 2.71 (2.57 to 2.85) |
| Senegal | 0 (0-1) | 0.014 (0.008-0.022) | 1 (0-1) | 0.01 (0.005-0.017) | -0.97 (-1.12 to -0.82) |
| Serbia | 14 (8-21) | 0.293 (0.175-0.436) | 10 (6-15) | 0.239 (0.147-0.356) | -1.14 (-1.54 to -0.74) |
| Seychelles | 0 (0-0) | 0.115 (0.071-0.172) | 0 (0-0) | 0.209 (0.126-0.32) | 1.61 (1.22 to 2) |
| Sierra Leone | 0 (0-0) | 0.011 (0.006-0.018) | 0 (0-1) | 0.008 (0.004-0.014) | -0.9 (-1.01 to -0.79) |
| Singapore | 2 (1-3) | 0.108 (0.067-0.154) | 2 (1-2) | 0.054 (0.033-0.079) | -2.73 (-3.16 to -2.3) |
| Slovakia | 9 (6-13) | 0.341 (0.208-0.495) | 6 (3-8) | 0.215 (0.127-0.322) | -1.8 (-2.02 to -1.58) |
| Slovenia | 2 (1-3) | 0.223 (0.136-0.32) | 1 (1-2) | 0.137 (0.079-0.206) | -1.7 (-1.95 to -1.45) |
| Solomon Islands | 0 (0-0) | 0.063 (0.025-0.113) | 0 (0-0) | 0.09 (0.046-0.144) | 1.45 (1.21 to 1.69) |
| Somalia | 1 (0-2) | 0.025 (0.011-0.05) | 2 (1-4) | 0.02 (0.008-0.039) | -1.69 (-2.06 to -1.31) |
| South Africa | 17 (10-24) | 0.09 (0.056-0.125) | 20 (13-30) | 0.065 (0.042-0.095) | -1.07 (-1.29 to -0.86) |
| South Sudan | 1 (0-1) | 0.02 (0.009-0.036) | 1 (0-2) | 0.024 (0.011-0.041) | 0.81 (0.46 to 1.17) |
| Spain | 48 (31-68) | 0.247 (0.161-0.348) | 29 (18-41) | 0.145 (0.088-0.205) | -1.48 (-1.95 to -1.01) |
| Sri Lanka | 3 (2-4) | 0.028 (0.017-0.042) | 2 (1-4) | 0.022 (0.011-0.038) | -1.03 (-1.33 to -0.72) |
| Sudan | 3 (1-5) | 0.031 (0.016-0.054) | 6 (3-11) | 0.027 (0.012-0.048) | -0.37 (-0.44 to -0.3) |
| Suriname | 0 (0-0) | 0.086 (0.049-0.13) | 0 (0-1) | 0.112 (0.062-0.178) | 0.98 (0.63 to 1.33) |
| Sweden | 7 (5-10) | 0.172 (0.109-0.243) | 3 (2-5) | 0.076 (0.046-0.111) | -2.57 (-2.71 to -2.42) |
| Switzerland | 6 (4-8) | 0.158 (0.098-0.22) | 3 (2-4) | 0.063 (0.04-0.093) | -3.06 (-3.31 to -2.81) |
| Syrian Arab Republic | 3 (2-5) | 0.06 (0.035-0.089) | 4 (2-6) | 0.054 (0.029-0.083) | -0.28 (-0.86 to 0.3) |
| Taiwan (Province of China) | 21 (13-30) | 0.191 (0.12-0.264) | 29 (18-42) | 0.255 (0.157-0.368) | 0.67 (0.43 to 0.91) |
| Tajikistan | 2 (1-2) | 0.064 (0.037-0.097) | 1 (1-3) | 0.029 (0.014-0.051) | -3.25 (-3.64 to -2.85) |
| Thailand | 34 (20-51) | 0.106 (0.065-0.161) | 70 (38-120) | 0.219 (0.119-0.375) | 2.08 (1.79 to 2.36) |
| Timor-Leste | 0 (0-0) | 0.046 (0.024-0.075) | 0 (0-1) | 0.049 (0.026-0.079) | 0.26 (-0.04 to 0.56) |
| Togo | 0 (0-0) | 0.009 (0.005-0.015) | 0 (0-1) | 0.011 (0.005-0.018) | 0.49 (0.34 to 0.64) |
| Tokelau | 0 (0-0) | 0.06 (0.031-0.099) | 0 (0-0) | 0.075 (0.038-0.128) | 0.44 (0.3 to 0.57) |
| Tonga | 0 (0-0) | 0.03 (0.017-0.044) | 0 (0-0) | 0.034 (0.018-0.055) | 0.62 (0.53 to 0.71) |
| Trinidad and Tobago | 1 (0-1) | 0.082 (0.051-0.118) | 1 (0-1) | 0.112 (0.063-0.171) | 0.83 (0.56 to 1.09) |
| Tunisia | 2 (1-2) | 0.038 (0.022-0.057) | 4 (2-6) | 0.06 (0.033-0.096) | 1.24 (1.05 to 1.44) |
| Turkey | 63 (36-93) | 0.217 (0.124-0.32) | 66 (39-98) | 0.15 (0.09-0.222) | -1.53 (-1.85 to -1.21) |
| Turkmenistan | 1 (1-1) | 0.058 (0.038-0.081) | 1 (1-2) | 0.049 (0.028-0.073) | -0.77 (-1.14 to -0.41) |
| Tuvalu | 0 (0-0) | 0.09 (0.048-0.146) | 0 (0-0) | 0.091 (0.051-0.142) | -0.17 (-0.32 to -0.02) |
| Uganda | 1 (1-2) | 0.015 (0.009-0.024) | 3 (2-5) | 0.016 (0.009-0.027) | -0.8 (-1.26 to -0.35) |
| Ukraine | 79 (51-110) | 0.318 (0.206-0.441) | 44 (24-69) | 0.218 (0.118-0.342) | -2.32 (-2.78 to -1.86) |
| United Arab Emirates | 1 (0-1) | 0.074 (0.036-0.127) | 3 (2-5) | 0.043 (0.024-0.077) | -2.24 (-2.55 to -1.93) |
| United Kingdom | 71 (45-95) | 0.248 (0.158-0.335) | 39 (24-53) | 0.128 (0.079-0.176) | -2.07 (-2.2 to -1.94) |
| United Republic of Tanzania | 4 (2-6) | 0.036 (0.02-0.055) | 10 (6-17) | 0.037 (0.02-0.059) | 0.02 (-0.05 to 0.09) |
| United States of America | 259 (166-350) | 0.193 (0.123-0.261) | 219 (134-305) | 0.144 (0.088-0.201) | -1.03 (-1.27 to -0.8) |
| United States Virgin Islands | 0 (0-0) | 0.115 (0.065-0.183) | 0 (0-0) | 0.09 (0.047-0.146) | -0.77 (-0.99 to -0.55) |
| Uruguay | 4 (2-5) | 0.251 (0.159-0.349) | 4 (2-5) | 0.225 (0.138-0.325) | -0.65 (-0.86 to -0.45) |
| Uzbekistan | 3 (2-4) | 0.029 (0.018-0.042) | 8 (5-11) | 0.043 (0.027-0.062) | 0.82 (0.4 to 1.24) |
| Vanuatu | 0 (0-0) | 0.047 (0.023-0.08) | 0 (0-0) | 0.048 (0.025-0.081) | -0.13 (-0.19 to -0.07) |
| Venezuela (Bolivarian Republic of) | 5 (3-7) | 0.05 (0.032-0.071) | 8 (4-13) | 0.064 (0.034-0.101) | 0.28 (0.04 to 0.52) |
| Viet Nam | 18 (10-28) | 0.054 (0.03-0.086) | 69 (38-109) | 0.133 (0.074-0.208) | 3.15 (2.98 to 3.31) |
| Yemen | 3 (1-4) | 0.048 (0.024-0.082) | 8 (4-13) | 0.045 (0.022-0.076) | -0.24 (-0.42 to -0.07) |
| Zambia | 1 (0-1) | 0.014 (0.008-0.023) | 2 (1-6) | 0.024 (0.01-0.067) | 1.95 (1.79 to 2.11) |
| Zimbabwe | 1 (1-2) | 0.027 (0.015-0.043) | 4 (2-7) | 0.056 (0.03-0.094) | 2.41 (1.85 to 2.97) |
| **Disability-adjusted life years** |  |  |  |  |  |
| Afghanistan | 53 (13-106) | 1.332 (0.337-2.654) | 283 (114-525) | 1.916 (0.774-3.551) | 2.9 (2.18 to 3.63) |
| Albania | 27 (16-42) | 1.558 (0.912-2.45) | 32 (17-52) | 2.531 (1.359-4.157) | 1.94 (1.68 to 2.2) |
| Algeria | 119 (70-180) | 1.01 (0.593-1.529) | 264 (152-401) | 1.166 (0.673-1.77) | 0.35 (0.1 to 0.61) |
| American Samoa | 1 (1-2) | 5.293 (3.029-8.232) | 2 (1-3) | 7.943 (4.42-12.326) | 1.55 (1.47 to 1.64) |
| Andorra | 4 (2-6) | 11.331 (6.598-17.977) | 3 (2-6) | 8.319 (4.309-13.575) | -0.6 (-0.87 to -0.33) |
| Angola | 58 (31-100) | 1.253 (0.659-2.153) | 188 (96-308) | 1.283 (0.657-2.103) | 0.48 (0.15 to 0.81) |
| Antigua and Barbuda | 1 (0-1) | 1.737 (1.058-2.565) | 1 (1-1) | 1.852 (1.078-2.683) | 1.35 (1.05 to 1.65) |
| Argentina | 1518 (919-2129) | 9.548 (5.783-13.392) | 1742 (1074-2430) | 7.398 (4.563-10.321) | -0.8 (-0.99 to -0.61) |
| Armenia | 131 (85-177) | 7.691 (4.96-10.353) | 87 (56-118) | 5.981 (3.838-8.122) | -1.48 (-1.82 to -1.14) |
| Australia | 900 (574-1238) | 10.019 (6.392-13.776) | 750 (442-1099) | 6.243 (3.68-9.155) | -1.6 (-1.71 to -1.49) |
| Austria | 424 (267-578) | 10.542 (6.626-14.365) | 220 (132-316) | 5.457 (3.268-7.823) | -1.98 (-2.15 to -1.81) |
| Azerbaijan | 167 (103-238) | 4.554 (2.799-6.489) | 227 (127-354) | 4.073 (2.285-6.346) | -0.68 (-0.94 to -0.42) |
| Bahamas | 5 (3-7) | 3.297 (1.962-4.892) | 10 (6-15) | 4.867 (2.67-7.19) | 1.7 (1.48 to 1.91) |
| Bahrain | 6 (4-9) | 1.9 (1.173-2.835) | 24 (14-38) | 2.464 (1.456-3.905) | 0.13 (-0.2 to 0.46) |
| Bangladesh | 605 (341-964) | 1.211 (0.683-1.93) | 842 (440-1553) | 0.957 (0.5-1.765) | -0.85 (-0.97 to -0.72) |
| Barbados | 5 (3-7) | 3.582 (2.153-4.998) | 5 (3-8) | 3.464 (1.88-5.562) | 0.19 (-0.33 to 0.71) |
| Belarus | 482 (305-673) | 9.568 (6.055-13.347) | 387 (231-577) | 9.094 (5.428-13.569) | -1.32 (-1.7 to -0.95) |
| Belgium | 523 (325-720) | 10.544 (6.551-14.52) | 261 (157-374) | 5.221 (3.152-7.487) | -2.82 (-3.14 to -2.49) |
| Belize | 1 (0-1) | 0.807 (0.488-1.144) | 4 (2-6) | 1.668 (1.008-2.474) | 2.42 (1.89 to 2.95) |
| Benin | 6 (3-10) | 0.289 (0.159-0.485) | 11 (6-18) | 0.176 (0.09-0.281) | -1.97 (-2.18 to -1.77) |
| Bermuda | 2 (1-3) | 6.013 (3.586-8.897) | 2 (1-2) | 5.961 (3.363-9.251) | 0.37 (0.2 to 0.53) |
| Bhutan | 2 (1-3) | 0.576 (0.259-1.041) | 2 (1-4) | 0.569 (0.268-0.99) | -0.56 (-0.76 to -0.37) |
| Bolivia (Plurinational State of) | 42 (22-66) | 1.394 (0.746-2.223) | 78 (40-132) | 1.253 (0.633-2.115) | -0.42 (-0.77 to -0.06) |
| Bosnia and Herzegovina | 156 (98-221) | 6.498 (4.103-9.223) | 149 (84-226) | 10.004 (5.654-15.16) | 1.66 (1.21 to 2.11) |
| Botswana | 12 (6-20) | 1.937 (1.002-3.259) | 31 (15-57) | 2.281 (1.083-4.163) | -0.13 (-0.41 to 0.15) |
| Brazil | 3743 (2391-5218) | 4.884 (3.12-6.809) | 4490 (2751-6527) | 3.872 (2.372-5.629) | -1.52 (-1.87 to -1.17) |
| Brunei Darussalam | 13 (8-20) | 8.98 (5.131-13.962) | 19 (11-30) | 7.056 (3.907-11.121) | -0.73 (-1.32 to -0.14) |
| Bulgaria | 911 (568-1293) | 21.913 (13.673-31.103) | 634 (381-936) | 21.585 (12.99-31.871) | 0.06 (-0.19 to 0.31) |
| Burkina Faso | 12 (6-20) | 0.322 (0.168-0.511) | 33 (17-53) | 0.321 (0.165-0.518) | 0 (-0.26 to 0.25) |
| Burundi | 31 (16-50) | 1.262 (0.653-2.043) | 41 (20-73) | 0.65 (0.325-1.163) | -2.71 (-3.07 to -2.36) |
| Cabo Verde | 0 (0-1) | 0.281 (0.161-0.444) | 2 (1-2) | 0.489 (0.255-0.792) | 1.6 (1.06 to 2.15) |
| Cambodia | 191 (99-314) | 4.154 (2.156-6.834) | 476 (245-786) | 5.277 (2.713-8.721) | 0.49 (0.34 to 0.65) |
| Cameroon | 27 (15-44) | 0.604 (0.332-0.977) | 78 (37-137) | 0.504 (0.24-0.891) | -0.79 (-0.92 to -0.65) |
| Canada | 1376 (835-1933) | 9.334 (5.663-13.111) | 1118 (679-1617) | 6.723 (4.085-9.721) | -1.38 (-1.53 to -1.23) |
| Central African Republic | 15 (7-26) | 1.247 (0.59-2.126) | 30 (13-56) | 1.107 (0.469-2.093) | -0.39 (-0.53 to -0.26) |
| Chad | 8 (4-13) | 0.316 (0.171-0.514) | 25 (13-42) | 0.337 (0.177-0.566) | 0.13 (-0.12 to 0.38) |
| Chile | 382 (243-512) | 5.378 (3.422-7.202) | 602 (372-843) | 6.334 (3.913-8.868) | 1.07 (0.92 to 1.22) |
| China | 62820 (39662-88682) | 9.42 (5.948-13.299) | 79091 (47723-119591) | 11.924 (7.195-18.03) | 0.65 (0.49 to 0.82) |
| Colombia | 379 (235-549) | 2.24 (1.388-3.248) | 564 (333-841) | 2.161 (1.274-3.221) | -0.4 (-0.6 to -0.2) |
| Comoros | 2 (1-4) | 1.151 (0.522-2.085) | 5 (3-10) | 1.39 (0.698-2.554) | 0.53 (0.12 to 0.93) |
| Congo | 13 (7-21) | 1.163 (0.588-1.929) | 42 (22-70) | 1.491 (0.766-2.498) | 1.2 (0.84 to 1.55) |
| Cook Islands | 0 (0-0) | 2.451 (1.293-3.742) | 0 (0-0) | 2.09 (1.105-3.416) | -0.15 (-0.35 to 0.05) |
| Costa Rica | 33 (20-48) | 2.11 (1.28-3.091) | 109 (64-163) | 4.339 (2.546-6.493) | 1.99 (1.71 to 2.27) |
| Côte d'Ivoire | 22 (12-35) | 0.393 (0.222-0.638) | 52 (25-93) | 0.379 (0.181-0.676) | -0.57 (-0.86 to -0.29) |
| Croatia | 334 (208-474) | 13.676 (8.535-19.395) | 208 (125-298) | 11.35 (6.818-16.296) | -0.56 (-0.81 to -0.31) |
| Cuba | 348 (215-483) | 5.655 (3.495-7.847) | 299 (181-438) | 5.88 (3.552-8.611) | 0.17 (0 to 0.35) |
| Cyprus | 19 (12-29) | 4.792 (2.962-7.126) | 27 (16-41) | 3.862 (2.196-5.85) | -0.79 (-1.05 to -0.53) |
| Czechia | 1036 (622-1477) | 19.901 (11.949-28.359) | 478 (279-721) | 10.137 (5.912-15.291) | -2.95 (-3.36 to -2.54) |
| Democratic People's Republic of Korea | 787 (428-1292) | 7.323 (3.988-12.024) | 1152 (580-2026) | 8.331 (4.193-14.647) | 0.6 (0.51 to 0.69) |
| Democratic Republic of the Congo | 90 (49-155) | 0.539 (0.291-0.922) | 218 (107-417) | 0.503 (0.247-0.963) | -0.04 (-0.3 to 0.22) |
| Denmark | 385 (234-537) | 14.389 (8.759-20.089) | 126 (77-185) | 4.896 (2.997-7.192) | -3.8 (-4.04 to -3.55) |
| Djibouti | 4 (2-6) | 1.778 (0.9-3.111) | 17 (9-31) | 2.524 (1.253-4.42) | 1.3 (1.06 to 1.54) |
| Dominica | 1 (0-1) | 1.561 (0.919-2.298) | 1 (0-1) | 2.381 (1.308-3.605) | 1.76 (1.5 to 2.02) |
| Dominican Republic | 61 (38-92) | 1.68 (1.04-2.527) | 138 (76-216) | 2.358 (1.302-3.679) | 1.37 (1.08 to 1.66) |
| Ecuador | 50 (31-73) | 1.011 (0.623-1.471) | 124 (71-189) | 1.315 (0.76-2.013) | 1.24 (0.68 to 1.8) |
| Egypt | 702 (421-1053) | 2.616 (1.569-3.922) | 1793 (1002-2748) | 3.355 (1.875-5.141) | 0.67 (0.54 to 0.81) |
| El Salvador | 27 (16-39) | 1.065 (0.629-1.568) | 78 (46-121) | 2.363 (1.381-3.67) | 2.96 (2.7 to 3.22) |
| Equatorial Guinea | 2 (1-4) | 1.116 (0.537-1.964) | 9 (4-16) | 1.152 (0.522-1.977) | 0.41 (-0.12 to 0.95) |
| Eritrea | 22 (11-37) | 1.421 (0.739-2.39) | 51 (23-95) | 1.489 (0.678-2.772) | 0.03 (-0.08 to 0.15) |
| Estonia | 82 (50-116) | 10.8 (6.554-15.257) | 38 (24-55) | 6.606 (4.108-9.501) | -2.41 (-2.74 to -2.07) |
| Eswatini | 3 (2-5) | 0.932 (0.488-1.531) | 8 (4-15) | 1.372 (0.607-2.37) | 1.36 (0.71 to 2.02) |
| Ethiopia | 229 (96-386) | 1.056 (0.443-1.777) | 259 (140-417) | 0.47 (0.254-0.757) | -2.72 (-3.19 to -2.24) |
| Fiji | 12 (7-18) | 3.054 (1.669-4.616) | 13 (6-21) | 2.734 (1.382-4.429) | -0.21 (-0.53 to 0.12) |
| Finland | 206 (124-288) | 7.982 (4.804-11.163) | 79 (47-114) | 3.368 (1.992-4.862) | -3.01 (-3.18 to -2.85) |
| France | 2570 (1631-3521) | 8.807 (5.589-12.065) | 1770 (1033-2598) | 6.259 (3.651-9.185) | -1.04 (-1.32 to -0.77) |
| Gabon | 7 (3-13) | 1.561 (0.7-2.841) | 17 (8-30) | 1.846 (0.89-3.202) | 0.39 (0.22 to 0.56) |
| Gambia | 1 (1-2) | 0.336 (0.184-0.532) | 3 (2-5) | 0.264 (0.142-0.424) | -1.11 (-1.34 to -0.88) |
| Georgia | 225 (140-307) | 8.382 (5.221-11.455) | 152 (94-213) | 9.487 (5.848-13.308) | 1.33 (1.04 to 1.62) |
| Germany | 4793 (3034-6658) | 12.016 (7.607-16.693) | 2291 (1325-3266) | 6.439 (3.726-9.18) | -1.73 (-1.93 to -1.53) |
| Ghana | 13 (7-21) | 0.193 (0.106-0.305) | 44 (23-73) | 0.25 (0.131-0.414) | 1.07 (0.97 to 1.17) |
| Greece | 334 (216-453) | 6.616 (4.27-8.968) | 319 (201-445) | 7.379 (4.633-10.276) | 0.36 (0.11 to 0.62) |
| Greenland | 10 (6-14) | 28.914 (17.081-42.316) | 4 (2-6) | 14.157 (7.934-20.957) | -2 (-2.51 to -1.5) |
| Grenada | 1 (1-1) | 2.255 (1.376-3.312) | 1 (1-2) | 2.78 (1.641-4.074) | 0.74 (0.32 to 1.16) |
| Guam | 3 (2-5) | 4.294 (2.631-6.47) | 6 (3-8) | 7.587 (4.55-11.166) | 1.96 (1.76 to 2.16) |
| Guatemala | 26 (16-39) | 0.74 (0.459-1.101) | 99 (59-148) | 1.182 (0.698-1.755) | 1.15 (0.84 to 1.45) |
| Guinea | 10 (6-16) | 0.403 (0.223-0.651) | 23 (12-40) | 0.38 (0.194-0.653) | -0.27 (-0.47 to -0.06) |
| Guinea-Bissau | 2 (1-3) | 0.384 (0.193-0.646) | 5 (3-8) | 0.504 (0.264-0.811) | 1.28 (0.97 to 1.6) |
| Guyana | 9 (5-13) | 2.147 (1.336-3.148) | 15 (8-24) | 3.834 (2.105-6.122) | 2.47 (1.94 to 3.01) |
| Haiti | 67 (34-107) | 2.294 (1.162-3.66) | 94 (50-157) | 1.374 (0.732-2.287) | -1.89 (-2.14 to -1.64) |
| Honduras | 22 (13-33) | 1.075 (0.629-1.615) | 47 (24-80) | 0.867 (0.446-1.482) | -1.17 (-1.38 to -0.97) |
| Hungary | 1081 (676-1519) | 21.24 (13.281-29.848) | 556 (325-836) | 12.801 (7.477-19.267) | -2.33 (-2.76 to -1.89) |
| Iceland | 8 (5-11) | 5.843 (3.674-8.089) | 6 (3-8) | 3.439 (2.069-5.078) | -2.48 (-2.74 to -2.21) |
| India | 5121 (3168-7390) | 1.218 (0.754-1.758) | 6254 (3551-9283) | 0.802 (0.455-1.191) | -1.36 (-1.48 to -1.24) |
| Indonesia | 2909 (1668-4369) | 3.078 (1.764-4.622) | 8034 (4194-13447) | 5.239 (2.735-8.769) | 1.88 (1.64 to 2.11) |
| Iran (Islamic Republic of) | 417 (248-618) | 1.634 (0.97-2.421) | 1275 (761-1859) | 2.69 (1.606-3.921) | 2.15 (1.85 to 2.46) |
| Iraq | 202 (115-315) | 2.382 (1.355-3.724) | 523 (288-880) | 2.359 (1.302-3.973) | 0.32 (0.13 to 0.5) |
| Ireland | 164 (105-225) | 9.232 (5.906-12.642) | 89 (54-131) | 3.874 (2.346-5.669) | -2.9 (-3.15 to -2.65) |
| Israel | 160 (99-221) | 6.58 (4.072-9.116) | 165 (101-234) | 3.67 (2.259-5.214) | -2.27 (-2.58 to -1.96) |
| Italy | 2983 (1899-4015) | 10.386 (6.61-13.978) | 1405 (889-1939) | 5.71 (3.614-7.88) | -1.81 (-2.01 to -1.61) |
| Jamaica | 20 (12-30) | 1.751 (1.072-2.535) | 47 (25-76) | 3.045 (1.641-4.993) | 0.99 (0.31 to 1.68) |
| Japan | 8193 (5389-11034) | 12.621 (8.301-16.997) | 3526 (2252-4897) | 6.958 (4.443-9.663) | -2.06 (-2.24 to -1.87) |
| Jordan | 67 (39-99) | 3.747 (2.191-5.581) | 302 (176-502) | 4.408 (2.57-7.342) | 1.11 (0.89 to 1.34) |
| Kazakhstan | 620 (402-868) | 7.544 (4.888-10.569) | 433 (272-602) | 4.623 (2.909-6.438) | -1.54 (-1.87 to -1.21) |
| Kenya | 50 (29-73) | 0.49 (0.283-0.724) | 162 (90-257) | 0.62 (0.344-0.981) | 0.58 (0.33 to 0.84) |
| Kiribati | 2 (1-3) | 4.716 (2.777-7.003) | 4 (2-6) | 5.969 (3.389-9.763) | 0.41 (0.13 to 0.69) |
| Kuwait | 16 (10-23) | 1.576 (0.964-2.233) | 108 (65-161) | 3.556 (2.14-5.271) | 2.39 (1.86 to 2.93) |
| Kyrgyzstan | 103 (66-143) | 4.938 (3.135-6.831) | 155 (92-227) | 4.5 (2.689-6.597) | -0.45 (-0.64 to -0.25) |
| Lao People's Democratic Republic | 95 (43-163) | 5.123 (2.31-8.755) | 211 (111-348) | 5.282 (2.777-8.686) | -0.01 (-0.1 to 0.08) |
| Latvia | 135 (86-190) | 10.474 (6.729-14.755) | 67 (43-94) | 8.452 (5.395-11.87) | -1.09 (-1.27 to -0.9) |
| Lebanon | 68 (34-108) | 4.718 (2.395-7.527) | 135 (77-206) | 4.482 (2.551-6.827) | -0.01 (-0.23 to 0.21) |
| Lesotho | 7 (4-11) | 1.007 (0.531-1.72) | 37 (20-62) | 3.733 (1.978-6.204) | 4.61 (4.2 to 5.03) |
| Liberia | 4 (2-6) | 0.341 (0.194-0.554) | 10 (5-19) | 0.366 (0.164-0.676) | -0.14 (-0.51 to 0.24) |
| Libya | 68 (38-110) | 3.425 (1.912-5.54) | 237 (132-383) | 5.747 (3.199-9.296) | 2.79 (2.36 to 3.22) |
| Lithuania | 149 (91-208) | 8.145 (4.966-11.361) | 87 (54-122) | 7.522 (4.705-10.556) | -0.05 (-0.37 to 0.27) |
| Luxembourg | 25 (16-35) | 12.774 (8.137-17.718) | 12 (8-18) | 3.868 (2.368-5.667) | -4.08 (-4.43 to -3.73) |
| Madagascar | 52 (30-83) | 0.983 (0.573-1.562) | 85 (43-139) | 0.605 (0.301-0.984) | -1.51 (-1.63 to -1.39) |
| Malawi | 14 (8-23) | 0.317 (0.181-0.532) | 48 (24-85) | 0.492 (0.244-0.881) | 1.21 (0.98 to 1.43) |
| Malaysia | 379 (220-570) | 4.208 (2.439-6.327) | 913 (545-1362) | 5.141 (3.07-7.671) | 0.28 (0.06 to 0.51) |
| Maldives | 2 (1-3) | 1.923 (0.712-3.277) | 5 (3-8) | 1.56 (0.879-2.39) | -1.1 (-1.46 to -0.73) |
| Mali | 17 (10-27) | 0.48 (0.275-0.738) | 51 (28-87) | 0.481 (0.266-0.826) | 0.23 (0.11 to 0.34) |
| Malta | 13 (8-19) | 6.929 (4.303-9.898) | 11 (6-15) | 5.447 (3.291-7.963) | -1.42 (-1.78 to -1.06) |
| Marshall Islands | 1 (0-1) | 2.855 (1.566-4.671) | 1 (1-2) | 4.664 (2.318-7.778) | 1.34 (1.2 to 1.48) |
| Mauritania | 7 (4-11) | 0.776 (0.455-1.255) | 12 (6-19) | 0.56 (0.287-0.945) | -1.08 (-1.22 to -0.95) |
| Mauritius | 16 (10-22) | 2.595 (1.609-3.562) | 44 (28-62) | 6.821 (4.447-9.73) | 2.4 (2.03 to 2.76) |
| Mexico | 679 (433-929) | 1.597 (1.017-2.184) | 1435 (901-2004) | 2.096 (1.315-2.927) | 0.71 (0.52 to 0.9) |
| Micronesia (Federated States of) | 2 (1-4) | 5.203 (2.828-8.349) | 3 (2-6) | 6.41 (3.128-10.257) | 0.67 (0.59 to 0.75) |
| Monaco | 2 (1-3) | 15.491 (8.724-23.784) | 2 (1-3) | 14.169 (7.461-23.72) | -0.08 (-0.3 to 0.15) |
| Mongolia | 21 (12-35) | 2.043 (1.139-3.429) | 79 (43-125) | 4.701 (2.536-7.437) | 2.77 (2.63 to 2.92) |
| Montenegro | 28 (17-40) | 8.659 (5.353-12.495) | 27 (17-40) | 9.273 (5.86-13.657) | 0.17 (-0.29 to 0.65) |
| Morocco | 198 (106-316) | 1.594 (0.852-2.54) | 285 (144-466) | 1.469 (0.742-2.397) | -0.51 (-0.6 to -0.43) |
| Mozambique | 10 (5-15) | 0.165 (0.094-0.262) | 32 (16-51) | 0.221 (0.11-0.357) | 1.94 (1.6 to 2.29) |
| Myanmar | 874 (430-1467) | 4.28 (2.108-7.185) | 881 (475-1373) | 2.995 (1.616-4.669) | -1.35 (-1.51 to -1.2) |
| Namibia | 5 (3-7) | 0.714 (0.396-1.111) | 10 (5-16) | 0.761 (0.394-1.231) | -0.23 (-0.68 to 0.23) |
| Nauru | 0 (0-1) | 9.835 (4.225-16.771) | 0 (0-1) | 8.618 (3.922-14.168) | -0.62 (-0.76 to -0.49) |
| Nepal | 116 (55-196) | 1.307 (0.62-2.202) | 107 (57-174) | 0.644 (0.34-1.047) | -2.67 (-2.87 to -2.48) |
| Netherlands | 1078 (687-1489) | 13.304 (8.477-18.37) | 475 (292-676) | 6.421 (3.945-9.136) | -2.47 (-2.69 to -2.25) |
| New Zealand | 211 (132-299) | 11.707 (7.329-16.585) | 144 (88-211) | 5.924 (3.621-8.665) | -2.33 (-2.46 to -2.2) |
| Nicaragua | 18 (10-26) | 1.025 (0.601-1.506) | 52 (30-78) | 1.459 (0.837-2.169) | 1.48 (1.27 to 1.7) |
| Niger | 7 (4-12) | 0.212 (0.109-0.354) | 15 (7-26) | 0.147 (0.068-0.254) | -1.11 (-1.21 to -1) |
| Nigeria | 99 (53-161) | 0.24 (0.128-0.392) | 197 (106-307) | 0.183 (0.098-0.284) | -0.75 (-0.82 to -0.68) |
| Niue | 0 (0-0) | 3.741 (1.921-6.396) | 0 (0-0) | 4.043 (2.128-7.117) | 0.01 (-0.12 to 0.14) |
| North Macedonia | 91 (57-128) | 8.796 (5.496-12.424) | 94 (55-140) | 8.499 (5.007-12.712) | -0.3 (-0.67 to 0.07) |
| Northern Mariana Islands | 2 (1-3) | 6.054 (3.194-9.871) | 2 (1-2) | 6.4 (3.854-9.611) | 1 (0.58 to 1.42) |
| Norway | 262 (163-363) | 12.109 (7.543-16.746) | 120 (76-169) | 4.825 (3.031-6.764) | -3.56 (-3.81 to -3.32) |
| Oman | 7 (4-12) | 0.738 (0.411-1.21) | 16 (9-28) | 0.524 (0.286-0.935) | -1.13 (-1.4 to -0.85) |
| Pakistan | 596 (364-876) | 1.206 (0.735-1.772) | 1281 (729-1996) | 1.05 (0.597-1.636) | -0.93 (-1.29 to -0.57) |
| Palau | 0 (0-0) | 1.938 (1.073-3.125) | 0 (0-0) | 2.533 (1.46-3.931) | 0.59 (0.47 to 0.71) |
| Palestine | 35 (21-52) | 3.919 (2.323-5.875) | 94 (53-142) | 3.541 (2.014-5.348) | -0.61 (-0.8 to -0.42) |
| Panama | 16 (9-22) | 1.263 (0.771-1.772) | 36 (20-56) | 1.685 (0.937-2.572) | 0.87 (0.61 to 1.14) |
| Papua New Guinea | 26 (12-43) | 1.32 (0.597-2.151) | 70 (39-108) | 1.308 (0.727-2.019) | -0.28 (-0.47 to -0.09) |
| Paraguay | 39 (23-57) | 2.089 (1.239-2.999) | 98 (53-154) | 2.54 (1.38-4.003) | 0.45 (0.21 to 0.69) |
| Peru | 97 (58-145) | 0.909 (0.538-1.352) | 259 (145-414) | 1.342 (0.748-2.144) | 1.12 (0.89 to 1.34) |
| Philippines | 2212 (1405-3085) | 7.112 (4.516-9.916) | 3980 (2362-5610) | 6.629 (3.935-9.344) | -0.48 (-0.67 to -0.29) |
| Poland | 2652 (1723-3560) | 13.986 (9.085-18.774) | 1717 (1047-2356) | 9.582 (5.84-13.145) | -1.73 (-1.93 to -1.52) |
| Portugal | 453 (280-647) | 9.052 (5.594-12.942) | 423 (266-599) | 9.242 (5.81-13.08) | 0.38 (0.03 to 0.74) |
| Puerto Rico | 87 (53-129) | 4.726 (2.87-7.014) | 75 (43-120) | 5.141 (2.961-8.179) | -0.04 (-0.29 to 0.2) |
| Qatar | 5 (3-8) | 1.783 (0.98-2.761) | 40 (23-64) | 1.805 (1.035-2.928) | -0.24 (-0.8 to 0.32) |
| Republic of Korea | 1910 (1161-2756) | 7.387 (4.489-10.659) | 1325 (782-2045) | 5.457 (3.218-8.419) | -1.4 (-1.72 to -1.08) |
| Republic of Moldova | 197 (125-275) | 8.982 (5.683-12.502) | 174 (107-247) | 9.737 (5.987-13.825) | 0.27 (0.02 to 0.53) |
| Romania | 1101 (691-1505) | 9.712 (6.096-13.277) | 1114 (645-1598) | 13.375 (7.741-19.182) | 0.69 (0.4 to 0.99) |
| Russian Federation | 6540 (4273-8821) | 8.811 (5.757-11.884) | 7375 (4780-10066) | 10.936 (7.088-14.926) | 0.26 (-0.06 to 0.57) |
| Rwanda | 45 (25-71) | 1.42 (0.789-2.253) | 80 (41-137) | 1.162 (0.604-2.005) | -2.23 (-2.78 to -1.66) |
| Saint Kitts and Nevis | 0 (0-1) | 2.206 (1.293-3.207) | 1 (0-1) | 2.186 (1.197-3.447) | -0.6 (-0.97 to -0.22) |
| Saint Lucia | 2 (1-2) | 2.3 (1.407-3.307) | 3 (2-4) | 2.828 (1.628-4.248) | 0.92 (0.71 to 1.14) |
| Saint Vincent and the Grenadines | 1 (1-1) | 1.794 (1.112-2.531) | 2 (1-3) | 3.366 (1.939-4.965) | 2.34 (2.13 to 2.55) |
| Samoa | 2 (1-2) | 1.919 (1.09-3.083) | 3 (1-4) | 2.532 (1.373-3.873) | 0.9 (0.76 to 1.04) |
| San Marino | 1 (1-1) | 8.137 (4.945-11.746) | 1 (0-1) | 4.316 (2.039-7.468) | -1.01 (-1.41 to -0.62) |
| Sao Tome and Principe | 0 (0-0) | 0.391 (0.212-0.631) | 1 (0-1) | 0.648 (0.315-1.234) | 1.84 (1.59 to 2.1) |
| Saudi Arabia | 104 (57-172) | 1.297 (0.712-2.142) | 737 (405-1165) | 2.914 (1.599-4.607) | 2.73 (2.59 to 2.87) |
| Senegal | 21 (12-34) | 0.656 (0.376-1.045) | 38 (19-63) | 0.491 (0.249-0.813) | -0.97 (-1.12 to -0.82) |
| Serbia | 663 (396-984) | 13.918 (8.316-20.644) | 479 (297-717) | 11.293 (7.001-16.903) | -1.13 (-1.51 to -0.75) |
| Seychelles | 2 (1-3) | 5.525 (3.402-8.22) | 5 (3-8) | 9.921 (5.991-15.018) | 1.5 (1.1 to 1.91) |
| Sierra Leone | 10 (5-16) | 0.52 (0.286-0.859) | 17 (9-29) | 0.385 (0.201-0.659) | -0.9 (-1.01 to -0.79) |
| Singapore | 100 (62-142) | 5.236 (3.25-7.469) | 78 (47-114) | 2.607 (1.59-3.821) | -2.7 (-3.14 to -2.27) |
| Slovakia | 434 (264-626) | 16.241 (9.884-23.418) | 266 (158-394) | 10.281 (6.127-15.228) | -1.76 (-1.97 to -1.56) |
| Slovenia | 109 (66-156) | 10.717 (6.528-15.349) | 59 (34-88) | 6.677 (3.859-10.056) | -1.63 (-1.85 to -1.4) |
| Solomon Islands | 5 (2-8) | 2.997 (1.2-5.351) | 15 (8-24) | 4.274 (2.18-6.832) | 1.46 (1.23 to 1.68) |
| Somalia | 40 (17-82) | 1.157 (0.5-2.339) | 92 (37-185) | 0.924 (0.37-1.859) | -1.59 (-1.97 to -1.21) |
| South Africa | 827 (510-1148) | 4.381 (2.703-6.088) | 969 (620-1411) | 3.11 (1.989-4.528) | -1.16 (-1.38 to -0.93) |
| South Sudan | 25 (11-45) | 0.913 (0.408-1.684) | 50 (23-85) | 1.119 (0.516-1.907) | 0.81 (0.46 to 1.16) |
| Spain | 2355 (1533-3286) | 12.124 (7.891-16.921) | 1403 (847-1990) | 7.021 (4.24-9.957) | -1.54 (-2 to -1.08) |
| Sri Lanka | 125 (77-184) | 1.357 (0.835-2.007) | 113 (56-198) | 1.029 (0.511-1.792) | -1.09 (-1.38 to -0.79) |
| Sudan | 136 (70-239) | 1.495 (0.773-2.621) | 290 (131-524) | 1.296 (0.584-2.346) | -0.35 (-0.42 to -0.27) |
| Suriname | 8 (5-12) | 4.129 (2.35-6.224) | 15 (8-24) | 5.323 (2.908-8.412) | 0.9 (0.55 to 1.25) |
| Sweden | 346 (217-491) | 8.234 (5.171-11.677) | 166 (102-243) | 3.668 (2.255-5.375) | -2.51 (-2.65 to -2.36) |
| Switzerland | 279 (174-389) | 7.692 (4.804-10.712) | 124 (78-181) | 3.101 (1.957-4.528) | -3.07 (-3.32 to -2.82) |
| Syrian Arab Republic | 163 (95-241) | 2.91 (1.703-4.304) | 182 (96-280) | 2.558 (1.35-3.938) | -0.37 (-0.93 to 0.2) |
| Taiwan (Province of China) | 1063 (672-1467) | 9.44 (5.97-13.029) | 1397 (865-2003) | 12.284 (7.604-17.615) | 0.57 (0.34 to 0.81) |
| Tajikistan | 77 (44-116) | 3.185 (1.846-4.823) | 73 (36-131) | 1.415 (0.704-2.556) | -3.34 (-3.76 to -2.91) |
| Thailand | 1606 (974-2436) | 5.073 (3.077-7.694) | 3349 (1832-5714) | 10.493 (5.74-17.903) | 2.03 (1.73 to 2.34) |
| Timor-Leste | 9 (4-14) | 2.228 (1.175-3.606) | 16 (9-26) | 2.329 (1.235-3.712) | 0.19 (-0.1 to 0.49) |
| Togo | 7 (4-12) | 0.44 (0.228-0.736) | 21 (10-35) | 0.497 (0.23-0.849) | 0.47 (0.33 to 0.62) |
| Tokelau | 0 (0-0) | 2.854 (1.519-4.726) | 0 (0-0) | 3.545 (1.835-5.97) | 0.42 (0.3 to 0.54) |
| Tonga | 1 (0-1) | 1.4 (0.805-2.104) | 1 (0-1) | 1.629 (0.867-2.623) | 0.62 (0.52 to 0.72) |
| Trinidad and Tobago | 24 (15-35) | 3.928 (2.433-5.627) | 37 (21-56) | 5.372 (3.001-8.155) | 0.83 (0.57 to 1.09) |
| Tunisia | 75 (44-112) | 1.82 (1.075-2.744) | 175 (96-280) | 2.893 (1.587-4.631) | 1.21 (1.03 to 1.4) |
| Turkey | 3085 (1749-4556) | 10.643 (6.035-15.718) | 3193 (1909-4727) | 7.267 (4.346-10.758) | -1.55 (-1.87 to -1.23) |
| Turkmenistan | 51 (33-71) | 2.909 (1.859-4.041) | 63 (37-94) | 2.368 (1.378-3.515) | -0.86 (-1.23 to -0.5) |
| Tuvalu | 0 (0-0) | 4.264 (2.271-6.913) | 0 (0-0) | 4.324 (2.417-6.745) | -0.16 (-0.31 to -0.02) |
| Uganda | 54 (30-84) | 0.725 (0.404-1.131) | 151 (81-253) | 0.752 (0.403-1.257) | -0.8 (-1.27 to -0.33) |
| Ukraine | 3802 (2471-5254) | 15.236 (9.904-21.059) | 2102 (1139-3293) | 10.394 (5.634-16.281) | -2.35 (-2.81 to -1.9) |
| United Arab Emirates | 43 (21-75) | 3.656 (1.745-6.325) | 144 (80-257) | 2.103 (1.163-3.74) | -2.23 (-2.52 to -1.95) |
| United Kingdom | 3378 (2149-4577) | 11.869 (7.552-16.081) | 1896 (1181-2594) | 6.249 (3.894-8.549) | -2 (-2.12 to -1.88) |
| United Republic of Tanzania | 191 (108-294) | 1.69 (0.958-2.596) | 494 (267-776) | 1.75 (0.946-2.751) | 0 (-0.07 to 0.08) |
| United States of America | 12696 (8155-17153) | 9.457 (6.075-12.778) | 10762 (6549-14995) | 7.08 (4.309-9.865) | -1.03 (-1.25 to -0.81) |
| United States Virgin Islands | 3 (2-5) | 5.457 (3.129-8.494) | 1 (1-2) | 4.333 (2.256-6.954) | -0.75 (-0.97 to -0.53) |
| Uruguay | 177 (112-245) | 11.913 (7.505-16.5) | 179 (110-256) | 10.887 (6.703-15.602) | -0.57 (-0.77 to -0.38) |
| Uzbekistan | 141 (87-199) | 1.445 (0.892-2.033) | 369 (230-538) | 2.072 (1.288-3.017) | 0.74 (0.33 to 1.17) |
| Vanuatu | 2 (1-3) | 2.268 (1.115-3.804) | 4 (2-6) | 2.294 (1.186-3.881) | -0.13 (-0.19 to -0.07) |
| Venezuela (Bolivarian Republic of) | 234 (149-329) | 2.438 (1.556-3.429) | 403 (217-635) | 3.064 (1.652-4.827) | 0.29 (0.06 to 0.52) |
| Viet Nam | 846 (474-1349) | 2.58 (1.445-4.114) | 3260 (1815-5118) | 6.261 (3.485-9.828) | 3.03 (2.88 to 3.17) |
| Yemen | 126 (62-214) | 2.318 (1.133-3.923) | 363 (180-614) | 2.172 (1.078-3.672) | -0.21 (-0.38 to -0.04) |
| Zambia | 24 (14-38) | 0.671 (0.384-1.08) | 110 (44-300) | 1.14 (0.457-3.108) | 1.96 (1.79 to 2.12) |
| Zimbabwe | 58 (33-93) | 1.266 (0.714-2.021) | 207 (108-349) | 2.678 (1.398-4.503) | 2.47 (1.91 to 3.04) |

ASR, age-standardized rate; EAPC, estimated annual percentage change; CI, confidence interval.
