# Supplementary material for: Global burden of early-onset colorectal cancer related to alcohol, tobacco, and physical inactivity: evidence from the global burden of disease 2021
Source: Front Oncol. 2026 Apr 21;16:1653676. doi: 10.3389/fonc.2026.1653676 (PMC13138888; doi:10.3389/fonc.2026.1653676)
Supplement: Supplementary Table 3 — The global burden of early-onset colorectal cancer attributable to low physical activity in 204 countries and territories. [file Table3.docx]

**Supplementary Table 3** The global burden of early-onset colorectal cancer attributable to low physical activity in 204 countries and territories.

| **Location name** | **1990** | | **2021** | | **EAPC (95% CI)** |
| --- | --- | --- | --- | --- | --- |
|  | **Number** | **ASR** | **Number** | **ASR** |  |
| **Deaths** |  |  |  |  |  |
| Afghanistan | 6 (1-12) | 0.14 (0.024-0.302) | 18 (4-37) | 0.123 (0.027-0.251) | 0.57 (-0.08 to 1.22) |
| Albania | 0 (0-0) | 0.009 (0.004-0.019) | 0 (0-0) | 0.014 (0.005-0.03) | 1.73 (1.37 to 2.09) |
| Algeria | 3 (2-5) | 0.027 (0.014-0.045) | 8 (4-14) | 0.034 (0.017-0.062) | 0.86 (0.72 to 1) |
| American Samoa | 0 (0-0) | 0.197 (0.112-0.309) | 0 (0-0) | 0.321 (0.174-0.523) | 1.82 (1.71 to 1.94) |
| Andorra | 0 (0-0) | 0.055 (0.02-0.121) | 0 (0-0) | 0.051 (0.014-0.112) | 0.23 (0 to 0.47) |
| Angola | 1 (0-1) | 0.014 (0.006-0.028) | 2 (1-4) | 0.015 (0.006-0.03) | 0.38 (0.23 to 0.52) |
| Antigua and Barbuda | 0 (0-0) | 0.035 (0.016-0.063) | 0 (0-0) | 0.046 (0.017-0.089) | 1.73 (1.52 to 1.95) |
| Argentina | 5 (2-9) | 0.029 (0.01-0.058) | 6 (2-13) | 0.026 (0.009-0.056) | -0.58 (-0.95 to -0.22) |
| Armenia | 0 (0-1) | 0.021 (0.008-0.04) | 0 (0-0) | 0.015 (0.007-0.032) | -1.19 (-1.57 to -0.81) |
| Australia | 13 (7-21) | 0.145 (0.078-0.229) | 16 (9-26) | 0.134 (0.071-0.216) | -0.21 (-0.4 to -0.02) |
| Austria | 3 (1-5) | 0.063 (0.025-0.116) | 1 (0-2) | 0.027 (0.011-0.053) | -2.51 (-2.77 to -2.25) |
| Azerbaijan | 1 (0-1) | 0.015 (0.006-0.03) | 1 (0-2) | 0.013 (0.005-0.027) | -0.4 (-0.65 to -0.15) |
| Bahamas | 0 (0-0) | 0.056 (0.025-0.102) | 0 (0-0) | 0.087 (0.033-0.167) | 1.87 (1.64 to 2.1) |
| Bahrain | 0 (0-0) | 0.044 (0.024-0.071) | 1 (0-1) | 0.057 (0.028-0.104) | 0.32 (-0.07 to 0.7) |
| Bangladesh | 5 (2-10) | 0.01 (0.004-0.02) | 11 (4-23) | 0.012 (0.005-0.026) | 1.38 (0.88 to 1.88) |
| Barbados | 0 (0-0) | 0.147 (0.081-0.224) | 0 (0-0) | 0.192 (0.102-0.319) | 1.28 (0.77 to 1.79) |
| Belarus | 2 (1-4) | 0.034 (0.014-0.072) | 2 (1-3) | 0.037 (0.015-0.075) | -0.66 (-1.03 to -0.28) |
| Belgium | 4 (2-7) | 0.074 (0.035-0.134) | 2 (1-4) | 0.046 (0.02-0.083) | -2.09 (-2.29 to -1.89) |
| Belize | 0 (0-0) | 0.014 (0.006-0.027) | 0 (0-0) | 0.031 (0.013-0.058) | 2.8 (2.31 to 3.29) |
| Benin | 0 (0-0) | 0.002 (0-0.004) | 0 (0-0) | 0.002 (0.001-0.005) | -0.02 (-0.5 to 0.45) |
| Bermuda | 0 (0-0) | 0.067 (0.03-0.122) | 0 (0-0) | 0.067 (0.028-0.129) | -0.11 (-0.22 to 0.01) |
| Bhutan | 0 (0-0) | 0.057 (0.026-0.093) | 0 (0-0) | 0.067 (0.034-0.108) | -0.09 (-0.27 to 0.1) |
| Bolivia (Plurinational State of) | 1 (0-2) | 0.023 (0.008-0.051) | 2 (1-4) | 0.025 (0.009-0.056) | -0.1 (-0.32 to 0.12) |
| Bosnia and Herzegovina | 0 (0-1) | 0.021 (0.009-0.041) | 0 (0-1) | 0.029 (0.011-0.059) | 1.36 (1.09 to 1.62) |
| Botswana | 0 (0-0) | 0.032 (0.012-0.063) | 1 (0-1) | 0.041 (0.017-0.082) | 0.92 (0.57 to 1.28) |
| Brazil | 29 (16-49) | 0.038 (0.02-0.064) | 102 (51-171) | 0.088 (0.044-0.148) | 2.76 (2.66 to 2.87) |
| Brunei Darussalam | 0 (0-0) | 0.118 (0.055-0.211) | 0 (0-1) | 0.127 (0.056-0.224) | 0.72 (0.29 to 1.14) |
| Bulgaria | 3 (1-6) | 0.076 (0.031-0.142) | 2 (1-5) | 0.082 (0.035-0.165) | 0.31 (0.03 to 0.59) |
| Burkina Faso | 0 (0-0) | 0.004 (0.002-0.009) | 0 (0-1) | 0.004 (0.001-0.009) | 0.14 (-0.22 to 0.5) |
| Burundi | 0 (0-1) | 0.01 (0.003-0.022) | 0 (0-1) | 0.007 (0.002-0.015) | -1.92 (-2.2 to -1.63) |
| Cabo Verde | 0 (0-0) | 0.005 (0.002-0.011) | 0 (0-0) | 0.009 (0.003-0.02) | 1.57 (1.11 to 2.03) |
| Cambodia | 0 (0-1) | 0.007 (0.002-0.018) | 1 (0-1) | 0.007 (0.002-0.016) | -0.77 (-1.21 to -0.33) |
| Cameroon | 1 (0-1) | 0.012 (0.005-0.023) | 2 (1-4) | 0.013 (0.005-0.028) | 0.38 (0.33 to 0.44) |
| Canada | 3 (1-6) | 0.02 (0.006-0.043) | 4 (1-8) | 0.022 (0.007-0.05) | 0.45 (0.25 to 0.65) |
| Central African Republic | 0 (0-0) | 0.019 (0.007-0.04) | 1 (0-1) | 0.02 (0.007-0.043) | -0.01 (-0.16 to 0.15) |
| Chad | 0 (0-0) | 0.006 (0.002-0.012) | 1 (0-1) | 0.008 (0.003-0.019) | 1.41 (1.24 to 1.57) |
| Chile | 1 (1-3) | 0.021 (0.008-0.043) | 3 (1-6) | 0.03 (0.011-0.061) | 1.73 (1.53 to 1.92) |
| China | 302 (155-537) | 0.045 (0.023-0.081) | 343 (160-650) | 0.052 (0.024-0.098) | 0.14 (-0.4 to 0.67) |
| Colombia | 9 (5-14) | 0.052 (0.029-0.084) | 33 (17-52) | 0.126 (0.067-0.2) | 3.64 (3.06 to 4.24) |
| Comoros | 0 (0-0) | 0.008 (0.002-0.018) | 0 (0-0) | 0.012 (0.004-0.028) | 1.94 (1.2 to 2.68) |
| Congo | 0 (0-0) | 0.02 (0.008-0.04) | 1 (0-2) | 0.026 (0.01-0.058) | 0.91 (0.46 to 1.36) |
| Cook Islands | 0 (0-0) | 0.048 (0.021-0.086) | 0 (0-0) | 0.044 (0.019-0.084) | 0.24 (-0.04 to 0.52) |
| Costa Rica | 0 (0-1) | 0.017 (0.007-0.035) | 1 (0-2) | 0.045 (0.018-0.089) | 3.21 (3 to 3.43) |
| Côte d'Ivoire | 0 (0-1) | 0.005 (0.002-0.011) | 1 (0-2) | 0.007 (0.003-0.014) | 1.21 (0.94 to 1.49) |
| Croatia | 1 (0-2) | 0.039 (0.016-0.072) | 1 (0-2) | 0.048 (0.018-0.091) | 0.97 (0.7 to 1.24) |
| Cuba | 3 (1-6) | 0.048 (0.019-0.094) | 4 (1-7) | 0.072 (0.025-0.147) | 1.61 (1.45 to 1.78) |
| Cyprus | 0 (0-0) | 0.032 (0.013-0.058) | 0 (0-0) | 0.024 (0.009-0.047) | -1.1 (-1.28 to -0.93) |
| Czechia | 3 (1-6) | 0.063 (0.027-0.115) | 3 (1-5) | 0.058 (0.026-0.113) | -0.51 (-0.94 to -0.07) |
| Democratic People's Republic of Korea | 6 (2-12) | 0.052 (0.019-0.112) | 8 (3-18) | 0.059 (0.02-0.131) | 0.68 (0.57 to 0.79) |
| Democratic Republic of the Congo | 2 (1-3) | 0.01 (0.004-0.02) | 4 (2-10) | 0.01 (0.004-0.022) | 0.2 (-0.14 to 0.55) |
| Denmark | 2 (1-3) | 0.069 (0.028-0.128) | 1 (0-2) | 0.034 (0.013-0.071) | -2.51 (-2.67 to -2.34) |
| Djibouti | 0 (0-0) | 0.009 (0.003-0.021) | 0 (0-0) | 0.014 (0.005-0.037) | 1.58 (1.44 to 1.73) |
| Dominica | 0 (0-0) | 0.018 (0.007-0.038) | 0 (0-0) | 0.027 (0.01-0.056) | 1.83 (1.64 to 2.03) |
| Dominican Republic | 1 (0-1) | 0.023 (0.011-0.041) | 2 (1-4) | 0.04 (0.016-0.075) | 2.33 (2.14 to 2.53) |
| Ecuador | 1 (0-1) | 0.011 (0.004-0.022) | 2 (1-4) | 0.022 (0.009-0.046) | 2.44 (2.12 to 2.77) |
| Egypt | 15 (8-23) | 0.056 (0.03-0.087) | 33 (16-54) | 0.061 (0.031-0.102) | 0.3 (0.2 to 0.41) |
| El Salvador | 0 (0-1) | 0.013 (0.005-0.026) | 1 (0-2) | 0.026 (0.009-0.054) | 2.37 (2.19 to 2.56) |
| Equatorial Guinea | 0 (0-0) | 0.015 (0.005-0.035) | 0 (0-0) | 0.015 (0.005-0.033) | 0.14 (-0.11 to 0.38) |
| Eritrea | 0 (0-0) | 0.005 (0.001-0.013) | 0 (0-1) | 0.006 (0.002-0.017) | 0.95 (0.56 to 1.34) |
| Estonia | 0 (0-0) | 0.025 (0.009-0.051) | 0 (0-0) | 0.022 (0.008-0.045) | -1.17 (-1.5 to -0.84) |
| Eswatini | 0 (0-0) | 0.028 (0.014-0.05) | 0 (0-1) | 0.056 (0.022-0.111) | 2.7 (2.15 to 3.24) |
| Ethiopia | 5 (1-10) | 0.022 (0.006-0.044) | 8 (3-15) | 0.015 (0.006-0.027) | -1.39 (-1.83 to -0.94) |
| Fiji | 0 (0-0) | 0.057 (0.029-0.103) | 0 (0-1) | 0.063 (0.032-0.117) | 0.54 (0.31 to 0.78) |
| Finland | 1 (0-2) | 0.045 (0.019-0.084) | 1 (0-1) | 0.023 (0.009-0.047) | -2.55 (-2.7 to -2.39) |
| France | 21 (9-36) | 0.071 (0.031-0.125) | 17 (7-30) | 0.059 (0.026-0.106) | -0.67 (-0.87 to -0.47) |
| Gabon | 0 (0-0) | 0.008 (0.002-0.018) | 0 (0-0) | 0.01 (0.003-0.022) | 0.5 (0.3 to 0.69) |
| Gambia | 0 (0-0) | 0.004 (0.001-0.007) | 0 (0-0) | 0.005 (0.002-0.01) | 0.55 (0.25 to 0.84) |
| Georgia | 1 (0-1) | 0.024 (0.01-0.044) | 0 (0-1) | 0.026 (0.01-0.051) | 0.95 (0.68 to 1.21) |
| Germany | 21 (8-43) | 0.052 (0.021-0.108) | 11 (4-22) | 0.031 (0.012-0.063) | -1.38 (-1.66 to -1.09) |
| Ghana | 1 (0-2) | 0.015 (0.006-0.028) | 3 (1-7) | 0.019 (0.007-0.039) | 0.86 (0.19 to 1.53) |
| Greece | 1 (0-2) | 0.022 (0.008-0.044) | 1 (0-2) | 0.026 (0.009-0.053) | 0.59 (0.21 to 0.97) |
| Greenland | 0 (0-0) | 0.078 (0.026-0.177) | 0 (0-0) | 0.051 (0.017-0.115) | -1.03 (-1.54 to -0.51) |
| Grenada | 0 (0-0) | 0.042 (0.019-0.075) | 0 (0-0) | 0.056 (0.026-0.098) | 1.28 (1.06 to 1.51) |
| Guam | 0 (0-0) | 0.015 (0.007-0.029) | 0 (0-0) | 0.038 (0.017-0.074) | 3.39 (2.87 to 3.9) |
| Guatemala | 0 (0-0) | 0.003 (0.001-0.008) | 1 (0-1) | 0.007 (0.002-0.017) | 2.54 (1.92 to 3.17) |
| Guinea | 0 (0-0) | 0.004 (0.001-0.009) | 0 (0-1) | 0.004 (0.001-0.008) | -0.42 (-0.61 to -0.23) |
| Guinea-Bissau | 0 (0-0) | 0.016 (0.005-0.032) | 0 (0-0) | 0.019 (0.007-0.039) | 0.6 (0.47 to 0.72) |
| Guyana | 0 (0-0) | 0.025 (0.01-0.047) | 0 (0-0) | 0.053 (0.019-0.11) | 2.71 (2.18 to 3.25) |
| Haiti | 1 (0-3) | 0.048 (0.014-0.097) | 3 (1-7) | 0.048 (0.016-0.098) | 0.09 (0 to 0.18) |
| Honduras | 0 (0-1) | 0.013 (0.005-0.025) | 1 (0-2) | 0.015 (0.005-0.034) | 0.22 (0.02 to 0.41) |
| Hungary | 3 (1-6) | 0.059 (0.026-0.114) | 3 (1-5) | 0.063 (0.025-0.122) | -0.14 (-0.35 to 0.07) |
| Iceland | 0 (0-0) | 0.04 (0.015-0.081) | 0 (0-0) | 0.033 (0.013-0.064) | -1.34 (-1.58 to -1.09) |
| India | 69 (35-113) | 0.016 (0.008-0.027) | 118 (60-194) | 0.015 (0.008-0.025) | -0.72 (-0.96 to -0.48) |
| Indonesia | 50 (24-86) | 0.053 (0.026-0.091) | 116 (55-212) | 0.076 (0.036-0.138) | 1.41 (1.34 to 1.48) |
| Iran (Islamic Republic of) | 12 (6-18) | 0.045 (0.023-0.072) | 29 (16-43) | 0.061 (0.034-0.091) | 1.32 (0.9 to 1.73) |
| Iraq | 5 (3-9) | 0.062 (0.032-0.102) | 16 (8-28) | 0.072 (0.034-0.126) | 0.76 (0.63 to 0.9) |
| Ireland | 1 (1-2) | 0.071 (0.032-0.13) | 1 (1-2) | 0.05 (0.022-0.091) | -1.23 (-1.41 to -1.05) |
| Israel | 1 (0-2) | 0.044 (0.017-0.082) | 1 (1-3) | 0.033 (0.013-0.067) | -1.23 (-1.55 to -0.9) |
| Italy | 27 (14-44) | 0.095 (0.049-0.153) | 18 (8-30) | 0.074 (0.033-0.123) | -0.72 (-0.88 to -0.56) |
| Jamaica | 1 (0-1) | 0.048 (0.026-0.075) | 2 (1-3) | 0.112 (0.057-0.186) | 3 (2.43 to 3.57) |
| Japan | 134 (76-206) | 0.207 (0.117-0.318) | 82 (43-137) | 0.163 (0.086-0.27) | -0.78 (-1.07 to -0.49) |
| Jordan | 1 (0-1) | 0.044 (0.021-0.075) | 3 (1-5) | 0.039 (0.017-0.074) | -0.24 (-0.36 to -0.12) |
| Kazakhstan | 2 (1-4) | 0.028 (0.013-0.051) | 2 (1-4) | 0.024 (0.009-0.047) | -0.4 (-0.61 to -0.18) |
| Kenya | 2 (1-3) | 0.018 (0.008-0.03) | 7 (3-12) | 0.027 (0.013-0.045) | 1.66 (1.53 to 1.79) |
| Kiribati | 0 (0-0) | 0.093 (0.049-0.15) | 0 (0-0) | 0.105 (0.055-0.183) | 0.22 (0.04 to 0.4) |
| Kuwait | 1 (0-1) | 0.063 (0.036-0.092) | 4 (2-7) | 0.142 (0.082-0.218) | 1.76 (1.08 to 2.46) |
| Kyrgyzstan | 0 (0-1) | 0.017 (0.006-0.034) | 0 (0-1) | 0.013 (0.005-0.028) | -0.92 (-1.08 to -0.76) |
| Lao People's Democratic Republic | 0 (0-1) | 0.014 (0.004-0.033) | 1 (0-1) | 0.013 (0.004-0.031) | -0.43 (-0.91 to 0.05) |
| Latvia | 1 (0-1) | 0.061 (0.026-0.11) | 0 (0-1) | 0.049 (0.02-0.099) | -0.9 (-1.13 to -0.68) |
| Lebanon | 1 (1-2) | 0.093 (0.043-0.167) | 2 (1-4) | 0.073 (0.037-0.123) | -0.65 (-0.89 to -0.42) |
| Lesotho | 0 (0-0) | 0.004 (0.001-0.009) | 0 (0-0) | 0.007 (0.002-0.017) | 2.44 (1.94 to 2.95) |
| Liberia | 0 (0-0) | 0.019 (0.009-0.033) | 1 (0-2) | 0.03 (0.012-0.057) | 1.04 (0.64 to 1.44) |
| Libya | 2 (1-3) | 0.078 (0.037-0.135) | 6 (3-10) | 0.136 (0.061-0.241) | 2.48 (2.22 to 2.74) |
| Lithuania | 1 (0-2) | 0.049 (0.023-0.09) | 1 (0-1) | 0.051 (0.02-0.097) | 0.4 (0.07 to 0.73) |
| Luxembourg | 0 (0-0) | 0.067 (0.03-0.12) | 0 (0-0) | 0.028 (0.011-0.055) | -2.79 (-2.96 to -2.63) |
| Madagascar | 1 (0-1) | 0.01 (0.003-0.024) | 1 (0-3) | 0.009 (0.003-0.022) | 0.03 (-0.09 to 0.15) |
| Malawi | 0 (0-0) | 0.003 (0.001-0.006) | 0 (0-1) | 0.003 (0.001-0.007) | -0.02 (-0.34 to 0.3) |
| Malaysia | 5 (2-8) | 0.052 (0.025-0.092) | 16 (7-27) | 0.091 (0.038-0.151) | 2.14 (1.7 to 2.59) |
| Maldives | 0 (0-0) | 0.085 (0.025-0.15) | 0 (0-0) | 0.04 (0.021-0.067) | -2.6 (-2.77 to -2.43) |
| Mali | 0 (0-1) | 0.013 (0.006-0.025) | 1 (1-3) | 0.012 (0.005-0.025) | -0.16 (-0.41 to 0.1) |
| Malta | 0 (0-0) | 0.078 (0.039-0.138) | 0 (0-0) | 0.074 (0.033-0.127) | -1.01 (-1.3 to -0.71) |
| Marshall Islands | 0 (0-0) | 0.183 (0.097-0.282) | 0 (0-0) | 0.292 (0.147-0.472) | 1.29 (1.1 to 1.47) |
| Mauritania | 0 (0-0) | 0.029 (0.013-0.049) | 1 (0-1) | 0.034 (0.018-0.061) | 0.61 (0.49 to 0.73) |
| Mauritius | 0 (0-0) | 0.014 (0.006-0.028) | 0 (0-1) | 0.04 (0.017-0.078) | 3.07 (2.7 to 3.44) |
| Mexico | 5 (2-9) | 0.011 (0.005-0.02) | 24 (11-44) | 0.035 (0.015-0.064) | 4.08 (3.37 to 4.79) |
| Micronesia (Federated States of) | 0 (0-0) | 0.207 (0.109-0.34) | 0 (0-0) | 0.252 (0.13-0.414) | 0.62 (0.52 to 0.71) |
| Monaco | 0 (0-0) | 0.095 (0.033-0.189) | 0 (0-0) | 0.101 (0.035-0.213) | 0.45 (0.27 to 0.62) |
| Mongolia | 0 (0-0) | 0.009 (0.003-0.019) | 0 (0-0) | 0.012 (0.004-0.027) | 0.33 (0.01 to 0.65) |
| Montenegro | 0 (0-0) | 0.028 (0.011-0.057) | 0 (0-0) | 0.031 (0.011-0.062) | 0.43 (-0.05 to 0.9) |
| Morocco | 6 (3-12) | 0.049 (0.022-0.094) | 16 (7-29) | 0.083 (0.034-0.149) | 1.87 (1.73 to 2.01) |
| Mozambique | 0 (0-0) | 0.002 (0.001-0.004) | 0 (0-1) | 0.002 (0.001-0.004) | 0.88 (0.63 to 1.12) |
| Myanmar | 1 (0-4) | 0.007 (0.002-0.017) | 2 (1-6) | 0.008 (0.002-0.02) | 0.7 (0.11 to 1.29) |
| Namibia | 0 (0-0) | 0.018 (0.008-0.033) | 0 (0-1) | 0.026 (0.012-0.052) | 0.96 (0.68 to 1.25) |
| Nauru | 0 (0-0) | 0.131 (0.045-0.252) | 0 (0-0) | 0.124 (0.047-0.245) | -0.37 (-0.48 to -0.26) |
| Nepal | 1 (0-2) | 0.008 (0.003-0.018) | 1 (0-3) | 0.009 (0.003-0.021) | 0.65 (0 to 1.3) |
| Netherlands | 3 (1-5) | 0.033 (0.013-0.066) | 2 (1-4) | 0.026 (0.009-0.057) | -0.88 (-1.19 to -0.58) |
| New Zealand | 1 (1-2) | 0.067 (0.028-0.13) | 2 (1-3) | 0.077 (0.036-0.137) | 1.49 (1.03 to 1.95) |
| Nicaragua | 0 (0-0) | 0.01 (0.004-0.019) | 1 (0-1) | 0.015 (0.006-0.03) | 1.67 (1.47 to 1.87) |
| Niger | 0 (0-1) | 0.008 (0.003-0.017) | 1 (0-2) | 0.007 (0.003-0.016) | -0.19 (-0.32 to -0.06) |
| Nigeria | 5 (2-8) | 0.011 (0.005-0.021) | 13 (6-24) | 0.012 (0.005-0.022) | 0.52 (0.39 to 0.66) |
| Niue | 0 (0-0) | 0.075 (0.031-0.142) | 0 (0-0) | 0.088 (0.041-0.166) | 0.18 (0.07 to 0.29) |
| North Macedonia | 0 (0-1) | 0.033 (0.013-0.063) | 0 (0-1) | 0.031 (0.012-0.064) | -0.32 (-0.7 to 0.07) |
| Northern Mariana Islands | 0 (0-0) | 0.089 (0.041-0.159) | 0 (0-0) | 0.111 (0.051-0.198) | 1.05 (0.72 to 1.38) |
| Norway | 2 (1-3) | 0.076 (0.036-0.137) | 1 (1-2) | 0.048 (0.021-0.086) | -1.69 (-1.84 to -1.53) |
| Oman | 0 (0-0) | 0.017 (0.008-0.031) | 0 (0-1) | 0.014 (0.006-0.025) | -0.67 (-0.86 to -0.48) |
| Pakistan | 8 (3-13) | 0.016 (0.007-0.027) | 26 (12-47) | 0.021 (0.01-0.039) | 0.78 (0.67 to 0.88) |
| Palau | 0 (0-0) | 0.114 (0.055-0.205) | 0 (0-0) | 0.127 (0.058-0.231) | 0.23 (0.12 to 0.34) |
| Palestine | 1 (1-2) | 0.128 (0.064-0.22) | 3 (1-4) | 0.099 (0.052-0.15) | -0.75 (-0.85 to -0.64) |
| Panama | 0 (0-0) | 0.017 (0.006-0.035) | 1 (0-1) | 0.031 (0.01-0.059) | 2.56 (2.31 to 2.8) |
| Papua New Guinea | 1 (0-1) | 0.033 (0.014-0.06) | 2 (1-3) | 0.032 (0.015-0.06) | -0.23 (-0.36 to -0.1) |
| Paraguay | 0 (0-0) | 0.008 (0.003-0.018) | 1 (0-1) | 0.015 (0.005-0.033) | 2.19 (1.99 to 2.39) |
| Peru | 2 (1-3) | 0.016 (0.006-0.031) | 4 (1-9) | 0.02 (0.007-0.045) | 0.83 (0.64 to 1.01) |
| Philippines | 5 (2-10) | 0.018 (0.008-0.032) | 17 (7-31) | 0.028 (0.012-0.052) | 1.84 (1.61 to 2.08) |
| Poland | 12 (6-21) | 0.065 (0.033-0.11) | 12 (6-22) | 0.067 (0.031-0.125) | -0.52 (-0.74 to -0.29) |
| Portugal | 4 (2-6) | 0.071 (0.032-0.126) | 4 (2-8) | 0.087 (0.034-0.169) | 0.92 (0.75 to 1.09) |
| Puerto Rico | 1 (0-1) | 0.043 (0.017-0.08) | 1 (0-2) | 0.055 (0.025-0.103) | 0.56 (0.37 to 0.75) |
| Qatar | 0 (0-0) | 0.066 (0.035-0.107) | 1 (1-2) | 0.05 (0.025-0.086) | -1.65 (-2.11 to -1.19) |
| Republic of Korea | 19 (9-32) | 0.074 (0.035-0.125) | 18 (8-34) | 0.075 (0.033-0.14) | -0.09 (-0.34 to 0.16) |
| Republic of Moldova | 1 (0-1) | 0.033 (0.013-0.063) | 1 (0-1) | 0.031 (0.011-0.06) | -0.32 (-0.61 to -0.03) |
| Romania | 6 (3-11) | 0.05 (0.023-0.094) | 6 (3-11) | 0.074 (0.033-0.136) | 0.88 (0.56 to 1.2) |
| Russian Federation | 23 (10-42) | 0.031 (0.013-0.056) | 23 (10-42) | 0.034 (0.015-0.063) | -0.32 (-0.59 to -0.05) |
| Rwanda | 0 (0-1) | 0.012 (0.004-0.027) | 1 (0-1) | 0.008 (0.002-0.018) | -2.46 (-2.98 to -1.94) |
| Saint Kitts and Nevis | 0 (0-0) | 0.043 (0.019-0.074) | 0 (0-0) | 0.041 (0.015-0.09) | -0.29 (-0.47 to -0.11) |
| Saint Lucia | 0 (0-0) | 0.03 (0.012-0.06) | 0 (0-0) | 0.045 (0.018-0.091) | 1.81 (1.58 to 2.05) |
| Saint Vincent and the Grenadines | 0 (0-0) | 0.028 (0.012-0.05) | 0 (0-0) | 0.061 (0.026-0.12) | 2.75 (2.53 to 2.97) |
| Samoa | 0 (0-0) | 0.087 (0.047-0.139) | 0 (0-0) | 0.129 (0.062-0.213) | 1.5 (1.4 to 1.6) |
| San Marino | 0 (0-0) | 0.048 (0.018-0.093) | 0 (0-0) | 0.04 (0.014-0.087) | 0.76 (0.25 to 1.27) |
| Sao Tome and Principe | 0 (0-0) | 0.005 (0.002-0.01) | 0 (0-0) | 0.007 (0.002-0.016) | 0.92 (0.73 to 1.11) |
| Saudi Arabia | 3 (1-5) | 0.034 (0.016-0.065) | 20 (10-34) | 0.079 (0.041-0.136) | 3.18 (3 to 3.36) |
| Senegal | 0 (0-1) | 0.009 (0.004-0.018) | 1 (0-2) | 0.012 (0.005-0.024) | 1.21 (0.91 to 1.52) |
| Serbia | 2 (1-5) | 0.051 (0.021-0.097) | 2 (1-4) | 0.042 (0.015-0.085) | -1.08 (-1.42 to -0.74) |
| Seychelles | 0 (0-0) | 0.032 (0.012-0.064) | 0 (0-0) | 0.058 (0.021-0.127) | 2.47 (2.16 to 2.77) |
| Sierra Leone | 0 (0-0) | 0.007 (0.003-0.013) | 0 (0-1) | 0.01 (0.004-0.02) | 1.39 (1.29 to 1.5) |
| Singapore | 2 (1-3) | 0.094 (0.046-0.158) | 2 (1-3) | 0.061 (0.03-0.106) | -2.05 (-2.53 to -1.57) |
| Slovakia | 1 (1-3) | 0.052 (0.022-0.098) | 1 (0-2) | 0.046 (0.017-0.091) | -0.52 (-0.7 to -0.34) |
| Slovenia | 0 (0-1) | 0.042 (0.017-0.078) | 0 (0-1) | 0.031 (0.012-0.061) | -1.33 (-1.54 to -1.12) |
| Solomon Islands | 0 (0-0) | 0.058 (0.023-0.118) | 0 (0-1) | 0.092 (0.041-0.168) | 1.72 (1.54 to 1.9) |
| Somalia | 0 (0-1) | 0.012 (0.003-0.029) | 1 (0-2) | 0.01 (0.002-0.022) | -1.37 (-1.61 to -1.13) |
| South Africa | 17 (10-26) | 0.091 (0.051-0.137) | 33 (18-51) | 0.105 (0.059-0.163) | 0.66 (0.38 to 0.94) |
| South Sudan | 0 (0-1) | 0.008 (0.003-0.019) | 1 (0-1) | 0.012 (0.004-0.027) | 1.43 (0.95 to 1.91) |
| Spain | 12 (6-22) | 0.061 (0.029-0.115) | 11 (4-20) | 0.053 (0.022-0.1) | -0.36 (-0.5 to -0.22) |
| Sri Lanka | 1 (0-1) | 0.006 (0.002-0.012) | 1 (0-2) | 0.008 (0.002-0.016) | 1.75 (1.39 to 2.1) |
| Sudan | 15 (7-27) | 0.17 (0.082-0.299) | 39 (19-68) | 0.177 (0.084-0.302) | 0.33 (0.24 to 0.41) |
| Suriname | 0 (0-0) | 0.07 (0.032-0.12) | 0 (0-1) | 0.117 (0.058-0.193) | 1.9 (1.68 to 2.12) |
| Sweden | 2 (1-4) | 0.051 (0.021-0.098) | 2 (1-3) | 0.041 (0.019-0.073) | 0.13 (-0.16 to 0.42) |
| Switzerland | 2 (1-3) | 0.045 (0.018-0.085) | 1 (0-2) | 0.027 (0.011-0.051) | -1.58 (-1.83 to -1.34) |
| Syrian Arab Republic | 3 (1-5) | 0.054 (0.024-0.09) | 4 (2-8) | 0.063 (0.029-0.112) | 0.7 (0.09 to 1.33) |
| Taiwan (Province of China) | 17 (9-27) | 0.148 (0.08-0.243) | 27 (14-47) | 0.24 (0.119-0.409) | 1.34 (1.13 to 1.55) |
| Tajikistan | 0 (0-1) | 0.011 (0.005-0.022) | 0 (0-1) | 0.008 (0.003-0.018) | -1.38 (-1.68 to -1.08) |
| Thailand | 7 (3-15) | 0.023 (0.009-0.048) | 17 (6-36) | 0.052 (0.018-0.112) | 2.37 (2.01 to 2.73) |
| Timor-Leste | 0 (0-0) | 0.011 (0.003-0.025) | 0 (0-0) | 0.01 (0.004-0.021) | -0.88 (-1.32 to -0.44) |
| Togo | 0 (0-0) | 0.003 (0.001-0.006) | 0 (0-0) | 0.004 (0.001-0.009) | 0.96 (0.66 to 1.26) |
| Tokelau | 0 (0-0) | 0.074 (0.033-0.141) | 0 (0-0) | 0.086 (0.042-0.147) | 0.12 (-0.05 to 0.3) |
| Tonga | 0 (0-0) | 0.053 (0.027-0.088) | 0 (0-0) | 0.058 (0.03-0.103) | 0.32 (0.25 to 0.38) |
| Trinidad and Tobago | 1 (0-1) | 0.081 (0.042-0.13) | 1 (0-2) | 0.135 (0.069-0.236) | 1.55 (1.36 to 1.74) |
| Tunisia | 0 (0-1) | 0.01 (0.004-0.019) | 1 (0-2) | 0.019 (0.007-0.036) | 2.03 (1.87 to 2.19) |
| Turkey | 22 (10-40) | 0.074 (0.034-0.137) | 26 (12-47) | 0.059 (0.027-0.106) | -0.76 (-1.26 to -0.26) |
| Turkmenistan | 0 (0-0) | 0.009 (0.004-0.017) | 0 (0-1) | 0.012 (0.004-0.023) | 0.53 (0.06 to 1) |
| Tuvalu | 0 (0-0) | 0.095 (0.041-0.174) | 0 (0-0) | 0.076 (0.037-0.135) | -1.01 (-1.17 to -0.86) |
| Uganda | 1 (0-1) | 0.007 (0.003-0.017) | 2 (1-4) | 0.009 (0.003-0.02) | -0.08 (-0.42 to 0.26) |
| Ukraine | 9 (4-19) | 0.037 (0.015-0.076) | 6 (2-13) | 0.03 (0.01-0.062) | -1.26 (-1.54 to -0.99) |
| United Arab Emirates | 1 (0-2) | 0.082 (0.035-0.149) | 4 (2-8) | 0.066 (0.028-0.115) | -1.15 (-1.59 to -0.71) |
| United Kingdom | 52 (30-75) | 0.183 (0.106-0.263) | 46 (26-67) | 0.152 (0.086-0.221) | -0.52 (-0.74 to -0.3) |
| United Republic of Tanzania | 0 (0-1) | 0.003 (0.001-0.008) | 1 (0-2) | 0.003 (0.001-0.006) | -0.9 (-1.22 to -0.58) |
| United States of America | 51 (23-89) | 0.038 (0.017-0.066) | 74 (35-123) | 0.049 (0.023-0.081) | 0.95 (0.59 to 1.31) |
| United States Virgin Islands | 0 (0-0) | 0.044 (0.017-0.093) | 0 (0-0) | 0.034 (0.011-0.074) | 0.21 (-0.15 to 0.58) |
| Uruguay | 1 (0-2) | 0.067 (0.03-0.129) | 1 (1-3) | 0.088 (0.037-0.169) | 1.2 (1 to 1.4) |
| Uzbekistan | 1 (0-1) | 0.006 (0.002-0.011) | 1 (1-3) | 0.008 (0.003-0.015) | 1.42 (1.06 to 1.78) |
| Vanuatu | 0 (0-0) | 0.006 (0.002-0.016) | 0 (0-0) | 0.006 (0.002-0.017) | -0.96 (-1.44 to -0.49) |
| Venezuela (Bolivarian Republic of) | 2 (1-3) | 0.018 (0.007-0.033) | 5 (2-10) | 0.035 (0.013-0.072) | 2.08 (1.88 to 2.28) |
| Viet Nam | 4 (2-8) | 0.012 (0.005-0.025) | 18 (7-39) | 0.034 (0.013-0.075) | 4.24 (3.91 to 4.57) |
| Yemen | 2 (1-5) | 0.044 (0.019-0.089) | 8 (3-16) | 0.05 (0.021-0.093) | 0.37 (0.23 to 0.51) |
| Zambia | 0 (0-1) | 0.013 (0.006-0.026) | 2 (1-5) | 0.018 (0.005-0.049) | 1.02 (0.55 to 1.51) |
| Zimbabwe | 0 (0-1) | 0.01 (0.004-0.021) | 2 (1-4) | 0.026 (0.009-0.055) | 3.86 (3.18 to 4.54) |
| **Disability-adjusted life years** |  |  |  |  |  |
| Afghanistan | 264 (46-564) | 6.608 (1.144-14.092) | 875 (197-1773) | 5.923 (1.332-11.996) | 0.59 (0.04 to 1.15) |
| Albania | 8 (3-15) | 0.454 (0.185-0.886) | 8 (3-18) | 0.653 (0.25-1.421) | 1.63 (1.28 to 1.99) |
| Algeria | 158 (83-260) | 1.344 (0.705-2.208) | 384 (186-685) | 1.695 (0.821-3.025) | 0.83 (0.68 to 0.97) |
| American Samoa | 2 (1-4) | 9.822 (5.555-15.127) | 4 (2-6) | 15.565 (8.49-25.134) | 1.7 (1.6 to 1.81) |
| Andorra | 1 (0-2) | 2.669 (0.989-5.848) | 1 (0-2) | 2.485 (0.725-5.342) | 0.23 (0 to 0.46) |
| Angola | 31 (13-62) | 0.675 (0.284-1.34) | 104 (42-207) | 0.711 (0.288-1.414) | 0.41 (0.26 to 0.55) |
| Antigua and Barbuda | 1 (0-1) | 1.716 (0.811-3.056) | 1 (0-2) | 2.185 (0.838-4.179) | 1.65 (1.44 to 1.85) |
| Argentina | 218 (79-438) | 1.371 (0.499-2.753) | 296 (107-619) | 1.258 (0.453-2.629) | -0.55 (-0.93 to -0.17) |
| Armenia | 18 (7-34) | 1.063 (0.419-2.015) | 11 (5-21) | 0.74 (0.329-1.473) | -1.31 (-1.64 to -0.98) |
| Australia | 633 (348-989) | 7.041 (3.868-11.006) | 809 (433-1303) | 6.737 (3.609-10.848) | -0.07 (-0.26 to 0.12) |
| Austria | 121 (50-220) | 3.009 (1.232-5.462) | 53 (22-102) | 1.323 (0.538-2.535) | -2.46 (-2.72 to -2.2) |
| Azerbaijan | 28 (12-53) | 0.752 (0.326-1.456) | 35 (14-72) | 0.636 (0.245-1.295) | -0.61 (-0.84 to -0.38) |
| Bahamas | 4 (2-7) | 2.754 (1.213-4.954) | 9 (4-17) | 4.256 (1.678-8.08) | 1.77 (1.55 to 1.99) |
| Bahrain | 7 (4-11) | 2.224 (1.191-3.551) | 28 (14-50) | 2.866 (1.426-5.144) | 0.31 (-0.06 to 0.69) |
| Bangladesh | 241 (94-477) | 0.482 (0.187-0.954) | 519 (209-1076) | 0.59 (0.237-1.224) | 1.39 (0.88 to 1.9) |
| Barbados | 10 (5-15) | 7.279 (3.954-11.045) | 13 (7-22) | 9.345 (4.91-15.568) | 1.17 (0.68 to 1.66) |
| Belarus | 82 (35-166) | 1.63 (0.698-3.287) | 75 (31-153) | 1.761 (0.717-3.604) | -0.65 (-1.01 to -0.28) |
| Belgium | 179 (87-314) | 3.61 (1.759-6.323) | 112 (50-199) | 2.252 (0.993-3.983) | -2.07 (-2.26 to -1.87) |
| Belize | 1 (0-1) | 0.703 (0.288-1.266) | 4 (1-7) | 1.522 (0.627-2.815) | 2.76 (2.27 to 3.25) |
| Benin | 2 (0-4) | 0.083 (0.024-0.199) | 5 (2-13) | 0.085 (0.025-0.207) | -0.06 (-0.57 to 0.46) |
| Bermuda | 1 (1-2) | 3.26 (1.505-5.804) | 1 (0-2) | 3.329 (1.409-6.376) | -0.05 (-0.16 to 0.06) |
| Bhutan | 9 (4-14) | 2.779 (1.291-4.532) | 14 (7-23) | 3.252 (1.691-5.222) | -0.09 (-0.27 to 0.09) |
| Bolivia (Plurinational State of) | 34 (11-74) | 1.129 (0.371-2.474) | 76 (27-164) | 1.22 (0.433-2.617) | -0.11 (-0.34 to 0.11) |
| Bosnia and Herzegovina | 23 (10-45) | 0.971 (0.416-1.895) | 20 (8-40) | 1.372 (0.527-2.711) | 1.33 (1.08 to 1.58) |
| Botswana | 9 (4-18) | 1.548 (0.603-2.944) | 27 (11-53) | 1.955 (0.826-3.875) | 0.95 (0.57 to 1.34) |
| Brazil | 1433 (769-2363) | 1.87 (1.004-3.084) | 4912 (2528-8161) | 4.235 (2.18-7.037) | 2.71 (2.6 to 2.83) |
| Brunei Darussalam | 9 (4-15) | 5.865 (2.807-10.558) | 17 (8-29) | 6.14 (2.789-10.766) | 0.58 (0.15 to 1.01) |
| Bulgaria | 150 (63-279) | 3.599 (1.51-6.718) | 114 (49-224) | 3.869 (1.672-7.64) | 0.34 (0.06 to 0.63) |
| Burkina Faso | 8 (3-16) | 0.206 (0.076-0.429) | 21 (7-42) | 0.204 (0.071-0.405) | 0.16 (-0.21 to 0.54) |
| Burundi | 11 (4-25) | 0.467 (0.158-1.012) | 21 (7-44) | 0.33 (0.114-0.698) | -1.92 (-2.21 to -1.63) |
| Cabo Verde | 0 (0-1) | 0.244 (0.087-0.513) | 1 (1-3) | 0.436 (0.162-0.916) | 1.46 (1 to 1.92) |
| Cambodia | 16 (3-38) | 0.35 (0.075-0.832) | 31 (9-71) | 0.343 (0.104-0.793) | -0.81 (-1.28 to -0.35) |
| Cameroon | 27 (11-49) | 0.595 (0.254-1.091) | 100 (39-210) | 0.651 (0.254-1.364) | 0.41 (0.35 to 0.47) |
| Canada | 146 (47-297) | 0.988 (0.322-2.014) | 180 (58-404) | 1.084 (0.35-2.428) | 0.51 (0.29 to 0.73) |
| Central African Republic | 11 (4-23) | 0.896 (0.332-1.821) | 25 (9-53) | 0.934 (0.337-1.976) | -0.02 (-0.18 to 0.13) |
| Chad | 7 (3-14) | 0.284 (0.107-0.572) | 29 (10-63) | 0.387 (0.138-0.852) | 1.44 (1.27 to 1.61) |
| Chile | 72 (27-143) | 1.009 (0.386-2.013) | 140 (51-278) | 1.469 (0.532-2.928) | 1.69 (1.49 to 1.88) |
| China | 15064 (7717-26567) | 2.259 (1.157-3.984) | 17100 (8063-32360) | 2.578 (1.216-4.879) | 0.1 (-0.44 to 0.64) |
| Colombia | 440 (241-704) | 2.603 (1.422-4.163) | 1644 (868-2562) | 6.297 (3.325-9.809) | 3.64 (3.04 to 4.25) |
| Comoros | 1 (0-2) | 0.37 (0.118-0.825) | 2 (1-5) | 0.588 (0.198-1.328) | 1.96 (1.21 to 2.71) |
| Congo | 11 (4-20) | 0.97 (0.381-1.85) | 35 (14-76) | 1.257 (0.494-2.69) | 0.91 (0.47 to 1.35) |
| Cook Islands | 0 (0-0) | 2.347 (1.038-4.095) | 0 (0-0) | 2.15 (0.96-4.048) | 0.17 (-0.1 to 0.45) |
| Costa Rica | 13 (5-26) | 0.841 (0.341-1.686) | 56 (22-106) | 2.228 (0.862-4.223) | 3.21 (2.97 to 3.44) |
| Côte d'Ivoire | 14 (6-28) | 0.252 (0.103-0.504) | 45 (18-95) | 0.329 (0.131-0.691) | 1.26 (0.98 to 1.54) |
| Croatia | 45 (19-82) | 1.838 (0.787-3.349) | 42 (16-78) | 2.27 (0.892-4.261) | 0.99 (0.73 to 1.25) |
| Cuba | 143 (59-272) | 2.331 (0.957-4.417) | 174 (62-350) | 3.418 (1.225-6.882) | 1.52 (1.37 to 1.66) |
| Cyprus | 6 (3-11) | 1.515 (0.633-2.76) | 8 (3-16) | 1.179 (0.436-2.274) | -0.99 (-1.16 to -0.82) |
| Czechia | 153 (67-275) | 2.944 (1.291-5.286) | 129 (58-250) | 2.735 (1.237-5.301) | -0.4 (-0.83 to 0.03) |
| Democratic People's Republic of Korea | 273 (102-571) | 2.536 (0.946-5.318) | 390 (135-874) | 2.823 (0.973-6.322) | 0.6 (0.51 to 0.68) |
| Democratic Republic of the Congo | 79 (32-158) | 0.47 (0.191-0.942) | 213 (79-457) | 0.492 (0.181-1.054) | 0.17 (-0.19 to 0.54) |
| Denmark | 88 (36-159) | 3.299 (1.362-5.963) | 42 (16-87) | 1.622 (0.634-3.392) | -2.45 (-2.61 to -2.29) |
| Djibouti | 1 (0-2) | 0.416 (0.138-1.009) | 5 (2-12) | 0.672 (0.228-1.671) | 1.61 (1.47 to 1.75) |
| Dominica | 0 (0-1) | 0.85 (0.321-1.78) | 0 (0-1) | 1.322 (0.524-2.635) | 1.82 (1.63 to 2.01) |
| Dominican Republic | 41 (19-73) | 1.136 (0.526-2.006) | 115 (46-209) | 1.965 (0.791-3.571) | 2.31 (2.11 to 2.51) |
| Ecuador | 25 (10-51) | 0.513 (0.207-1.033) | 100 (40-204) | 1.062 (0.423-2.171) | 2.46 (2.14 to 2.78) |
| Egypt | 744 (400-1153) | 2.771 (1.49-4.295) | 1631 (838-2701) | 3.051 (1.568-5.053) | 0.34 (0.22 to 0.47) |
| El Salvador | 16 (6-32) | 0.65 (0.232-1.267) | 42 (15-85) | 1.256 (0.439-2.578) | 2.38 (2.19 to 2.56) |
| Equatorial Guinea | 1 (0-3) | 0.721 (0.243-1.626) | 6 (2-13) | 0.719 (0.256-1.57) | 0.24 (0.01 to 0.47) |
| Eritrea | 4 (1-9) | 0.235 (0.062-0.568) | 10 (3-27) | 0.29 (0.083-0.801) | 1 (0.61 to 1.4) |
| Estonia | 9 (3-18) | 1.198 (0.444-2.39) | 6 (2-12) | 1.039 (0.407-2.133) | -1.12 (-1.46 to -0.79) |
| Eswatini | 5 (2-8) | 1.374 (0.655-2.369) | 17 (7-33) | 2.73 (1.087-5.401) | 2.7 (2.16 to 3.25) |
| Ethiopia | 222 (65-444) | 1.021 (0.301-2.047) | 403 (160-713) | 0.732 (0.291-1.295) | -1.32 (-1.77 to -0.86) |
| Fiji | 11 (6-20) | 2.799 (1.487-4.964) | 14 (7-26) | 3.083 (1.561-5.612) | 0.5 (0.26 to 0.74) |
| Finland | 56 (24-103) | 2.168 (0.928-3.979) | 26 (10-52) | 1.108 (0.431-2.217) | -2.49 (-2.63 to -2.34) |
| France | 1009 (435-1757) | 3.459 (1.491-6.021) | 828 (371-1461) | 2.927 (1.31-5.166) | -0.57 (-0.75 to -0.38) |
| Gabon | 2 (1-4) | 0.388 (0.114-0.847) | 4 (1-10) | 0.476 (0.154-1.035) | 0.46 (0.26 to 0.65) |
| Gambia | 1 (0-2) | 0.173 (0.065-0.346) | 3 (1-6) | 0.227 (0.096-0.479) | 0.55 (0.24 to 0.86) |
| Georgia | 31 (14-57) | 1.16 (0.517-2.114) | 20 (8-38) | 1.232 (0.477-2.367) | 0.82 (0.54 to 1.09) |
| Germany | 984 (412-1967) | 2.467 (1.032-4.932) | 545 (210-1083) | 1.533 (0.589-3.043) | -1.33 (-1.61 to -1.04) |
| Ghana | 49 (20-92) | 0.723 (0.293-1.343) | 157 (65-330) | 0.898 (0.372-1.881) | 0.85 (0.17 to 1.55) |
| Greece | 54 (20-107) | 1.067 (0.399-2.11) | 54 (19-108) | 1.242 (0.428-2.488) | 0.56 (0.19 to 0.92) |
| Greenland | 1 (0-3) | 3.721 (1.269-8.259) | 1 (0-1) | 2.416 (0.838-5.314) | -1.14 (-1.63 to -0.66) |
| Grenada | 1 (0-1) | 2.083 (0.941-3.737) | 1 (1-3) | 2.731 (1.26-4.686) | 1.14 (0.9 to 1.37) |
| Guam | 1 (0-1) | 0.748 (0.34-1.42) | 1 (1-3) | 1.815 (0.802-3.462) | 3.32 (2.84 to 3.81) |
| Guatemala | 6 (2-14) | 0.17 (0.054-0.4) | 29 (8-67) | 0.349 (0.094-0.802) | 2.55 (1.9 to 3.2) |
| Guinea | 5 (2-10) | 0.197 (0.067-0.407) | 11 (4-24) | 0.18 (0.058-0.395) | -0.4 (-0.6 to -0.19) |
| Guinea-Bissau | 3 (1-7) | 0.771 (0.265-1.503) | 9 (4-18) | 0.895 (0.352-1.823) | 0.62 (0.49 to 0.75) |
| Guyana | 5 (2-9) | 1.207 (0.512-2.197) | 10 (4-21) | 2.529 (0.92-5.166) | 2.63 (2.11 to 3.16) |
| Haiti | 69 (22-136) | 2.354 (0.735-4.648) | 159 (54-323) | 2.322 (0.785-4.709) | 0.09 (0 to 0.19) |
| Honduras | 13 (5-25) | 0.621 (0.247-1.229) | 38 (13-88) | 0.704 (0.239-1.617) | 0.12 (-0.08 to 0.32) |
| Hungary | 143 (65-272) | 2.808 (1.285-5.336) | 129 (52-247) | 2.968 (1.209-5.7) | -0.13 (-0.32 to 0.06) |
| Iceland | 3 (1-5) | 1.956 (0.79-3.896) | 3 (1-5) | 1.654 (0.671-3.126) | -1.23 (-1.47 to -0.99) |
| India | 3296 (1711-5339) | 0.784 (0.407-1.27) | 5632 (2903-9092) | 0.722 (0.372-1.166) | -0.73 (-0.96 to -0.5) |
| Indonesia | 2364 (1162-4039) | 2.501 (1.229-4.273) | 5439 (2622-9804) | 3.547 (1.71-6.394) | 1.37 (1.3 to 1.44) |
| Iran (Islamic Republic of) | 584 (301-915) | 2.286 (1.178-3.584) | 1459 (813-2165) | 3.078 (1.714-4.567) | 1.32 (0.91 to 1.74) |
| Iraq | 258 (136-415) | 3.048 (1.601-4.898) | 777 (383-1333) | 3.509 (1.73-6.016) | 0.75 (0.61 to 0.88) |
| Ireland | 60 (28-109) | 3.388 (1.566-6.128) | 57 (26-100) | 2.475 (1.128-4.334) | -1.08 (-1.25 to -0.9) |
| Israel | 52 (20-96) | 2.155 (0.825-3.942) | 73 (29-144) | 1.614 (0.655-3.201) | -1.15 (-1.47 to -0.84) |
| Italy | 1319 (678-2080) | 4.593 (2.359-7.241) | 884 (399-1453) | 3.592 (1.622-5.903) | -0.71 (-0.87 to -0.54) |
| Jamaica | 27 (15-43) | 2.361 (1.287-3.654) | 85 (44-139) | 5.532 (2.878-9.075) | 2.94 (2.36 to 3.52) |
| Japan | 6490 (3675-9914) | 9.997 (5.661-15.273) | 4032 (2157-6589) | 7.955 (4.256-13.002) | -0.7 (-0.97 to -0.43) |
| Jordan | 38 (18-64) | 2.123 (1.025-3.572) | 131 (57-247) | 1.915 (0.839-3.613) | -0.24 (-0.37 to -0.11) |
| Kazakhstan | 113 (53-198) | 1.379 (0.648-2.414) | 108 (44-205) | 1.157 (0.466-2.194) | -0.44 (-0.63 to -0.25) |
| Kenya | 87 (41-148) | 0.856 (0.4-1.453) | 340 (164-567) | 1.302 (0.628-2.17) | 1.64 (1.51 to 1.77) |
| Kiribati | 2 (1-3) | 4.607 (2.463-7.399) | 3 (2-5) | 5.177 (2.761-8.806) | 0.19 (0.03 to 0.35) |
| Kuwait | 32 (18-47) | 3.135 (1.795-4.598) | 222 (127-338) | 7.275 (4.169-11.092) | 1.75 (1.04 to 2.46) |
| Kyrgyzstan | 18 (6-35) | 0.851 (0.31-1.649) | 23 (9-47) | 0.662 (0.248-1.378) | -0.98 (-1.14 to -0.82) |
| Lao People's Democratic Republic | 12 (3-28) | 0.668 (0.184-1.523) | 25 (8-57) | 0.628 (0.203-1.431) | -0.42 (-0.9 to 0.07) |
| Latvia | 37 (16-67) | 2.87 (1.245-5.185) | 18 (8-36) | 2.338 (0.98-4.526) | -0.91 (-1.14 to -0.68) |
| Lebanon | 66 (31-116) | 4.598 (2.152-8.078) | 110 (58-185) | 3.661 (1.908-6.15) | -0.58 (-0.82 to -0.35) |
| Lesotho | 1 (0-3) | 0.172 (0.048-0.41) | 3 (1-8) | 0.334 (0.109-0.761) | 2.54 (2.03 to 3.05) |
| Liberia | 10 (5-18) | 0.938 (0.431-1.626) | 40 (17-78) | 1.438 (0.6-2.765) | 1.07 (0.67 to 1.46) |
| Libya | 76 (36-129) | 3.828 (1.836-6.515) | 272 (122-474) | 6.606 (2.968-11.507) | 2.44 (2.18 to 2.7) |
| Lithuania | 43 (20-77) | 2.336 (1.106-4.191) | 28 (12-52) | 2.439 (1.002-4.538) | 0.39 (0.06 to 0.71) |
| Luxembourg | 6 (3-11) | 3.225 (1.453-5.699) | 4 (2-8) | 1.38 (0.556-2.658) | -2.78 (-2.94 to -2.61) |
| Madagascar | 25 (7-60) | 0.464 (0.137-1.132) | 63 (21-149) | 0.444 (0.146-1.054) | -0.02 (-0.14 to 0.11) |
| Malawi | 6 (2-12) | 0.138 (0.048-0.27) | 14 (5-31) | 0.145 (0.049-0.317) | 0 (-0.32 to 0.33) |
| Malaysia | 228 (110-398) | 2.528 (1.218-4.419) | 784 (339-1273) | 4.415 (1.907-7.174) | 2.13 (1.67 to 2.58) |
| Maldives | 4 (1-7) | 4.174 (1.2-7.35) | 7 (4-11) | 2.021 (1.067-3.3) | -2.53 (-2.7 to -2.36) |
| Mali | 23 (11-43) | 0.644 (0.314-1.18) | 60 (24-125) | 0.575 (0.23-1.188) | -0.13 (-0.39 to 0.14) |
| Malta | 7 (4-13) | 3.765 (1.882-6.64) | 7 (3-12) | 3.611 (1.641-6.099) | -0.89 (-1.17 to -0.61) |
| Marshall Islands | 2 (1-3) | 9.154 (4.857-14.208) | 4 (2-7) | 14.294 (7.212-23.342) | 1.25 (1.08 to 1.42) |
| Mauritania | 13 (6-21) | 1.409 (0.629-2.299) | 33 (18-60) | 1.626 (0.857-2.904) | 0.61 (0.49 to 0.73) |
| Mauritius | 4 (2-8) | 0.699 (0.3-1.324) | 12 (5-23) | 1.93 (0.848-3.669) | 3.03 (2.64 to 3.41) |
| Mexico | 239 (114-411) | 0.561 (0.267-0.967) | 1148 (512-2054) | 1.677 (0.748-3) | 4.01 (3.29 to 4.73) |
| Micronesia (Federated States of) | 5 (3-8) | 10.276 (5.382-16.857) | 7 (3-11) | 12.292 (6.454-20.179) | 0.56 (0.47 to 0.64) |
| Monaco | 1 (0-1) | 4.566 (1.658-9.032) | 1 (0-1) | 4.995 (1.769-10.361) | 0.5 (0.32 to 0.68) |
| Mongolia | 5 (2-9) | 0.448 (0.176-0.886) | 9 (4-21) | 0.561 (0.21-1.216) | 0.23 (-0.11 to 0.58) |
| Montenegro | 4 (2-9) | 1.351 (0.525-2.761) | 4 (2-9) | 1.478 (0.556-2.918) | 0.39 (-0.06 to 0.85) |
| Morocco | 303 (135-570) | 2.433 (1.087-4.58) | 783 (326-1382) | 4.033 (1.678-7.115) | 1.8 (1.66 to 1.94) |
| Mozambique | 5 (2-10) | 0.084 (0.027-0.171) | 13 (4-29) | 0.094 (0.028-0.206) | 0.94 (0.68 to 1.19) |
| Myanmar | 65 (16-163) | 0.32 (0.08-0.801) | 111 (29-275) | 0.377 (0.098-0.935) | 0.66 (0.05 to 1.27) |
| Namibia | 6 (3-10) | 0.875 (0.413-1.524) | 16 (8-32) | 1.259 (0.586-2.444) | 0.96 (0.68 to 1.24) |
| Nauru | 0 (0-1) | 6.449 (2.267-12.38) | 0 (0-1) | 6.084 (2.358-11.855) | -0.36 (-0.46 to -0.26) |
| Nepal | 34 (11-73) | 0.38 (0.123-0.815) | 70 (24-158) | 0.423 (0.144-0.947) | 0.66 (0 to 1.32) |
| Netherlands | 133 (54-261) | 1.644 (0.665-3.221) | 96 (32-206) | 1.291 (0.437-2.778) | -0.85 (-1.15 to -0.54) |
| New Zealand | 58 (25-110) | 3.201 (1.404-6.116) | 92 (44-163) | 3.79 (1.809-6.707) | 1.56 (1.1 to 2.02) |
| Nicaragua | 8 (3-16) | 0.483 (0.198-0.929) | 26 (11-52) | 0.72 (0.293-1.449) | 1.66 (1.46 to 1.86) |
| Niger | 13 (5-27) | 0.401 (0.162-0.809) | 36 (13-78) | 0.348 (0.126-0.752) | -0.21 (-0.33 to -0.09) |
| Nigeria | 222 (100-400) | 0.54 (0.245-0.976) | 623 (268-1155) | 0.578 (0.248-1.071) | 0.51 (0.38 to 0.64) |
| Niue | 0 (0-0) | 3.579 (1.563-6.855) | 0 (0-0) | 4.291 (2.055-7.786) | 0.18 (0.06 to 0.3) |
| North Macedonia | 16 (7-31) | 1.553 (0.651-2.96) | 16 (6-32) | 1.476 (0.581-2.949) | -0.34 (-0.71 to 0.04) |
| Northern Mariana Islands | 1 (1-2) | 4.409 (2.084-7.86) | 1 (1-2) | 5.297 (2.447-9.249) | 0.82 (0.54 to 1.09) |
| Norway | 80 (39-142) | 3.68 (1.807-6.563) | 59 (27-105) | 2.371 (1.09-4.185) | -1.62 (-1.76 to -1.47) |
| Oman | 8 (4-15) | 0.82 (0.384-1.495) | 21 (10-36) | 0.708 (0.326-1.209) | -0.55 (-0.73 to -0.37) |
| Pakistan | 362 (164-615) | 0.733 (0.333-1.244) | 1249 (574-2203) | 1.024 (0.471-1.806) | 0.82 (0.72 to 0.93) |
| Palau | 0 (0-1) | 5.635 (2.772-9.914) | 1 (0-1) | 6.08 (2.823-10.834) | 0.12 (0.01 to 0.24) |
| Palestine | 57 (28-98) | 6.376 (3.212-11.025) | 133 (71-200) | 5.004 (2.659-7.547) | -0.73 (-0.82 to -0.63) |
| Panama | 10 (4-20) | 0.847 (0.317-1.628) | 33 (11-63) | 1.517 (0.531-2.916) | 2.54 (2.31 to 2.78) |
| Papua New Guinea | 32 (15-58) | 1.614 (0.733-2.924) | 84 (41-156) | 1.58 (0.762-2.918) | -0.26 (-0.39 to -0.13) |
| Paraguay | 8 (3-16) | 0.407 (0.146-0.831) | 27 (9-60) | 0.707 (0.243-1.565) | 2.18 (1.97 to 2.39) |
| Peru | 82 (33-158) | 0.763 (0.308-1.473) | 188 (63-403) | 0.972 (0.325-2.087) | 0.83 (0.64 to 1.02) |
| Philippines | 272 (122-486) | 0.875 (0.394-1.561) | 811 (357-1471) | 1.351 (0.595-2.45) | 1.76 (1.51 to 2.01) |
| Poland | 596 (304-993) | 3.145 (1.603-5.235) | 568 (273-1046) | 3.169 (1.524-5.835) | -0.5 (-0.7 to -0.3) |
| Portugal | 170 (79-301) | 3.405 (1.586-6.028) | 193 (76-371) | 4.211 (1.666-8.101) | 0.95 (0.79 to 1.12) |
| Puerto Rico | 39 (16-71) | 2.107 (0.851-3.844) | 39 (18-73) | 2.688 (1.261-4.963) | 0.61 (0.42 to 0.8) |
| Qatar | 10 (5-16) | 3.326 (1.755-5.398) | 56 (28-96) | 2.567 (1.294-4.377) | -1.6 (-2.03 to -1.17) |
| Republic of Korea | 948 (448-1602) | 3.664 (1.731-6.194) | 895 (406-1626) | 3.686 (1.673-6.693) | -0.11 (-0.34 to 0.13) |
| Republic of Moldova | 34 (14-62) | 1.555 (0.648-2.841) | 26 (10-49) | 1.469 (0.531-2.744) | -0.34 (-0.61 to -0.07) |
| Romania | 274 (128-503) | 2.418 (1.133-4.44) | 289 (132-530) | 3.465 (1.588-6.367) | 0.86 (0.55 to 1.16) |
| Russian Federation | 1113 (477-1990) | 1.499 (0.642-2.682) | 1095 (485-2006) | 1.624 (0.718-2.974) | -0.32 (-0.56 to -0.07) |
| Rwanda | 18 (6-40) | 0.582 (0.182-1.259) | 27 (8-59) | 0.398 (0.115-0.856) | -2.5 (-3.02 to -1.97) |
| Saint Kitts and Nevis | 0 (0-1) | 2.159 (0.951-3.745) | 1 (0-1) | 1.959 (0.744-4.172) | -0.5 (-0.7 to -0.29) |
| Saint Lucia | 1 (0-2) | 1.475 (0.607-2.835) | 2 (1-4) | 2.165 (0.877-4.233) | 1.72 (1.49 to 1.95) |
| Saint Vincent and the Grenadines | 1 (0-1) | 1.357 (0.598-2.445) | 2 (1-3) | 2.935 (1.266-5.672) | 2.62 (2.4 to 2.84) |
| Samoa | 3 (2-5) | 4.236 (2.298-6.771) | 6 (3-10) | 6.278 (3.087-10.377) | 1.46 (1.36 to 1.55) |
| San Marino | 0 (0-1) | 2.351 (0.896-4.444) | 0 (0-1) | 1.962 (0.703-4.202) | 0.73 (0.21 to 1.25) |
| Sao Tome and Principe | 0 (0-0) | 0.243 (0.085-0.484) | 0 (0-1) | 0.339 (0.117-0.753) | 0.9 (0.69 to 1.1) |
| Saudi Arabia | 133 (64-253) | 1.653 (0.804-3.154) | 980 (519-1656) | 3.873 (2.051-6.547) | 3.23 (3.03 to 3.43) |
| Senegal | 15 (6-27) | 0.452 (0.183-0.838) | 45 (17-88) | 0.578 (0.221-1.138) | 1.22 (0.91 to 1.52) |
| Serbia | 115 (47-221) | 2.413 (0.994-4.638) | 83 (31-166) | 1.963 (0.733-3.923) | -1.09 (-1.42 to -0.76) |
| Seychelles | 1 (0-1) | 1.563 (0.62-3.091) | 2 (1-3) | 2.782 (1.03-5.975) | 2.34 (2.04 to 2.63) |
| Sierra Leone | 6 (3-12) | 0.326 (0.138-0.607) | 21 (8-42) | 0.467 (0.188-0.954) | 1.41 (1.31 to 1.52) |
| Singapore | 88 (43-146) | 4.622 (2.254-7.691) | 89 (44-153) | 2.997 (1.472-5.123) | -2.03 (-2.51 to -1.55) |
| Slovakia | 66 (29-125) | 2.488 (1.076-4.679) | 57 (21-110) | 2.215 (0.823-4.237) | -0.47 (-0.65 to -0.28) |
| Slovenia | 20 (9-37) | 2.01 (0.866-3.601) | 13 (5-25) | 1.48 (0.569-2.902) | -1.25 (-1.45 to -1.05) |
| Solomon Islands | 4 (2-8) | 2.824 (1.091-5.597) | 15 (7-27) | 4.441 (1.992-7.971) | 1.7 (1.53 to 1.87) |
| Somalia | 20 (6-47) | 0.561 (0.159-1.353) | 46 (12-105) | 0.461 (0.118-1.056) | -1.3 (-1.53 to -1.07) |
| South Africa | 860 (488-1278) | 4.559 (2.586-6.776) | 1603 (889-2468) | 5.146 (2.854-7.922) | 0.57 (0.26 to 0.88) |
| South Sudan | 10 (3-24) | 0.375 (0.124-0.876) | 24 (8-57) | 0.55 (0.182-1.272) | 1.41 (0.94 to 1.88) |
| Spain | 573 (279-1066) | 2.953 (1.439-5.491) | 518 (213-956) | 2.59 (1.063-4.783) | -0.37 (-0.5 to -0.23) |
| Sri Lanka | 25 (9-52) | 0.269 (0.098-0.569) | 40 (12-84) | 0.364 (0.11-0.766) | 1.73 (1.36 to 2.11) |
| Sudan | 767 (370-1356) | 8.422 (4.057-14.881) | 1984 (931-3428) | 8.872 (4.162-15.333) | 0.37 (0.29 to 0.45) |
| Suriname | 7 (3-11) | 3.432 (1.555-5.761) | 16 (8-27) | 5.683 (2.853-9.351) | 1.83 (1.62 to 2.04) |
| Sweden | 102 (44-198) | 2.435 (1.039-4.713) | 91 (44-159) | 2.017 (0.965-3.512) | 0.18 (-0.1 to 0.47) |
| Switzerland | 79 (32-147) | 2.177 (0.881-4.057) | 52 (21-99) | 1.298 (0.535-2.464) | -1.59 (-1.84 to -1.35) |
| Syrian Arab Republic | 151 (67-252) | 2.7 (1.194-4.495) | 216 (103-381) | 3.044 (1.447-5.372) | 0.62 (0.03 to 1.21) |
| Taiwan (Province of China) | 844 (446-1372) | 7.496 (3.959-12.184) | 1339 (669-2250) | 11.772 (5.881-19.783) | 1.23 (1.04 to 1.43) |
| Tajikistan | 13 (6-27) | 0.559 (0.232-1.105) | 20 (7-44) | 0.396 (0.145-0.852) | -1.51 (-1.84 to -1.18) |
| Thailand | 356 (150-711) | 1.125 (0.473-2.245) | 799 (276-1700) | 2.504 (0.865-5.327) | 2.34 (1.95 to 2.73) |
| Timor-Leste | 2 (1-5) | 0.514 (0.164-1.231) | 3 (1-7) | 0.458 (0.178-0.984) | -0.93 (-1.39 to -0.47) |
| Togo | 2 (1-5) | 0.141 (0.047-0.301) | 8 (3-18) | 0.193 (0.066-0.426) | 0.95 (0.64 to 1.26) |
| Tokelau | 0 (0-0) | 3.599 (1.605-6.884) | 0 (0-0) | 4.239 (2.118-7.016) | 0.11 (-0.07 to 0.3) |
| Tonga | 1 (1-2) | 2.57 (1.308-4.261) | 1 (1-2) | 2.831 (1.492-4.988) | 0.31 (0.25 to 0.37) |
| Trinidad and Tobago | 24 (13-39) | 3.953 (2.113-6.306) | 46 (23-79) | 6.612 (3.381-11.419) | 1.53 (1.34 to 1.72) |
| Tunisia | 21 (9-38) | 0.506 (0.218-0.919) | 55 (21-104) | 0.914 (0.346-1.716) | 2.06 (1.89 to 2.23) |
| Turkey | 1055 (494-1902) | 3.641 (1.703-6.564) | 1280 (578-2246) | 2.913 (1.317-5.112) | -0.73 (-1.23 to -0.23) |
| Turkmenistan | 8 (3-16) | 0.455 (0.198-0.883) | 15 (6-30) | 0.575 (0.208-1.113) | 0.44 (-0.04 to 0.91) |
| Tuvalu | 0 (0-0) | 4.633 (2.05-8.466) | 0 (0-0) | 3.704 (1.832-6.452) | -1.01 (-1.15 to -0.87) |
| Uganda | 26 (10-60) | 0.355 (0.131-0.811) | 90 (30-192) | 0.446 (0.15-0.956) | -0.08 (-0.43 to 0.28) |
| Ukraine | 432 (187-857) | 1.731 (0.748-3.436) | 284 (102-577) | 1.406 (0.502-2.853) | -1.26 (-1.53 to -0.99) |
| United Arab Emirates | 48 (21-85) | 4.074 (1.742-7.252) | 221 (96-384) | 3.213 (1.4-5.601) | -1.09 (-1.5 to -0.68) |
| United Kingdom | 2502 (1456-3611) | 8.79 (5.115-12.688) | 2274 (1294-3295) | 7.493 (4.266-10.859) | -0.42 (-0.64 to -0.21) |
| United Republic of Tanzania | 16 (5-41) | 0.143 (0.042-0.363) | 35 (11-79) | 0.125 (0.039-0.279) | -0.97 (-1.3 to -0.64) |
| United States of America | 2503 (1145-4312) | 1.864 (0.853-3.212) | 3624 (1752-5991) | 2.384 (1.153-3.941) | 0.95 (0.57 to 1.33) |
| United States Virgin Islands | 1 (0-2) | 2.106 (0.816-4.353) | 1 (0-1) | 1.643 (0.55-3.53) | 0.2 (-0.14 to 0.53) |
| Uruguay | 47 (21-89) | 3.184 (1.407-6.007) | 70 (30-132) | 4.258 (1.851-8.017) | 1.24 (1.04 to 1.44) |
| Uzbekistan | 27 (11-52) | 0.28 (0.11-0.534) | 70 (31-128) | 0.394 (0.175-0.72) | 1.38 (0.98 to 1.78) |
| Vanuatu | 0 (0-1) | 0.308 (0.093-0.748) | 0 (0-1) | 0.309 (0.094-0.792) | -1.01 (-1.51 to -0.52) |
| Venezuela (Bolivarian Republic of) | 83 (32-152) | 0.867 (0.339-1.583) | 226 (86-453) | 1.717 (0.654-3.445) | 2.07 (1.87 to 2.27) |
| Viet Nam | 193 (77-388) | 0.588 (0.234-1.185) | 849 (322-1825) | 1.63 (0.618-3.505) | 4.16 (3.82 to 4.5) |
| Yemen | 116 (48-231) | 2.141 (0.89-4.246) | 408 (167-760) | 2.442 (0.998-4.543) | 0.43 (0.29 to 0.58) |
| Zambia | 23 (10-45) | 0.657 (0.271-1.266) | 84 (26-229) | 0.873 (0.267-2.366) | 1.02 (0.54 to 1.5) |
| Zimbabwe | 23 (9-46) | 0.499 (0.205-0.989) | 96 (36-203) | 1.244 (0.459-2.616) | 3.92 (3.24 to 4.6) |

ASR, age-standardized rate; EAPC, estimated annual percentage change; CI, confidence interval.
